# Evaluating School‐Based Obesity Prevention Interventions in 6‐ to 12‐Year‐Old Children: A Scoping Review of All Reported Outcomes and Expert Consultation

**DOI:** 10.1111/obr.70125

**Published:** 2026-03-12

**Authors:** Teatske M. Altenburg, Lotte W. de Vries, Iris J. Grooten, Janneke van 't Hooft, Arend W. van Deutekom, Tessa Roseboom, Erica van den Akker, Michael Duncan, Ulf Ekelund, John J. Reilly, Lisa Reilly, Annette Stafleu, Martyn Standage, Mai J. M. Chinapaw, Deirdre M. Harrington

**Affiliations:** ^1^ Public and Occupational Health Amsterdam UMC, Location Vrije Universiteit Amsterdam Amsterdam the Netherlands; ^2^ Health Behaviours and Chronic Disease Amsterdam Public Health Amsterdam the Netherlands; ^3^ Methodology Amsterdam Public Health Amsterdam the Netherlands; ^4^ Department of Obstetrics and Gynaecology Amsterdam UMC, University of Amsterdam Amsterdam the Netherlands; ^5^ Amsterdam Reproduction and Development Institute Amsterdam UMC, University of Amsterdam Amsterdam the Netherlands; ^6^ Department of Pediatrics, Division of Pediatric Cardiology Erasmus MC–Sophia Children's Hospital Rotterdam the Netherlands; ^7^ Department of Epidemiology and Data Science Amsterdam UMC Amsterdam the Netherlands; ^8^ Division of Pediatric Endocrinology, Obesity Center CGG, Department of Pediatrics Erasmus University Medical Center–Sophia Children's Hospital Rotterdam the Netherlands; ^9^ Centre for Physical Activity, Sport and Exercise Science Coventry University Coventry UK; ^10^ Department of Sport Medicine Norwegian School of Sport Sciences Oslo Norway; ^11^ Department of Chronic Diseases Norwegian Institute of Public Health Oslo Norway; ^12^ Psychological Sciences and Health University of Strathclyde Glasgow UK; ^13^ Tayside Healthy Weight Service NHS Tayside Nutrition & Dietetics Dundee UK; ^14^ The Netherlands Nutrition Centre The Hague the Netherlands; ^15^ Department for Health University of Bath Bath UK; ^16^ Diabetes Research Centre University of Leicester Leicester UK

**Keywords:** children, core outcome set, obesity prevention, school‐based program

## Abstract

**Introduction:**

This scoping review aims to identify all outcomes reported in school‐based obesity prevention interventions in childhood. It serves as an essential first step towards developing an internationally agreed‐upon Core Outcome Set (COS), which defines *what* should be measured in all school‐based childhood obesity prevention studies, thereby reducing research waste and enhancing the comparability and relevance of future research.

**Methods:**

Four databases (PubMed, Embase, Cochrane Database of Systematic Reviews, and PsycINFO) were searched for published studies on controlled trials of school‐based overweight/obesity prevention interventions in 6‐ to 12‐year‐olds, from inception until June 2024. Two researchers independently searched for relevant articles, extracted study/intervention characteristics, and reported outcomes. Through multiple meetings and feedback rounds, an international expert panel, including researchers (*n* = 5), healthcare providers (*n* = 4; i.e., pediatrician, youth health physician, dietician, psychologist), and a health educator identified unique outcomes underlying all reported outcomes, by reflecting on what was measured irrespective of how outcomes were defined and measured.

**Results:**

In total, 262 published studies that evaluated 242 interventions were included in this review. From these studies, we extracted 642 different reported outcomes. BMI (kg/m^2^) was the most frequently reported outcome (128 studies), then BMI‐z (108 studies) and BMI categories (100 studies). Experts identified 69 unique outcomes from all reported outcomes.

**Conclusion:**

There is substantial heterogeneity in outcomes reported in studies evaluating school‐based overweight/obesity prevention interventions in 6‐ to 12‐year‐olds, limiting a synthesis of evidence in meta‐analyses. This highlights the need for a consensus‐based COS to improve the comparability and relevance of evidence of childhood obesity prevention trials.

**Abbreviations**
BMI: body mass index
COS: Core Outcome Set
EBRBs: energy balance related behaviors
PRISMA: Preferred Reporting Items for Systematic Reviews and Meta‐Analysis
UK: United Kingdom
USA: United States of America

## 1 Introduction

The prevalence of overweight (including obesity) among children has persistently increased worldwide during the last decades, in both developed and developing countries [1, 2]. These trends are worrisome, as being overweight has an adverse effect on children's physical and mental health [3]. In addition, being overweight as a child has the tendency to track into adulthood [4]. Six out of 10 children who are overweight at age 5–7 are overweight at age 15–17 [5]. Therefore, prevention of overweight in childhood is an international public health priority.

The causes of overweight in children are complex and include, among others, the obesogenic environment leading to unhealthy diet and insufficient physical activity [6, 7, 8, 9, 10, 11]. Therefore, hundreds of interventions stimulating a healthy diet and physical activity in children have been developed and tested over many years [12, 13, 14]. As most children can be reached via schools, this context has formed a convenient and feasible setting for overweight prevention. Schools can provide a variety of opportunities for interventions throughout the day (e.g., physical activity opportunities during recess and after‐school programs, free school lunches) [15, 16]. Moreover, school settings also provide an infrastructure and environment wherein interventions can be delivered to positively influence children's health behaviors [16, 17, 18].

A wide range of school‐based interventions targeting the prevention of overweight among children are implemented in various countries internationally. However, the lack of a standardized set of outcomes has led to substantial heterogeneity in reported outcomes in terms of how reported outcomes are defined and measured. For example, to assess children's body composition, outcomes such as BMI, body fat, and skinfold thickness are reported, reflecting different ways to measure body composition (i.e., ratio of weight to height, bioelectrical impedance analysis [BIA], and thickness of multiple skinfolds using calipers, respectively). Despite the potential of statistical analyses to combine different outcomes such as BMI, BMI percentiles, and BMI‐z [19], this heterogeneity in reported outcomes remains and impedes comparability across studies and the synthesis of evidence from these publications in systematic reviews and meta‐analyses [16, 17, 20]. A Core Outcome Set (COS), defined as an agreed minimum set of outcomes that should be measured and reported in all studies within a specific area, would address these challenges [21]. Developing and implementing a COS for school‐based interventions targeting the prevention of overweight in children is essential to reduce outcome reporting bias, enhance the consistency and relevance of reported outcomes, and ultimately improve the comparability, utility, and impact of the research in this field [21, 22].

The first step in developing such a COS is to obtain consensus on *what* should be measured and reported in all studies evaluating school‐based interventions targeting the prevention of overweight in children, irrespective of *how* to measure the outcomes included in the COS. To guide the consensus process on what should be measured, this scoping review aims to explore the scope of outcomes used in recent publications evaluating school‐based interventions targeting the prevention of overweight in children aged 6–12 years [22]. Specifically, this scoping review aims to (1) summarize outcomes currently reported in publications evaluating school‐based interventions designed to prevent overweight among children and (2) identify all unique outcomes underlying the reported outcomes by disregarding how outcomes were defined and measured. The resulting list of outcomes will be taken forward and presented in an international Delphi study aimed at reaching consensus on which outcomes should be included in a COS for school‐based intervention studies focused on preventing overweight (including obesity) in children [22].

## 2 Methods

The scoping review was registered in Open Science Frameworks (registration DOI 10.17605/OSF.IO/AQTYU), and the protocol for the overall COS development study is registered in the COMET database (http://www.comet‐initiative.org/studies/details/971). We followed the Preferred Reporting Items for Systematic Reviews and Meta‐Analysis (PRISMA) statement [23] using the extension for scoping reviews [24].

### 2.1 Literature Search and Eligibility Criteria

We searched four databases (PubMed, Embase, Cochrane Database of Systematic Reviews, and PsycINFO) from inception to June 2024. The full search strategy can be found in File S1. The search contains terms related to children (e.g., child, youth), prevention (e.g., health promotion), overweight (e.g., body weight, obesity), and controlled trials (e.g., controlled trials, clinical trials). Published studies were included if they (i) evaluated school‐based interventions aimed at the prevention of overweight (including obesity); (ii) included children and adolescents aged between 6 and 12 years—which we refer to as *children* from this point onward, both at baseline as well as at follow‐up measurement; and (iii) used a controlled design (randomized or non‐randomized). Only full‐text articles published in English in a peer‐reviewed journal were included. Studies in children with a disease or disorder (e.g., attention deficit disorder, autism) were excluded.

### 2.2 Selection Procedure

First, two pairs of reviewers (LV/DH and AD/TA) independently checked all identified titles and abstracts to establish potentially relevant studies. Additionally, these pairs of reviewers checked the reference lists of reviews identified by the search in the Cochrane Database of Systematic Reviews. Disagreements between reviewers were resolved through discussion, and full‐text papers were obtained for publications meeting the inclusion criteria during the initial screening. Second, two reviewers (LV/DH and AD/TA) independently screened all full‐text papers to determine if the inclusion criteria were met. Whenever necessary, a third reviewer was consulted. Disagreements were resolved through discussion.

### 2.3 Data Extraction Process

Two reviewers independently extracted data from the included studies (LV/DH and AD/TA) using a standardized data extraction sheet. Information was extracted regarding study design, study population, type and focus of intervention(s), reported outcomes, and measurement tools. Disagreements were solved through discussion. To provide a clear overview of reported outcomes, we merged similar outcomes (e.g., BMI z‐score and BMI SDs; consumption of unhealthy snacks and consumption of energy‐dense snacks) and grouped outcomes in domains (e.g., anthropometric measurements, 24‐h movement behaviors). Two reviewers (LV and TA) merged and grouped outcomes, which were checked by a third reviewer (DH).

### 2.4 Identification of Unique Outcomes

An expert panel was formed to critically review all outcomes reported in the included studies and the identify unique outcomes underlying these outcomes, by reflecting on *what* was measured irrespective of *how* outcomes were defined and measured. For example, experts discussed that the reported outcomes BMI‐z and body fat percentage are two different measures of the unique outcome body composition. We chose the word “unique” as the grouping of outcomes are unique to this review. Existing outcome taxonomy did not allow sufficient detail that was needed for this review, and therefore, a more granular process was required. The expert panel initially consisted of five members from the Netherlands with expertise in the prevention of childhood overweight (AS, EvdA, LS, LvH, MC), including two researchers, two healthcare providers (i.e., a pediatrician, a youth health physician), and one health educator (see de Vries et al. [22] for more details). The expert panel members were recruited via email from the authors' network. In‐person meetings were organized with the expert panel members, during which the list of outcomes identified through the scoping review was reduced to a comprehensive list of unique outcomes. An independent facilitator (IG) experienced in COS development guided the in‐person meetings with the Dutch members. The experts participated in multiple meetings and feedback rounds via e‐mail to reach a consensus on the unique outcomes underlying all reported outcomes. Two meetings (with five and three Dutch experts, respectively) and multiple feedback rounds via email were required to identify the first list of unique outcomes underlying all reported outcomes. After that, we grouped the unique outcomes by outcome domain, where applicable, coherent with the taxonomy proposed by Dodd et al. [25].

Once a first list of unique outcomes was identified, five international members were added to the expert panel to reflect and provide feedback on this list of outcomes. These additional members consisted of three researchers and two healthcare providers (i.e., a dietician, a psychologist) (JR, LR, MD, MS, UE). During individual online meetings, these international experts reflected and provided feedback on the list of unique outcomes within their domain of expertise. An updated list of unique outcomes was established from this input and feedback. Thereafter, TA and DH provided a draft working description for each unique outcome, based on commonly used definitions as well as descriptions in the literature and input from the expert panel members. This list of updated unique outcomes with draft working descriptions was subsequently circulated via email to all experts for further feedback. Based on all feedback, a final list of unique outcomes, working descriptions, and examples of reported outcomes was completed.

## 3 Results

### 3.1 Study Selection

**FIGURE 1:**
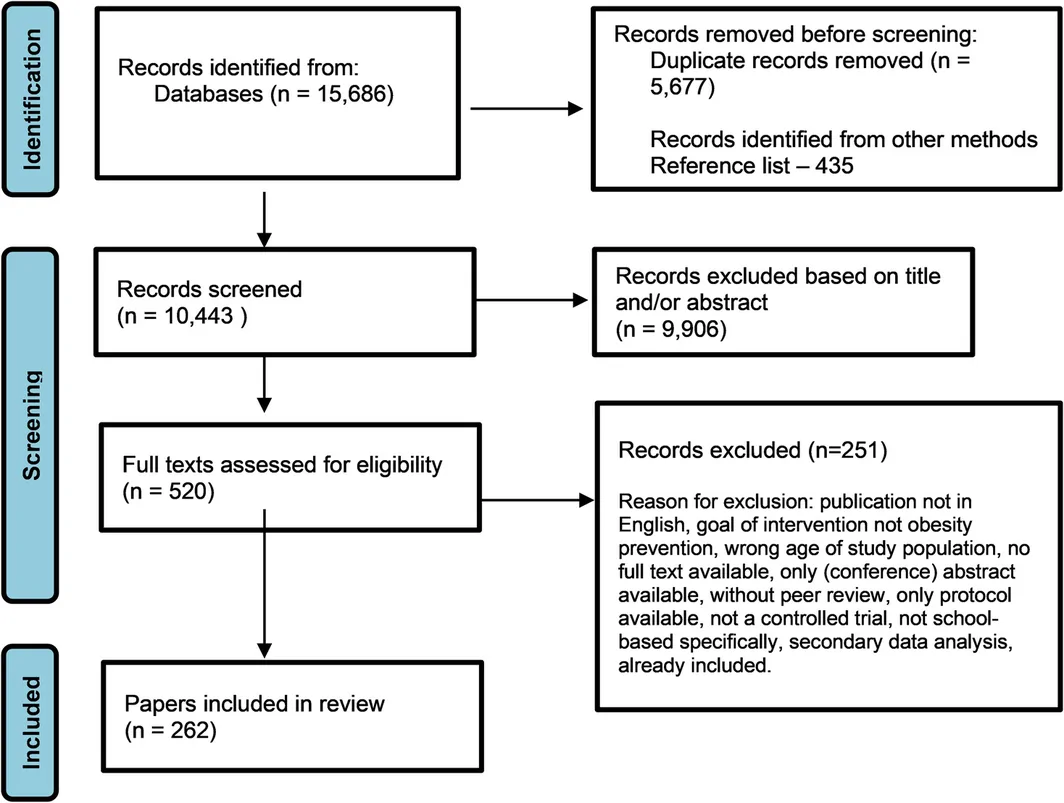
PRISMA flow chart study selection process.

The PRISMA flowchart in Figure 1 illustrates the record identification screening and inclusion process. After removing duplicates, the literature searches yielded 15,685 records, and after screening titles and abstracts, 504 full‐text articles were checked for eligibility. In addition, we screened the reference list of one relevant Cochrane review identified through the Cochrane Database. This yielded 435 additional records. The full texts of 16 of these were checked, and seven met the inclusion criteria. In total, we included 262 published studies that evaluated 242 interventions in the present scoping review. File S2 shows the complete list of studies and data extraction. File S3 shows the reference list of all included studies.

### 3.2 Study Characteristics

The 262 included intervention studies were conducted in 45 different countries, with the majority from the United States (58 studies), the United Kingdom (15 studies), China (10 studies), and Germany (nine studies), including intervention durations ranging from 1 month to 3 school years. Studies included 11–5727 participants (0%–100% girls) in the intervention group and 15–5314 participants (0%–100% girls) in the control group, with an average age of 6.0–12.3 years in the intervention group and 5.9–12.4 years in the control group.

### 3.3 Reported Outcomes

File S4 shows all (merged) reported outcomes, and Table 1 shows the most frequently reported outcomes, as well as the frequency with which they were reported and the domains in which they were grouped. The 262 studies reported 646 different outcomes, with studies reporting between 1 and 57 outcomes. BMI (kg/m^2^) was the most frequently reported outcome (128 studies), followed by BMI‐z (108 studies) and BMI categories (100 studies). Fifty‐one studies reported both BMI (kg/m^2^) and BMI‐z, and 18 studies reported BMI (kg/m^2^), BMI‐z, and BMI categories. Fifty‐three percent of all outcomes (*n* = 345) were only reported in one study. Outcomes were grouped into 10 different domains: anthropometric measurements, academic skills, dietary behaviors, fitness and motor skills, parental, physical environment, physiological, psychological, 24‐h movement behaviors, and a miscellaneous outcomes domain. The 10 most frequently reported outcomes covered the anthropometric and dietary behaviors domains. Outcomes completing the 20 most frequently reported outcomes additionally covered the domains of 24‐h movement behaviors, fitness and motor skills, and physiological and psychological domains. File S4 includes a summary description of the most frequently reported outcomes for each outcome domain.

**TABLE 1:** The top 20 outcomes most frequently reported in included studies, along with their reporting frequency, the domain in which they were grouped and the underlying unique outcome.

	Reported outcome	Frequency	Domain	Unique outcome
1	BMI (kg/m^2^)	128	Anthropometric	Body composition
2	BMI‐z	108	Anthropometric	Body composition
3	BMI categories	100	Anthropometric	Body composition
4	Waist circumference	80	Anthropometric	Fat distribution
5	Body fat	67	Anthropometric	Body composition
6	Consumption of sweetened beverages	64	Dietary behaviors	Drink intake
7	Consumption of fruits	59	Dietary behaviors	Food intake (amount, type)
8	Consumption of vegetables	59	Dietary behaviors	Food intake (amount, type)
9	Weight	51	Anthropometric	Body composition
10	Height^a^	50	Anthropometric	Not an outcome
11	Moderate‐to‐vigorous physical activity, time spent	45	24‐h movement behaviors	Physical activity—duration (intensity)
12	Skinfold thickness	41	Anthropometric	Body composition
13	Cholesterol levels	41	Physiological	Lipid metabolism
14	Physical activity, time spent	37	24‐h movement behaviors	Physical activity—duration
15	Aerobic, cardiovascular endurance	37	Fitness and motor skills	Cardiorespiratory fitness
16	Systolic blood pressure	33	Physiological	Blood pressure
17	Diastolic blood pressure	33	Physiological	Blood pressure
18	Sedentary behavior, time spent	32	24‐h movement behaviors	Sedentary behavior—duration
19	BMI percentile	31	Anthropometric	Body composition
20	Knowledge on (healthy) nutrition	26	Psychological	Knowledge (type)

Abbreviation: BMI, body mass index.

^a^ The outcome “height” was reported in 49 of the included studies, yet the experts classified it as “not an outcome” as they discussed that height is not an outcome in itself in these types of school‐based overweight/obesity prevention studies, but measured to calculate anthropometric outcomes. [Correction added on 18 March 2026, after first online publication: The fifth reported outcome has been added in this version.]

### 3.4 Identified Unique Outcomes

Table 2 presents the final list of 68 unique outcomes that the expert panel identified from the 646 reported outcomes, grouped by outcome domain and including their working descriptions and examples of reported outcomes. Some of the reported outcomes were classified as “not an outcome” as, according to the experts, these outcomes reflected an interpretation or an adherence‐related result. For example, for the reported outcome “intake of highly recommended food,” no unique outcome was identified as what is meant by “highly recommended food” was considered to be open to interpretation. Most unique outcomes were identified in the psychological domain (*n* = 16), followed by the 24‐h movement behaviors domain (*n* = 10), the fitness and motor skills domain (*n* = 9), dietary behaviors domains (*n* = 11), the parental domain (*n* = 7), the physiological domain (*n* = 6), the academic skills domain (*n* = 4), the physical environment domain (*n* = 3), and the anthropometric domain (*n* = 2).

**TABLE 2:** Unique outcomes, their working descriptions, and examples of reported outcomes, sorted alphabetically (first by domain, then by unique outcome).

Unique outcome^a^	Working description	Examples of reported outcomes from published studies
**Anthropometric domain—**Including outcomes related to physical properties of the body		
Body composition	General outcome regarding the amount of fat, fat‐free, muscle mass, and water, i.e., the composition of the body	BMI‐z; body fat; bone mass
Fat distribution	Outcome specific regarding location of fat	Waist circumference; fat ratio; trunk fat
**Academic skills domain—**Including outcomes related to skills that help students succeeding in academic settings		
Academic attainment	Outcomes related to grades achieved by students	School grades; academic attainment, math; academic attainment, reading
Academic skills	Outcomes related to skills that students need to succeed in academic settings	Writing skills; oral language skills; mathematics skills
Executive functioning	Outcomes related to the management of cognitive processes	Attention; cognitive performance; Sternberg paradigm
Negative classroom behavior	Outcomes related to undesired behaviors in the classroom	Referrals; classroom behavior
**Dietary behaviors domain—**Including outcomes related to food choice, eating behavior, and dietary intake/nutrition		
Adherence to dietary guidelines (type)	Outcomes related to meeting dietary guidelines, recommending the necessary types and amounts of foods, drinks, and nutrients for children	Adherence to fruit recommendation; recommended amount of fruit and vegetable intake
Energy intake (amount, type)	Outcomes related to the intake of energy; including the amount and type of energy intake	Total energy intake; energy intake from sugar
Carbohydrate intake (amount, timing, type)	Outcomes related to the intake of carbohydrates; including the amount, timing, and type of carbohydrate intake	Intake of (added) sugars in lunch order; plate waste of added sugar; intake of carbohydrates
Fat intake (amount, type)	Outcomes related to the intake of fat; including the amount and type of fat intake	Intake of trans fat; intake of saturated fat; intake of cholesterol
Protein intake (amount, timing)	Outcomes related to the intake of fat; including the amount and type of fat intake	Intake of proteins at lunch; total intake of proteins
Micronutrient intake (type)	Outcomes related to the intake of vitamins and minerals; including the type of micronutrient intake	Intake of calcium; intake of vitamins; sodium selection
Food intake (amount, context, frequency, preparation, timing, type)	Outcomes related to the intake of foods; including the amount, context, frequency, preparation, timing, and type of food intake	Consumption of baked goods at breakfast; frequency of consuming fast‐food; consumption of pastry at home
Drink intake (amount, context, frequency, timing, type)	Outcomes related to the intake of drinks; including the amount, context, frequency, timing, and type of drink intake	Consumption of water in class; consumption of dairy products at breakfast; frequency of drinks intake
Portion size (type)	Outcomes related to the portion size of the consumed foods/drinks; including the type of foods/drinks	Size of fruit; portion size of fast‐food; size of sports drinks
Food preference (type)	Outcomes related to having preference for certain foods over others; including the type of foods	Food selection; selection of fruit; plate waste carbohydrates
Drink preference (type)	Outcomes related to having preference for certain drinks over others; including the type of drinks	Selection of white milk
**Fitness and motor skills domains—**Including outcomes related to the condition of being physically fit and having skills to perform body movements		
Cardiorespiratory fitness	Outcomes related to the capacity of the circulatory and respiratory systems to supply oxygen to skeletal muscles needed for physical activity	VO_2_ consumption; maximum heart rate; aerobic fitness
Fundamental movement skills	Outcomes including basic learned movement patterns that do not occur naturally and are considered foundational for complex motor skills and physical activities	Coordination; obstacle jumping; motor development
Agility	Outcomes related to the ability to rapidly change body direction, accelerate or decelerate	Agility
Balance	Outcomes related to the ability to maintain the body's center of gravity over its base of support	Balance
Flexibility	Outcomes related to the ability of a joint or series of joints to move through an unrestricted, pain free range of motion	Flexibility
Speed	Outcomes related to the ability of your whole body, arm, or leg to move from one point to another as quickly as possible	Speed; submax speed; peak speed
Muscle endurance	Outcomes related to the ability of muscles to exert force (against resistance) over a sustained period of time	Abdominal strength (sit ups); bent arm hang; push ups
Muscle power	Outcomes related to the ability to exert a maximal force in as short a time as possible	Vertical jump; explosive strength; standing long jump
Muscle strength	Outcomes related to the amount of force a muscle can produce with a single maximal effort	Muscular strength; handgrip strength; back extensor strength
**Parental domain—**Including outcomes related to parental characteristics, skills, and behaviors		
Parental behavior^b^	Outcomes related to behaviors performed by parents	Parents' physical activity
Parental body composition	Outcomes related to the body composition of parents	BMI parents; BMI category parents
Parent intentions^b^	Outcomes related to a parent's prior conscious decision to perform a behavior	Parental intention to change eating habits
Parent outcome expectations^b^	Outcomes related to the beliefs or judgments of parents about their child's future, or intended, achievements/behaviors	Parent outcome expectation to bike to school
Parent practices^b^	Outcomes related to the totality of attitudes, values, beliefs, and behaviors that parents bring to settings in which they interact with their child or children	Dietary related parenting practices
Parenting style	Outcomes related to the combination of attitudes, practices, and emotional climate that parents create when interacting with their child	Authoritative parenting
Parental support^b^	Outcomes related to the provision of assistance or comfort by parents to their children, typically to help them cope with biological, psychological, and social stressors	Parents' social support to improve children's consumption of fruit and vegetables; parent–child self‐efficacy to bike to school
**Physical and political environment domain—**Including outcomes related to the physical environment		
Accessibility (type, context)	Outcomes related to the quality of being accessible, e.g., to reach or obtain; including the type and context	Easily accessible water source in class; quality of independent access to play environment; accessibility of fruit and vegetables at home; food insecurity
Availability (type, context)	Outcomes related to the quality of being available, e.g., to buy, use, or reach; including the type and context	Availability of sports venues; TV in bedroom; availability of soda
Policy (type)	Outcomes related to a set of ideas or plans used as a basis for making decisions; including the type of policy	Physical activity school policy
**Physiological domain—**Including outcomes related to the functioning of the body		
Blood pressure	Outcomes related to within the major arterial system of the body	Systolic blood pressure; diastolic blood pressure; mean arterial pressure
Glucose metabolism	Outcomes related to the process by which a simple sugar found in many foods is processed and used to produce energy	Glucose; insulin levels; HbA1C levels
Lipid metabolism	Outcomes related to the process of synthesizing, breaking down, and storing fat in cells for energy storage	Triglycerides; apolipoprotein B; cholesterol levels
Micronutrient status	Outcomes related to the vitamins and minerals needed by the body in very small amounts	Iron levels; ferritin levels
Immunity	Outcomes related to the human body's inherent mechanism to combat attacks from foreign invaders	Transmembrane proteins; migratory capacity
Inflammation	Outcomes related to the body's biological response to harmful stimuli, such as irritants, pathogens, and damaged cells	C‐reactive protein
**Psychological domain—**Including outcomes related to the mental and emotional state of a person *Each unique outcome in this domain needs further specification (i.e., what is the “type” of, for example, attitude) to measure any factor related to EBRBs*.		
Attitude	Outcomes reflecting a relatively enduring and general evaluation of an object, person, group, issue, or concept on a dimension ranging from negative to positive. Attitudes provide summary evaluations of target objects and are often assumed to be derived from specific beliefs, emotions, and past behaviors associated with those objects	Attitudes towards sports; attitude towards obesity preventive behavior; attitude towards fruit
Awareness	Outcomes related to the perception or knowledge of something; accurate reportability of something perceived or known is widely used as a behavioral index of conscious awareness	Awareness of food intake to gain excess body weight; awareness of physical activity and TV time to gain excess body weight
Behavioral outcomes	Outcomes that are observable, intervention‐targeted self‐regulatory actions for monitoring, planning, or controlling health behaviors, specified using A(A)CT (Actor, Action, Context, Time) and reported as frequency, duration, or quantity.	Writing/adjusting goals; self‐regulation for healthy eating; nutrition related skills, such as reading nutrition labels
Habit	Outcomes a well‐learned behavior or automatic sequence of behaviors that is relatively situation specific and over time has become motorically reflexive and independent of motivational or cognitive influence—that is, it is performed with little or no conscious intent.	Family physical activity habits; sports habits; habit strength towards fruit and vegetable consumption
Intention	Outcomes related to a prior conscious decision to perform a behavior	Food choice intention; behavior intention towards physical activity; intention towards obesity preventive behavior
Knowledge	Outcomes related the state of being familiar with something or aware of its existence, usually resulting from experience or study	Knowledge of vegetables; media knowledge; knowledge of physical activity and diet
Motivation	Outcomes related to the impetus that gives purpose or direction to behavior and operates in humans at a conscious or unconscious levels	Motivation for healthy eating; motivation for physical activity; self‐determined motivation
Outcome expectation	Outcomes related to an individual's beliefs that are associated with future, or intended, behaviors and that are believed to either promote or inhibit these behaviors	Outcome expectation towards physical activity; outcome expectation towards tv viewing; outcome expectation towards fast food consumption
Preference	Outcomes related to the probability of occurrence of one of two or more concurrently available responses, usually expressed as either a relative frequency or a ratio.	Food preference; preference for vegetables; preference of body image
Health‐related quality of life	Outcomes related to the perceived impact of a child's health on physical, emotional, social, and role/school functioning	Physical quality of life; psychological quality of life; social quality of life
Satisfaction/enjoyment	Outcomes related to the perception of great pleasure and happiness brought on by success in or simple satisfaction with an activity	Satisfaction with activity choices; physical activity enjoyment; satisfaction with family food choices
Self‐efficacy	Outcomes related to an individual's subjective perception of their capability to perform in a given setting or to attain desired results	Self‐efficacy to eat fruits/vegetables; self‐efficacy to be physically active; self‐efficacy towards sweetened beverage consumption
Self‐concept	Outcomes related to a description and evaluation of oneself, including psychological and physical characteristics, qualities, skills, roles, and so forth.	Perceived body image/physical appearance; perceived physical competence; physical self‐worth
Self‐esteem	Outcomes related to the degree to which the qualities and characteristics contained in one's self‐concept are perceived to be positive	Self‐esteem
Social support	Outcomes related to the provision of assistance or comfort to others, typically to help them cope with biological, psychological, and social stressors	Perceived social support; physical activity together; teacher participation in classroom physical activity
Well‐being	Outcomes capturing subjective well‐being—positive affect and life satisfaction alongside low psychological distress—reflecting emotional and social functioning; distinct from health‐related quality of life	Having a good feeling; well‐being index score; mental well‐being
**24‐h movement behaviors domain**—Including outcomes related to physical activity,^c^ sedentary behavior,^d^ and sleep		
Energy expenditure	Outcomes related to the amount of energy an individual uses to maintain essential body functions (respiration, circulation, digestion) and as a result of physical activity.	Energy expenditure; Intensity of physical activity in kcal/kg/h
Adherence to physical activity guidelines	Outcomes related to meeting physical activity recommendations, recommending that children accumulate at least 60 min of moderate‐to‐vigorous physical activity	Meeting physical activity guidelines
Physical activity—Type (duration, context, frequency, intensity, timing)	Outcomes related to the type of physical activity, including aerobic, muscle strengthening, bone‐strengthening, balance, and flexibility activities; including the duration, frequency, intensity, and context of the type of physical activity	Time spent in open air games; exercise for at least 30 min after school; frequency of playing outdoors
Physical activity—Duration (intensity)	Outcomes related to the amount of time spent in physical activity; including the intensity of the physical activity	Time spent in light physical activity; time spent in moderate‐to‐vigorous physical activity; time spent in vigorous physical activity
Physical activity—Frequency (intensity)	Outcomes related to the number of times participating in physical activity; including the intensity of the physical activity	Frequency of physical activity; frequency of participating in > 60 min of moderate‐to‐vigorous physical activity
Physical activity—Volume	Outcomes related to the sum of all activity accumulated during a given timeframe	Step counts
Sedentary behavior—Type (duration, frequency, timing)	Outcomes related to the type of sedentary behavior; including the duration, frequency, and timing of the sedentary behaviors	Time spent watching TV on weekend days; frequency of screen use; sitting time
Sedentary behavior—Duration (timing)	Outcomes related to the amount of time spent in sedentary behavior; including the timing of the sedentary behaviors	Time spent in sedentary activities; time spent in sedentary activities on weekend days; time spent in sedentary behaviors on an average day
Sedentary behavior—Frequency (timing)	Outcomes related to the number of times participating in sedentary behavior; including the timing of the sedentary behaviors	Frequency of sedentary breaks; number of sedentary breaks on weekdays
Sleep (duration)	Outcomes related to a naturally recurring and reversible biobehavioral state characterized by relative immobility, perceptual disengagement, and subdued consciousness; including the duration of sleep	Sleep time; sleep duration; sleeping 10 h or more

Abbreviations: EBRBs, energy balance related behaviors; TV, television.

^a^ The details between brackets refer to the specific aspects of the unique outcome, as reflected by the reported outcomes. For example, the unique outcome physical activity—duration (intensity) specifies that reported outcomes on the duration of physical activity include the intensity of the activity (e.g., moderate physical activity).

^b^ These unique outcomes in this domain need further specification (i.e., what is the “type” of, for example, parental support) to measure any factor related to EBRBs.

^c^ Physical activity refers to any bodily movement produced by skeletal muscles that results in energy expenditure [26].

^d^ Sedentary behavior refers to activities that do not increase energy expenditure substantially above the resting level and includes activities such as sleeping, sitting, lying down, and watching television, and other forms of screen‐based entertainment [27].

Several outcomes in the dietary behaviors, parental, physical and political environment, psychological, and 24‐h movement behavior domains need further specification, as indicated between brackets in Table 2. For example, the outcome drink intake in the dietary behaviors domain needs specification of the “amount” (e.g., servings/day), “context” (e.g., in class), “frequency” (times/week), “timing” (e.g., at breakfast), and “type” (e.g., soft drink). The outcome physical activity duration in the 24‐h movement behaviors domain needs specification of among others the “intensity” (e.g., light physical activity).

## 4 Discussion

Our scoping review on outcomes reported in studies evaluating school‐based interventions targeting overweight and obesity in 6‐ to 12‐year‐old children showed that the 262 included studies reported 646 different outcomes. This finding confirms the heterogeneity in reported outcomes and substantiates the need for a COS to enhance the comparability, relevance, and reliability of future research [16, 17, 20]. BMI (kg/m^2^) was the most frequently reported outcome, being reported in only 49% of studies (*n* = 128). Consequently, when considering a meta‐analysis to assess the effectiveness of school‐based interventions aimed at preventing overweight and obesity in childhood on BMI (kg/m^2^), only 128 out of 262 studies would be suitable for this meta‐analysis. If we consider all reported outcomes and, for example, two to five studies as the minimum number of studies needed for conducting a meta‐analysis, 54% to 85% of reported outcomes could not be included in a meta‐analysis. The expert panel members identified 68 unique outcomes underlying the reported outcomes, grouped in 10 outcome domains, which will be taken forward and presented in an international eDelphi study aimed at obtaining consensus on which of these outcomes should be included in the final COS.

A previous scoping review summarizing outcomes reported in randomized controlled trials of early childhood (children aged from birth to 5 years) obesity prevention interventions identified 221 unique outcomes across 18 outcome domains [28]. Our review identified fewer unique outcomes (i.e., *n* = 68) compared to the review of Brown et al. [28], which indicates a larger variance in outcomes used in obesity prevention studies in the early years, compared to school‐based obesity prevention studies in 6‐ to 12‐year‐old children. Alternatively, the difference in number of unique outcomes could be explained by a difference in the process of identifying unique outcomes. Brown et al. [28] describe an iterative process of categorizing, sorting, and merging of outcomes, conducted by one researcher and checked by a member of the steering group. In our current project, we assigned an expert panel the task of identifying unique outcomes underlying the reported outcomes irrespective of how outcomes were defined and measured. Despite this difference, similarities in the broad range of outcome domains and outcomes within these domains are evident: the anthropological, physiological, parental, dietary behaviors, fitness and motor skills, and physical and political environmental domains (and outcomes in these domains) described in our review have considerable overlap with the domains anthropometry, dietary intake, physical activity, sedentary behavior, sleep, parent/caregivers practices, motor skill development, environmental, and blood and lymphatic system (and outcomes in these domains) as described in the review of Brown et al. [17]. Remarkably, outcomes described in the economic and oral health domains in the review of Brown et al. [28] were not identified in the present review.

As described in our protocol paper [22], we initially planned to group the identified unique outcomes by outcome domain according to the taxonomy by Dodd et al. [25]. However, this taxonomy includes clinical outcome domains, which we felt did not fit with the unique outcomes identified in the included studies aimed at the prevention of overweight and obesity. Rather, we created additional new domains by which we grouped the identified unique outcomes, largely overlapping with the domains as described by Brown et al. [28]. To allow future COS initiatives in the field of prevention, we suggest extending the outcome domain taxonomy of Dodd et al. [25] with domains covering outcomes relevant for prevention. The domains suggested in the present study and the study of Brown et al. [28] could inform outcome domains used in prevention or public health.

Unsurprisingly, and in line with the findings of Brown et al. [28], the most frequently reported outcomes were grouped in the anthropometric domain, followed by outcomes in the dietary behavior domain and the 24‐h movement behaviors domain, reflecting the energy balance‐related behaviors that, in case of a positive balance, cause excessive weight gain. Outcomes in these domains provide important insights into whether the intervention has been effective in terms of changes in targeted behaviors, whereas outcomes in the other domains provide insight into potential determinants of the targeted behaviors [18].

We will present the final list of unique outcomes to relevant stakeholders in an international eDelphi study to obtain consensus on which of these outcomes should be included in the final COS for school‐based intervention studies aimed at preventing obesity in 6‐ to 12‐year‐old children. It will be interesting to find out whether there is consensus on including outcomes from all the described domains. Additionally, as the anthropometric, dietary behavior, and 24‐h movement behavior domains include 21 unique outcomes in total, it will be interesting to find out for which of these unique outcomes consensus will be achieved. Through an international eDelphi study and consensus meeting to establish a COS for early childhood obesity prevention interventions, Brown et al. [29] showed that consensus was reached for 22 outcomes from nine outcome domains (out of the 221 outcomes across 18 domains). This final COS includes outcomes reflecting targeted behaviors and potential determinants of the targeted behaviors, covering the anthropometry, dietary intake, sedentary behavior, physical activity, sleep, outcomes in parents/caregivers, environmental, emotional/cognitive functioning, and economics domains. Notably, a COS prescribes a minimal set of outcomes to be measured and reported. Any country or context specific outcome preferences that are not considered “core” in the established COS could be added in a country or context specific extension of the COS, that should be established through a separate consensus process.

To identify outcomes relevant to the children themselves, we conducted standardized interactive focus groups in six countries across three continents, involving 159 children aged 8–13 years [30]. Children proposed 170 unique outcomes, with most top‐20 outcomes reflecting mental, social, or cognitive aspects of health and well‐being (e.g., “having fun,” “being happy,” and “making friends”), and none of the top‐20 outcomes relating to children's body weight and fat mass [30]. In contrast, the present scoping review showed that the top‐20 of most reported outcomes include a large number of outcomes related to children's body weight or fat mass. All outcomes that were reported by children in at least two countries and were overall rated by children as “very important” will be added to the list of unique outcomes that will be put forward and presented in the eDelphi study (including “being healthy,” “healthy diet,” “concentration,” “having fun,” and “feeling happy”).

Once consensus is reached on *what* to measure, it is essential to identify and obtain consensus on the most appropriate measurement instruments for assessing the core outcomes in the COS (i.e., *how* to measure) [21, 31]. Hereby, not only the optimal validity but also the feasibility of the measurement instruments should be considered, which calls for reviews on measurement properties (e.g., validity, reliability, and feasibility) of different measurement instruments [31]. We found a large variety of reported outcomes belonging to a unique outcome, for example, only two unique outcomes were identified from the 51 reported outcomes in the anthropometric domain. This finding may point to future challenges in obtaining consensus for the most appropriate measurement instrument for the core outcomes in the future COS.

The COS we are currently developing would be of great value in guiding researchers in choosing the outcomes of future intervention studies and reducing research waste by including outcomes relevant to all key stakeholders. However, the uptake of a COS is variable and typically low [32], indicating that developing a COS is not enough. Barriers and facilitators to COS uptake are being identified [33] and our role is to prioritize the involvement of relevant stakeholders during the eDelphi study as well as contacting journal editorial teams and funders that publish and fund school‐based obesity prevention studies. It would also be interesting to chart the development time course and uptake of COS that were developed without funding or with partial/sporadic funding like ours.

A strength of this study is the thorough identification of reported outcomes in a scoping review by two reviewers independently. The assessment of unique outcomes underlying all reported outcomes by a group of international experts during multiple meetings and feedback rounds additionally strengthens this study. The study has several limitations; however, given the breadth and number of studies included in this review, do not significantly impact the strength of the study results. In case of lack of clarity on outcomes, we did not contact the corresponding author of the study to obtain clarification. By merging outcomes that we believed were similar yet differed in wording or reflected a specific timing or context (e.g., “time spent in physical activity” and “time spent in weekend day physical activity”), important aspects of an outcome (in this case, the timing of physical activity) might have got lost. To prevent this, we added specific aspects of the unique outcomes between brackets (e.g., physical activity duration [timing]). Moreover, we will encourage the participants in our eDelphi study (including researchers who contributed to the studies in the current scoping review) to add (specific aspects of) outcomes they feel are currently missing in our list of unique outcomes. We may also have missed outcomes due to publication bias and reporting bias, where authors publish only outcomes with favorable results as well as due to the exclusion of studies published in languages other than English. Research from the global south is underrepresented in the included studies, yet based on the number of unique outcomes we anticipate that this review covers all unique outcomes related to school‐based obesity prevention interventions for 6‐ to 12‐year‐olds. Finally, our decision to exclude studies focusing on samples with a disease or disorder and studies evaluating obesity treatment interventions and to focus on the school setting means that the future COS will also have this specific focus. We recommend future studies to set up a Delphi study to explore the relevance of the outcomes in our COS beyond school‐based obesity prevention interventions for 6‐ to 12‐year‐olds.

We conclude that the heterogeneity in outcomes that are currently reported in studies evaluating school‐based interventions targeting childhood overweight and obesity confirms the need for consensus on the most important outcomes in a COS for this public health problem. The identified 68 unique outcomes serve as a starting point in the international online Delphi study to establish a core outcome set for school‐based interventions targeting childhood overweight and obesity.

## Conflicts of Interest

The authors declare no conflicts of interest.

## Supporting information


**Data S1:** Supporting information.

## Data Availability

The data that support the findings of this study are available in the Supporting Information of this article.
