# Supplementary material for: Evaluating School‐Based Obesity Prevention Interventions in 6‐ to 12‐Year‐Old Children: A Scoping Review of All Reported Outcomes and Expert Consultation
Source: Obes Rev. 2026 Mar 12;27(10):e70125. doi: 10.1111/obr.70125 (PMC13551394; doi:10.1111/obr.70125)
Supplement: Supplementary file 1 [file OBR-27-e70125-s001.pdf]

Evaluating school-based obesity prevention interventions in 6-12-year-old children: a scoping review of all reported outcomes and expert consultation

Teatske M. Altenburg: Amsterdam UMC, location Vrije Universiteit Amsterdam, De Boelelaan 1117, Amsterdam, the Netherlands; t.altenburg@amsterdamumc.nl

Lotte W. de Vries: Amsterdam UMC, location Vrije Universiteit Amsterdam, De Boelelaan 1117, Amsterdam, the Netherlands; lotte.w.devries@gmail.com

Iris J. Grooten: Amsterdam UMC, location University of Amsterdam, Meibergdreef 9 Amsterdam, the Netherlands; i.j.grooten@amsterdamumc.nl

Janneke van 't Hooft: Amsterdam UMC, location University of Amsterdam, Meibergdreef 9 Amsterdam, the Netherlands; j.vanthooft1@amsterdamumc.nl

Arend W. van Deutekom: Erasmus MC-Sophia Children's Hospital, Dr. Molewaterplein 40, Rotterdam, the Netherlands; a.vandeutekom@erasmusmc.nl

Tessa Roseboom: Amsterdam UMC, location University of Amsterdam, Meibergdreef 9 Amsterdam, the Netherlands; t.j.roseboom@amsterdamumc.nl

Erica van den Akker: Erasmus MC-Sophia Children's Hospital, Dr. Molewaterplein 40, Rotterdam, the Netherlands; e.l.t.vandenakker@erasmusmc.nl

Michael Duncan: Coventry University, Coventry, UK
aa8396@coventry.ac.uk

Ulf Ekelund: Norwegian School of Sport Sciences, Sognsveien 220, Oslo, Norway; ulfek@nih.no

John J Reilly: University of Strathclyde, Glasgow G1 1XQ, United Kingdom; john.j.reilly@strath.ac.uk

Lisa Reilly: NHS Tayside Nutrition & Dietetics, Dundee DD4 9FF, United Kindgdom; lisa.reilly3@nhs.scot

Annette Stafleu: The Netherlands Nutrition Centre, Bezuidenhoutseweg 105, The Hague, The Netherlands; stafleu@voedingscentrum.nl

Martyn Standage: University of Bath, Bath BA2 7AY, United Kingdom; spsms@bath.ac.uk

Mai J.M Chinapaw: Amsterdam UMC, location Vrije Universiteit Amsterdam, De Boelelaan 1117, Amsterdam, the Netherlands m.chinapaw@amsterdamumc.nl

Deirdre M. Harrington: University of Strathclyde, Glasgow G1 1XQ, United Kingdom; deirdre.harrington@strath.ac.uk

Supplementary File 1: Search strategy in PubMed

Search	Query
**#10**	#8 AND #9
**#9**	"Child"[Mesh] OR child*[tiab] OR schoolchild*[tiab] OR pediatri*[tiab] OR paediatr*[tiab] OR "youth"[tiab] OR "youths"[tiab]
**#8**	#1 AND #4 AND #7
**#7**	#5 AND #6
**#6**	("population level"[tiab] OR "population based"[tiab] OR "population orientated"[tiab] OR "population oriented"[tiab] OR "community level"[tiab] OR "community based"[tiab] OR "community orientated"[tiab] OR "community oriented"[tiab] OR school*[tiab] OR "school-based"[tiab] OR education*[tiab] OR institution[tiab] OR institutional[tiab] OR class*[tiab]) AND (intervention*[tiab] OR prevent*[tiab] OR policy[tiab] OR policies[tiab] OR program*[tiab] OR project*[tiab] OR change[tiab] OR changing[tiab] OR trial*[tiab] OR approach*[tiab])
**#5**	"Clinical Trial" [Publication Type] OR "Clinical Trials as Topic"[Mesh] OR "Randomized Controlled Trial" [Publication Type] OR "Randomized Controlled Trials as Topic"[Mesh] OR "Random Allocation"[Mesh] OR "Double-Blind Method"[Mesh] OR "Single-Blind Method"[Mesh] OR "randomized controlled trial"[tiab] OR "randomised controlled trial"[tiab] OR RCT[tiab] OR "controlled trial*"[tiab] OR "clinical trial*"[tiab] OR trial*[tiab] OR non-randomized[tiab] OR non-randomised[tiab] OR (control*[tiab] AND (experiment*[tiab] OR evaluat*[tiab] OR effective*[tiab] OR intervention*[tiab])) OR random*[tiab]
**#4**	#2 OR #3
**#3**	"health promotion"[Mesh] OR "health promotion"[tiab] OR "health behavior"[tiab] OR "health behaviour"[tiab] OR (policy[tiab] AND (social[tiab] OR school*[tiab] OR food[tiab] OR public[tiab] OR urban[tiab] OR environmental[tiab] OR fiscal[tiab])) OR "urban planning"[tiab] OR "city planning"[tiab] OR "built environment"[tiab] OR "social environment"[tiab] OR "physical environment"[tiab] OR "cultural environment"[tiab] OR "urban environment"[tiab] OR "school environment"[tiab] OR neighbourhood[tiab] OR community[tiab] OR societal[tiab] OR "social interventions"[tiab] OR "community interventions"[tiab] OR "obesogenic environment"[tiab] OR "individual level"[tiab] OR lifestyle[tiab] OR individual[tiab] OR tax*[tiab] OR subsid*[tiab] OR "health education"[tiab] OR "social marketing"[tiab] OR (diet[tiab] AND (advice[tiab] OR counselling[tiab])) OR (exercise[tiab] AND (advice[tiab] OR counselling[tiab])) OR "weight management"[tiab] OR "cash transfer*"[tiab] OR "lifestyle counselling"[tiab] OR "behavioural counselling"[tiab] OR "behavioral counselling"[tiab] OR "exercise on prescription"[tiab] OR exercise[tiab] OR "health trainer*"[tiab] OR school*[tiab] OR workplace[tiab] OR campaign*[tiab] OR "access to facilities"[tiab] OR "green space"[tiab] OR "walking ability"[tiab] OR "food label*"[tiab] OR "food advert*"[tiab]
**#2**	"Health Promotion"[Mesh:NoExp]
**#1**	"Body Weights and Measures"[Mesh:NoExp] OR "Body Weight"[Mesh] OR "Obesity"[Mesh] OR "Overweight"[Mesh] OR "Adiposity"[Mesh] OR obes*[tiab] OR overweight[tiab]

Search strategy in Embase

Search	Query
**#11**	#10 AND ([child]/lim OR [school]/lim) AND [humans]/lim
**#10**	#8 AND #9
**#9**	"Child"/exp OR child*:ab,ti OR schoolchild*:ab,ti OR pediatri*:ab,ti OR paediatr*:ab,ti OR “youth”:ab,ti OR “youths”:ab,ti
**#8**	#1 AND #4 AND #7
**#7**	#5 AND #6
**#6**	(“population level”:ab,ti OR “population based”:ab,ti OR “population orientated”:ab,ti OR “population oriented”:ab,ti OR “community level”:ab,ti OR “community based”:ab,ti OR “community orientated”:ab,ti OR “community oriented”:ab,ti OR school*:ab,ti OR “school-based”:ab,ti OR education*:ab,ti OR institution:ab,ti OR institutional:ab,ti OR class*:ab,ti) AND (intervention*:ab,ti OR prevent*:ab,ti OR policy:ab,ti OR policies:ab,ti OR program*:ab,ti OR project*:ab,ti OR change:ab,ti OR changing:ab,ti OR trial*:ab,ti OR approach*:ab,ti)
**#5**	"Clinical Trial"/exp OR "Randomized Controlled Trial"/exp OR “Controlled Clinical Trial”/exp OR "Random Allocation"/exp OR "Double-Blind Method"/exp OR "Single-Blind Method"/exp OR “clinical trial*”:ab,ti OR trial*:ab,ti OR “randomized controlled trial*”:ab,ti OR “randomised controlled trial*”:ab,ti OR “controlled trial*”:ab,ti OR RCT:ab,ti OR random*:ab,ti OR non-randomized:ab,ti OR non-randomised:ab,ti OR (control*:ab,ti AND (experiment*:ab,ti OR evaluat*:ab,ti OR effective*:ab,ti OR intervention*:ab,ti))
**#4**	#2 OR #3
**#3**	“health promotion”/exp OR “health promotion”:ab,ti OR “health behavior”:ab,ti OR “health behaviour”:ab,ti OR (policy:ab,ti AND (social:ab,ti OR school*:ab,ti OR food:ab,ti OR public:ab,ti OR urban:ab,ti OR environmental:ab,ti OR fiscal:ab,ti)) OR “urban planning”:ab,ti OR “city planning”:ab,ti OR “built environment”:ab,ti OR “social environment”:ab,ti OR “physical environment”:ab,ti OR “cultural environment”:ab,ti OR “urban environment”:ab,ti OR “school environment”:ab,ti OR neighbourhood:ab,ti OR community:ab,ti OR societal:ab,ti OR “social interventions”:ab,ti OR ”community interventions”:ab,ti OR “obesogenic environment”:ab,ti OR “individual level”:ab,ti OR lifestyle:ab,ti OR individual:ab,ti OR tax*:ab,ti OR subsid*:ab,ti OR “health education”:ab,ti OR “social marketing”:ab,ti OR (diet:ab,ti AND (advice:ab,ti OR counselling:ab,ti)) OR (exercise:ab,ti AND (advice:ab,ti OR counselling:ab,ti)) OR “weight management”:ab,ti OR “cash transfer*”:ab,ti OR “lifestyle counselling”:ab,ti OR “behavioural counselling”:ab,ti OR “behavioral counselling”:ab,ti OR “exercise on prescription”:ab,ti OR exercise:ab,ti OR “health trainer*”:ab,ti OR school*:ab,ti OR workplace:ab,ti OR campaign*:ab,ti OR “access to facilities”:ab,ti OR “green space”:ab,ti OR “walking ability”:ab,ti OR “food label*”:ab,ti OR “food advert*”:ab,ti
**#2**	"Health Promotion"/de
**#1**	"Body Weights and Measures"/de OR "Body Weight"/exp OR "Obesity"/exp OR “Overweight”/exp OR “Adiposity”/exp OR obes*:ab,ti OR overweight:ab,ti

Search strategy in PsycInfo

Search	Query
**#11**	#8 AND #9 Narrow by SubjectAge: - school age (6-12 yrs)<br><br>10 SU AGE (6-12 yrs)
**#10**	#8 AND #9
**#9**	In title or abstract: child* OR schoolchild* OR pediatri* OR paediatr* OR “youth” OR “youths”
**#8**	#1 AND #4 AND #7
**#7**	#5 AND #6
**#6**	In title or abstract: (“population level” OR “population based” OR “population orientated” OR “population oriented” OR “community level” OR “community based” OR “community orientated” OR “community oriented” OR school* OR “school-based” OR education* OR institution OR institutional OR class*) AND (intervention* OR prevent* OR policy OR policies OR program* OR project* OR change OR changing OR trial* OR approach*)
**#5**	In Mesh Subject Heading: "Clinical Trial*" OR "Randomized Controlled Trial*" OR “Controlled Clinical Trial* OR "Random Allocation" OR "Double-Blind Method" OR "Single-Blind Method" OR<br>In title or abstract:<br>“clinical trial*” OR trial* OR “randomized controlled trial” OR “randomised controlled trial” OR “controlled trial*” OR RCT OR random* OR non-randomized OR non-randomised OR nonrandomized OR nonrandomized OR (control* AND (experiment* OR evaluat* OR effective* OR intervention*))
**#4**	#2 OR #3
**#3**	In title or abstract: “health promotion” OR “health promotion” OR “health behavior” OR “health behaviour” OR (policy AND (social OR school* OR food OR public OR urban OR environmental OR fiscal)) OR “urban planning” OR “city planning” OR “built environment” OR “social environment” OR “physical environment” OR “cultural environment” OR “urban environment” OR “school environment” OR neighbourhood OR community OR societal OR “social interventions” OR ”community interventions” OR “obesogenic environment” OR “individual level” OR lifestyle OR individual OR tax* OR subsid* OR “health education” OR “social marketing” OR (diet AND (advice OR counselling)) OR (exercise AND (advice OR counselling)) OR “weight management” OR “cash transfer*” OR “lifestyle counselling” OR “behavioural counselling” OR “behavioral counselling” OR “exercise on prescription” OR exercise OR “health trainer*” OR school* OR workplace OR campaign* OR “access to facilities” OR “green space” OR “walking ability” OR “food label*” OR “food advert*”
**#2**	MM "Health Promotion"
**#1**	In title or abstract: "Body Weight" OR "Obesity" OR “Overweight” OR “Adiposity” OR obes* OR overweight

Search strategy in Cochrane

Search	Query
**#11**	Restriction: in trials only
**#10**	#8 AND #9
**#9**	[mh child] OR child* OR schoolchild* OR pediatri* OR paediatr* OR youth*
**#8**	#1 AND #4 AND #7
**#7**	#5 AND #6 DEZE DOET HET NIET ?!
**#6**	(“population level” OR “population based” OR “population orientated” OR “population oriented” OR “community level” OR “community based” OR “community orientated” OR “community oriented” OR school* OR “school-based” OR education* OR institution OR institutional OR class*) AND (intervention* OR prevent* OR policy OR policies OR program* OR project* OR change OR changing OR trial* OR approach*)
**#5**	[mh “Clinical Trial”] OR [mh “Randomized Controlled Trial”] OR [mh “Controlled Clinical Trial”] OR [mh “Random Allocation”] OR [mh “Double-Blind Method”] OR [mh “Single-Blind Method”] OR “clinical trial*” OR trial* OR “randomized controlled trial” OR “randomised controlled trial” OR “controlled trial*” OR RCT OR random* OR non-randomized OR non-randomised OR nonrandomized OR nonrandomized OR (control* AND (experiment* OR evaluat* OR effective* OR intervention*))
**#4**	#2 OR #3
**#3**	[mh health promotion] OR “health behavior” OR “health behaviour” OR (policy AND (social OR school* OR food OR public OR urban OR environmental OR fiscal)) OR “urban planning” OR “city planning” OR “built environment” OR “social environment” OR “physical environment” OR “cultural environment” OR “urban environment” OR “school environment” OR neighbourhood OR community OR societal OR “social interventions” OR ”community interventions” OR “obesogenic environment” OR “individual level” OR lifestyle OR individual OR tax* OR subsid* OR “health education” OR “social marketing” OR (diet AND (advice OR counselling)) OR (exercise AND (advice OR counselling)) OR “weight management” OR “cash transfer*” OR “lifestyle counselling” OR “behavioural counselling” OR “behavioral counselling” OR “exercise on prescription” OR exercise OR “health trainer*” OR school* OR workplace OR campaign* OR “access to facilities” OR “green space” OR “walking ability” OR “food label*” OR “food advert*”
**#2**	[mh^Health Promotion]
**#1**	[mh “Body Weight”] OR [mh Obesity] OR [mh Overweight] OR [mh Adiposity] OR obes* OR overweight

Author	Year	Journal	Country	Study Design	Intervention focus	Pop int N	Pop int age	Pop int age SD	Girls (%)	pop ctrl N	pop ctrl age	pop ctrl age SD	Girls (%)	Intervention duration	Timepoints	Outcome(s) reported	Measurement tool
Adab	2014	BMJ Open	UK	Feasibility	Nutrition	269	6,5	0,6	47	305	6,4	0,6	50	1 year	Bl, 1 year, 2 year	BMI z-score	Objectively measured, and calculated using the UK 1990 growth reference charts
				Controlled trial	Physical activity											Waist circumference	Objectively measured
																Skinfold thickness, biceps	Objectively measured
																Skinfold thickness, triceps	Objectively measured
																Skinfold thickness, subscapular	Objectively measured
																Skinfold thickness, suprailiac	Objectively measured
																Skinfold thickness, thigh	Objectively measured
																Percentage body fat	Bioimpedance monitor
																Weight status %	Objectively measured and
																Quality of Life	PedsQL, proxy-report by
																Self-concept	Marsh self-description
																Perceived physical competence	Harter Pictorial Scale for
																Body image perception	Adapted Collin's Pictorial
																24h dietary intake	Child And Dietary Evaluation Tool (CADET), proxy report at school by researcher, at home by parents
																Total energy intake (kJ)	data from CADET
																Amount of fruit and vegetables co	data from CADET
																Amount of suger consumed	data from CADET
																Family dietary habits	questionnaire for parents
																Physical activity levels	measured with accelerometer, which measured acceleration and heart rate
																Total daily volume of physical activ	Calculated with accelerometry
																Mean duration of daily moderate-t	Calculated with accelerometry
																Proportion of children participatin	Calculated with accelerometry
																Family physical activity habits	self-report questionnaire for
Akdemir	2017	The Kuwait medical jou	Turkey	RCT	Nutrition	674	10,36	2,25		675	10,15	2,27		1 year (5x 1 m)	Bl, 1 year	Height (m) (standing)	measurement tape
					Physical activity											Weight (kg)	Objectively measured
																BMI	Objectively measured and calculated with WHO-2007 Reference z score values of the age-matched
																Proportion of overweight/obese	
																Nutritional status / KIDMED Index	Questionnaire
																Daily no. of meals	Self-administered questionnaire. KIDMED Index (Mediterranean Diet Quality Index for Children and Adolescents)
																Regular daily breakfast	Self-administered questionnaire. KIDMED Index (Mediterranean Diet Quality Index for Children and Adolescents)
																Consumption of take-home pastry	Self-administered questionnaire. KIDMED Index (Mediterranean Diet Quality Index for Children and Adolescents)
																Sweet, candy, chocolate consumpt	Self-administered questionnaire. KIDMED Index (Mediterranean Diet Quality Index for Children and Adolescents)
																Daily consumption of fresh fruits	Self-administered questionnaire. KIDMED Index (Mediterranean Diet Quality Index for Children and Adolescents)
																Daily consumption of fresh vegeta	Self-administered questionnaire. KIDMED Index (Mediterranean Diet Quality Index for Children and Adolescents)
																Eating at fast-food restaurants	Self-administered questionnaire. KIDMED Index (Mediterranean Diet Quality Index for Children and Adolescents)
																Consumption of fried food	Self-administered questionnaire. KIDMED Index (Mediterranean Diet Quality Index for Children and Adolescents)
																Consumption of pastry at home	Self-administered questionnaire. KIDMED Index (Mediterranean Diet Quality Index for Children and Adolescents)
																Walking exercise with family mem	Self-administered questionnaire. KIDMED Index (Mediterranean Diet Quality Index for Children and Adolescents)
																Inactivity during physical training l	Self-administered questionnaire. KIDMED Index (Mediterranean Diet Quality Index for Children and Adolescents)
																Playing computer games/TV watch	Self-administered questionnaire. KIDMED Index (Mediterranean Diet Quality Index for Children and Adolescents)
Alexander	2014	Obesity	USA	Controlled trial	Nutrition	289	6 to 8			272	6 to 8			6 monhts		BMI	Objectively measured
					Physical activity											BMI percentiles	Overweight: 85-95%; obesity: >= 95%, based on National Center for Health Statistics
Allafi	2020	Progress in Nutrition	Kuwait	RCT	Physical activity	FB = 85	9-11 year olds	unknown	Overall:	72	9-11 year olds	unknown	Overall:	5 sessions	5 times: during PE clas:	Step counts (pedometer)	objectively measured
						FB + R = 68			51% girls (not specified for Int and control)				51% girls (not specified for Int and control)				
Almas	2013	Arch Dis Child	Pakistan	Controlled trial	Physical activity	131	9 to 11		100%	146	9 to 11		100%	20 weeks		BMI z-score	Objectively measured, and
				Feasibility												Systolic blood pressure	electronic BP monitor
																Diastolic blood pressure	electronic BP monitor
																Physical activity, amount of time	Questionnaire, proxy-report
Angelopoulos	2009	Eur J Pub Health	Greece	RCT	Nutrition	321	10,3	0,4	57	325	10,3	0,4	54%	14 months		Height (cm) (standing)	objectively measured
					Physical activity											Weight (kg)	objectively measured
																BMI	objectively measured
																BMI z-score	Calculated according to CDC growth charts
																Blood pressure	electronic BP monitor
																Daily consumption of fruit	24h recall, proxy-report by researcher
																Daily consumption of vegetable	24h recall, proxy-report by researcher
																Daily consumption of total fat/oils	24h recall, proxy-report by researcher
																Daily consumption of sweets/beve	24h recall, proxy-report by researcher
																Daily consumption of meat	24h recall, proxy-report by researcher
																Daily consumption of grains	24h recall, proxy-report by researcher
																Daily consumption of dairy produc	24h recall, proxy-report by researcher
																Leisure time moderate-to-	Data extracted from self-report
Arlinghaus	2017	Preventing chronic dis	USA	RCT	Nutrition	94				95				1 year	Bl, 6 month, 12 month	Height (standing)	Objectively measured
					Physical activity											Weight	Objectively measured
																BMI	BMI was calculated from students' weight and height
																BMI percentile	CDC guidelines
																zBMI	CDC guidelines
Ariza	2019	the Journal of Nutritio	Spain	Quasi-experimental	Nutrition	1464	8-9-yr olds at b	unknown	50	1609	8-9-yr olds at ba	unknown	52	1 schoolyear (Oc tober 2	2 timepoints: baseline	BMI z-score	Objectively measured, WHO z-score
					Physical activity											Cumulative incidence rate of obesi	Objectively measured
					other											Food frequency: water	Questionnaire -FFQ
																Food frequency: vegetables	Questionnaire -FFQ
																Food frequency: fruits	Questionnaire -FFQ
																Food frequency: meat	Questionnaire - FFQ
																Food frequency: occasional sweet	Questionnaire -FFQ
																Food frequency: occasional fried p	Questionnaire -FFQ
																Food behavior: proper breakfast	Questionnaire
																Food behavior: dinner in front of t	Questionnaire
																Food behavior: eating at school	Questionnaire
																Food behavior: global nutrition sc	Questionnaire
																Physical activity: practicing out-of-	Questionnaire
																Physical activity: engaging in physc	Questionnaire
																Daily use of screens: on weekdays	Questionnaire
																Daily use of screens: on weekends	Questionnaire
																Daily use of screens: global activity	Questionnaire
																Daily use of screens: global score	Questionnaire
																Height	Objectively measured, cm
																Weight	Objectively measured, kg
Bacardí-Gascon	2012	Nutr Hosp	Mexico	RCT	Nutrition	280	8,5	0,7	48	252	8,4	0,7	50	6 months	18 month fu	BMI	objectively measured
				Quasi-experimental	Physical activity											BMI z-score	classified according to WHO charts
																Waist circumference	objectively measured
																Weight status %	calculated with BMI z-score
																Overweight/obesity incidence	From normal weight to
																Overweight/obesity remission	From overweight/obesity to
																Abdominal obesity	90th percentile of WC

																Fruits, number of portions	self report questionnaire
																Vegetables, number of portions	self report questionnaire
																Fruits availability	self report questionnaire
																Vegetables availability	self report questionnaire
																sweetened beverages, number of p	self report questionnaire
																sweetened beverages availability	self report questionnaire
																Soda, number of portions	self report questionnaire
																Soda availability	self report questionnaire
																Chocolates and candy,number of p	self report questionnaire
																Chocolates and candyavailability	self report questionnaire
																Snacks containing fatand salts, nur	self report questionnaire
																Snacks containing fatand salts avai	self report questionnaire
																outdoors playing (h/day)	self-report questionnaire
																physical education (h/week)	self-report questionnaire
																supervised sports or dance (h/wee	self-report questionnaire
																Availability sport venues	
																TV watching (h/day)	self-report questionnaire
																Computer and videogames (h/day)	self-report questionnaire
																Sitting (h/day)	self-report questionnaire
Banchonhattakit	2009	Southeast Asian J Trop	Thailand	RCT	Nutrition	180	10,5	0,6	51	195	10,7	0,5	52	8 monhts		Weight status %	calculted using IOTF BMI cutoff points
				Quasi-experimental	Physical activity											Frequency of consumption of fast	self-report questionnaire
																Knowledge towards obesity prever	self-report questionnaire
																Attitude towards obesitypreventiv	self-report questionnaire
																Intention towards obesitypreventiv	self-report questionnaire
																Amount of physical activity	self-reported questionnaire
																Amount of physical activity	observed by researchers
																Exercise for at least 30 mindays/we	self-reported questionnaire
Barnes	2021	Preventive medicine	Australia	RCT	Physical Activity	3 (nutrition only)	5-12-yr-olds	Not reported	49	167	5-12-yr-olds	Not reported	54	9 months	Pre and 9 mo post inte	BMI	objectively measured
					Nutrition	283 (PA only)			49							BMI z-score	objectively measured
						202 (combined)			56							Waist circumference	objectively measured
																BMI categories (underweight, norr	objectively measured, WHO cut-offs
																Quality of life, total score	self-report questionnaire, Paediatric Quality of Life Inventory
																functioning domain	self-report questionnaire, Paediatric Quality of Life Inventory
																functioning domain	self-report questionnaire, Paediatric Quality of Life Inventory
																domain	self-report questionnaire, Paediatric Quality of Life Inventory
Bartelink	2019	BMJ open	The Netherlands	Quasi experimental	Nutrition	Full HPSF = 537	7,6	2,2	52	661	7,6	2,1	53	4 years (and sustained i	Bl, 1 year, 2 years	BMI z-score	Objectively measured; z-score based on Dutch reference values
Willeboordse	2022	Plos ONE			Environment	artial HPSF = 478	7,4	2,2	53						Bl, 1-4 years	Waist circumference	Objectively measured
					Physical activity											Sedentary behaviour	Accelerometer-based
																Light physical activity	Accelerometer-based
																MVPA	Accelerometer-based
																School water consumption	self-report questionnaire
																Healthy dietary behaviours	self-report questionnaire
																Unhealthy dietary behaviours	self-report questionnaire
																Lunch intake - fruits (%)	self-report questionnaire
																Lunch intake - vegetables (%)	self-report questionnaire
																Lunch intake - grains(%)	self-report questionnaire
																Lunch intake - dairy (%)	self-report questionnaire
																Lunch intake - water (%)	self-report questionnaire
																Lunch intake - butter (%)	self-report questionnaire
																Lunch intake - minimum 2 healthy	self-report questionnaire
Bilic-Kirin	2013	Coll Antropol	Croatia	Controlled trial	Nutrition	200			49	172			49	6 months	Bl, 6 month	BMI	objectively measured, categorized according to Croatian child reference values
					Physical activity				Total group 49% girls mean age 1st grade							Weight	objectively measured
																Height (standing)	objectively measured
																Waist circumference	objectively measured, and
																Hip circumference	objectively measured, and
																Waist-to-hip ratio	objectively measured
																Waist-to-height ratio	objectively measured
																Weight status %	based on BMI, WC, HC and ratios
Bilińska, I. and Ł. Kryst,	2017	Anthropologischer Anz	Poland	RCT	Physical activity	3575	9,05			1718	8,99			1 yr	Bl, 1 year	Body height (standing)	Objectively measured
					Nutrition											Body weight	Objectively measured
																Prevalence of overweigth/obesity	Calculated based on the cut-off points of the IOTF values
																BMI	Force – IOTF) (Cole . 2000)
Brandstetter	2012	Obes Facts	Germany	RCT	Physical activity	450	7,6	0,4	45	495	7,5	0,4	48	1 year		BMI	objectively measured
					Nutrition											BMI z-core	calcuted using German reference values
																Waist circumference	objectively measured
																Skinfold thickness, triceps	objectively measured
																Skinfold thickness, subscapular	objectively measured
																frequency of consuming sugar-swe	questionnaire, proxy-report
																frequency of consuming sugar-swe	questionnaire, proxy-report
																frequency of playing outdoors	questionnaire, proxy-report
																frequency of participating in club s	questionnaire, proxy-report
																frequency of participating in sport	questionnaire, proxy-report
																time spent watching TV on weekda	questionnaire, proxy-report
																time spent watching TV on weeker	questionnaire, proxy-report
Breheny	2020	of Obesity	UK	RCT	Physical Activity	1153	8,8	1,1	47,6	1127	8,8	1,1	47,5	12 months	Pre, after 4 months an	BMI z-score	objectively measured, British growth reference data
																% body fat	objectively measured
																9D)	self-report questionnaire
																Wellbeing Index score (Middle yea	self-report questionnaire
																Academic attainment overall	teacher-rated
																Academic attainment, maths	teacher-rated
																Academic attainment, reading	teacher-rated
																Academic attainment, writing	teacher-rated
																Aerobic fitness	objectively measured (50-m run)
Breslin	2012	Arch Dis Child	UK	Controlled trial	Physical activity	209	9,1	0,4		207	9,1	0,4		12 weeks	Bl, 12 week	BMI	objectively measured
									Total group: 52% girls							MVPA (min/day)	self-report questionnaire
																Time spent in light physicalactivitie	objectively measured with
																Time spent in moderate physical a	objectively measured with
																Time spent in vigorous physical act	objectively measured with
																Time spent in MVPA	objectively measured with accelerometer
																time spent watching TV	self-report questionnaire Health Promotion Agencies National Children’s
																time spent watching DVD	self-report questionnaire Health Promotion Agencies National Children’s
																time spent playingcomputer/videc	self-report questionnaire Health Promotion Agencies National Children’s
																Time spent in sedentaryactivities	objectively measured with
																non-core food intake	self-report questionnaire The

																sweetened beverage intake	self-report questionnaire The
																sweetened beverages intake witho	self-report questionnaire The
																water intake	self-report questionnaire The
																fruit intake	self-report questionnaire The
																vegetable intake	self-report questionnaire The
																Environment (school or home) in v	self-report questionnaire The
																healthy eating behaviour	self-report questionnaire The
																attitude towards fruit	self-report questionnaire The
																attitude towards vegetables	self-report questionnaire The
Bundy, A	2017	The Journal of school h	Australia	Cluster RCT	Physical activity	113	6	0,6		108	6	0,6		13 weeks	Bl, 13 weeks	Total activity counts	Objectively measured with accelerometers
																Time spent in sedentary PA	Objectively measured with accelerometers
																Time spent in light PA	Objectively measured with accelerometers
																Time spent in MVPA	Objectively measured with accelerometers
																Sedentary time	Objectively measured with accelerometers
																time spent in play	quantification.
																time spent in nonplay	quantification.
																Number of playmates/quantificati	children (PSPCSAYC) scales competence (physical and acedeminc) and accptence (peer and maternal).
																perceived competence/social acce	children (PSPCSAYC) scales competence (physical and acedeminc) and accptence (peer and maternal).
																social skills	Parent and teacher report, The social skills improveent system rating scale (SSISRS).
																Childrens activities after school	Parent reported, real-time activity diary (palm-pilot loaded with Experience sampling program ESP software)
Burgermaster	2017	Prev Sci	USA	RCT	Nutrition	604 (group 1)	10,5	0,5	52	301	10,9	0,6	48	1 year		BMI	objectively measured
					Physical activity	569 (group 2)	10,5	0,5	50							BMI z-scores	objectively measured and calculated based on CDC reference data
																intake fruit and vegetablefrequenc	self-report Food Health & Choices Questionnaire
																intake fruit and vegetable, portion	self-report Food Health & Choices Questionnaire
																intake sugar-sweetened beverage,	self-report Food Health & Choices Questionnaire
																intake sugar-sweetened beverage,	self-report Food Health & Choices Questionnaire
																intake processed packagedsnacks,	self-report Food Health & Choices Questionnaire
																intake processed packagedsnacks,	self-report Food Health & Choices Questionnaire
																intake fast food, frequency per we	self-report Food Health & Choices Questionnaire
																intake fast food, portion size	self-report Food Health & Choices Questionnaire
																Unhealthy Eating Behavior Portior	analyses
																Unhealthy Eating Behavior Freque	analyses
																Healthy Behavior	analyses
																physical activity, weekly frequency	self-report Food Health & Choices Questionnaire
																physical activity, duration of sessic	self-report Food Health & Choices Questionnaire
																recreational screen time,weekly fr	self-report Food Health & Choices Questionnaire
																recreational screen time,duration (	self-report Food Health & Choices Questionnaire
																Sedentary behaviour	analyses
Busch	2023	Journal of School Healt	The Netherlands	Controlled trial	Physical activity	2660	64,4	4,4	49	1198	126	4,3	49	3 years	Bl, 5 years	BMI z-score	Objectively measured and calculated using Cole criteria
					Nutrition		(months)				(months)					Weight categories	Objectively measured and calculated
					Environment											Shifts in weight categories	Objectively measured and calculated
					other												
Caballero	2003	Am J Clin Nutr	USA	RCT	Physical activity	879				825				3 years		height (cm) (standing)	objectively measured
					Nutrition					Total group mean age 7.6 (0.6)						weight (kg)	objectively measured
																BMI	objectively measured
																BMI percentiles	categorised according to CDC
																percentage body fat	estimated from bioelectrical
																Skinfold thickness, triceps	objectively measured
																Skinfold thickness, subscapular	objectively measured
																PA, over 24h	self-report questionnaire
																PA, over 24h	measured with accelerometer
																Total energy intake over 24h	24-h recall, self-report
																Total energy intake during school l	by direct observation, and
																percentage of energy from fat, ove	24-h recall, self-report
																percentage of energy from fat, at s	by direct observation
																Knowlegde on PA and diet	self-report questionnaire,
																Self-efficacy to be physically active	self-report questionnaire,
																Self-efficacy to choose healthy foo	self-report questionnaire,
																healthy food choice intentions	self-report questionnaire,
Canaway	2019	Plos one	United Kingdom	Cluster RCT	Nutrition	622	6,3	0,3	51	735	6,3	0,3	47	12 months	Baseline and 3 and 18	Health-related quality of life QALY:	CHU-9D
					Physical activity												
Cao	2015	Am J Prev Med	China	RCT	Nutrition	965	7	0,4	45	889	6,8	0,2	47	2.7 year	Bl, 1 year, 2 year, 3 yea	BMI z-scores	calculated using WHO growth
					Physical activity											Weight status %	categorised according to
																BMI parents	self-report questionnaire
																BMI category parents	BMI <24 (normal weight), 24-28
Cao	2019	International journal o	China	Cluster RCT	Other	1287	7	0,41		1159	6,82	0,24		3 years	Bl, 3 year, 4 year, 5 yea	BMI z-score	Objectively measured and calculated
																Obesity prevalence	Direct measureements, standard cut-point
					Nutrition											Overweight prevalence	Direct measureements, standard cut-point
Centeio	2018	Preventive Medicine	USA	RCT	Other	377	9,9	0,02		251	10,01	0,02		8 months	Bl, 8 month	WHtR (Waist-to-Height Ratio)	Caculated by dividing waist circumference by height.
					Physical activity											BMI	calculated using WHO BMI cutpoints for age and seks. (de Onis and Lobstein, 2012, WHO 2017)
					Nutrition											BMI categories	WHO cutpoints for age and seks
Centis	2012	Pediatr obes	Italy	Controlled trial	Nutrition	103	9,3	0,3	51	106	9,4	0,4	53	8 months	Bl, 8 month	weight (kg)	objectively measured
					Physical activity											weight z-scores	calculated using Italian data
																height (cm) (standing)	objectively measured
																height z-scores	calculated using Italian data
																BMI	objectively measured
																BMI z-scores	calculated using Italian data,
																waist circumference	objectively measured
																hip circumference	objectively measured
																waist-to-hip ratio	objectively measured
																waist-to-height ratio	objectively measured
																Skinfold thickness, triceps	objectively measured
																Distribution overweight/obesity (w	calculated using Italian data,
																arm circumference	objectively measured
																sports habits	interview with parents
																leisure-time activities h/week	interview with parents
																time spent in open-air games	interview with parents
																attendence extra-schoolsports	interview with parents
																Extra-school physical	interview with parents
																mode of transportation toschool	interview with parents
																Parents' physical activity	interview with parents
																TV watching at home, h/day	interview with parents
																TV watching at home, total hours p	interview with parents
																TV on when eating	interview with parents

Chawla, N	2017	Journal of Ayub Medic	Thailand	Cluster RCT	Other	236	9,73			254	10			6 months	Bl, 6 month	overall knowledge on PA and diet	self-administered questionnaire (total 69 items)
					Nutrition											Overall attitude on PA and diet	self-administered questionnaire
					Physical activity											Diet practice	self-administered questionnaire
					Environment											PA practice	self-administered questionnaire
																Overall practive (Diet and PA)	self-administered questionnaire
																Phyical activity (hrs/wk)	self-administered questionnaire
																Screen time (hrs/day)	self-administered questionnaire
																Sleep (hrs/day)	self-administered questionnaire
																weight-for-height z-scores	was calculated by the intitute of nutrition research, Mahidol program (INMU) Thai Growth Program as weight for height
																Lipid profile - total cholesterol leve	3 ml of venous blood after 10-12 hours fasting. By the enzymatic method (Beckham Couter, AU680)
																Lipid profile - triglycerides levels	4 ml of venous blood after 10-12 hours fasting. By the enzymatic method (Beckham Couter, AU680)
																Lipid profile - LDL-C levels	5 ml of venous blood after 10-12 hours fasting. By the enzymatic method (Beckham Couter, AU680)
																Lipid profile - HDL-C levels	6 ml of venous blood after 10-12 hours fasting. By the enzymatic method (Beckham Couter, AU680)
Chesham	2018	BMC medicine	Scotland	Cluster RCT	Physical activity	252	8,1	2		127	8,8	1,8		6 months	Bl, 6 month	Average daily MVPA	Objectively measured with accelerometers
																average daily sedentary behaviour	Objectively measured with accelerometers
																VO2max, Total shuttle distance (m	Objectively measured with multistage Fitness test CD (Sports Coach UK, UK), 20-m shuttle run fitness test performance
																Sum of skinfolds (mm)	Objectively measured via sum of skinfolds at four sites, sandard ISAK procedures.
																Weight	Objectively measured
																height (cm) (standing)	Objectively measured
																BMI	Calculated
																BMI z scores	Based on UK 1990 reference data
																Weight categories	Calculated
																% meeting PA guidelies	Based on accelerometer data
Cohen	2014	J Acad Nutr Diet	USA	RCT	Nutrition	251	8,6	1,5	53	181	8,7	1,7	59	2 years	Bl, 2 year	intake fruits (cups), per 1,000 kcal	self-report 24h questionnaire
					Environment											intake vegetables, excl potatoes (c	self-report 24h questionnaire
Coknaz	2019	European Journal of Pe	Turkey	RCT	physical activity	53	9,6	1	55	53	10,3	1,2	59	12 weeks (50-60 min of	Pre and post	Weigth	Objectively measured
					Other (active video											Weigth z-score	Objectively measured
																BMI	Objectively measured
																BMI z-score	Objectively measured
																Fat ratio (%)	Objectively measured, based on skinfold measurements
																Visual reaction time, dominant har	Objectively measured, response to visual stimuli
																Visual reaction time, non-dominan	Objectively measured, response to visual stimuli
																Auditory reaction time, dominant	Objectively measured, response to auditory stimuli
																Auditory reaction time, non-domir	Objectively measured, response to auditory stimuli
																Self-perception	Children and youth physcial self-perception profile
																Global sel-worth	Subscale of Children and youth physcial self-perception profile
																Physical self-worth	Subscale of Children and youth physcial self-perception profile
																PA enjoyment	Self-report, PACES-SF
Cunha	2013	Plos One	Brazil	RCT	Nutrition	242	11,2	1,3		236	11,2	1,3		9 months	Bl, 6 month, 9 month	weight (kg)	objectively measured
																height (cm) (standing)	objectively measured
																BMI	objectively measured
																BMI z-scores	calculated based on WHO data
																percentage body fat	bioimpedance analysis
																weight status	classified according to WHO
																intake sugar-sweetened beverages	self-report questionnaire,
																intake sodas (ml/day)	self-report questionnaire,
																intake juices (ml/day)	self-report questionnaire,
																intake cookies (g/day)	self-report questionnaire,
																intake fruits (g/day)	self-report questionnaire,
																intake beans (g/day)	self-report questionnaire,
																intake fast food (consumed in resta	self-report questionnaire,
																intake milk (ml/day)	self-report questionnaire,
																intention of parents tochange eati	self-report questionnaire for
Dagger	2018	Pediatric exercise scier	UK	RCT	Other	26	10,64	0,06		27	10,62	0,06		20 weeks	Bl, 20 week, 28-30 wee	BMI z-score	stadiometer and electronic scales
					Physical activity											Waist circumference	non-elastic anthropometric tape
					Nutrition											VO2 peak (cardiorespiratory fitnes	individually calibrated continuous incremental treadmill and accelerometer (right hip) and HR monitor.
																Whole Body fat mass (kg)	DEXA scan
																Whole body fat percentage	DEXA scan
																Trunk Fat mass (kg)	DEXA scan
																Trunk fat percentage	DEXA scan
																peripheral fat mass (kg)	DEXA scan
																peripheral fat percentage	DEXA scan
																Whole lean body mass (kg)	DEXA scan
																whole lean body percentage	DEXA scan
																BMC (kg)	DEXA scan
																BMD (g/cm2)	DEXA scan
Dalma	2019	European journal of pu	Greece	Cluster RT	Nutrition	1442	8,3	1,6		0	0	0		8 months	Bl, 8 month	food insecurity level (raw scores) c	parent-report questionnaire, measured by FSSM
						986	8,5	1,6								Health related quality of life	Parental questionaire
																Weight categories	
Dämon	2005	Acta Pediatr	Austria	Controlled trial	Nutrition	260				231				10 months		BMI	objectively measured
									Total groupmean age 10-12							Eating habits	self-report questionnaire, and
																intake of unfavourable food	3-d dietary record
																Knowledge on nutrition	self-report questionnaire
Da Silva	2013	Eur J Clin Nutr	Brazil	Quasi-experimental	Nutrition	108				130				28 weeks	Bl, 28 week	% body fat	sum of two skinfold measures
					Physical activity				Total groupmean age 6-11 years							BMI	objectively measured
																BMI z-scores	WHO growth references
																weight status %	WHO growth references
																light physical activity (min/day)	self-report recall questionnaire
																moderate physical activity (min/da	self-report recall questionnaire
																intense physical activity (min/day)	self-report recall questionnaire
																aerobic cardiovascular endurance	PACER—progressive aerobic cardiovascular endurance run
																strength and resistance of the upp	push-up test
																abdominal strength and resistance	curl-up test
																flexibility	back-saver sit and reach test
																TV watching (h/day)	self-report recall questionnaire
Davis	1993	Ann N Y Acad Sci	USA	RCT	Nutrition	n= 674-876				n= 375-441				One group of children followed 1 year, for 5 ye		BMI	objectively measured and
					Physical activity				Total group 50% girlsmean age 9-13							BMI percentiles	calculated
																skinfold thickness, triceps	objectively measured
																Excersice habits	self-report questionnaire
																Eating habits, e.g. use of butter on	self-report questionnaire
																Knowledge on the cardio-vascular	self-report questionnaire
Davis	2021	International Journal o	US	RCT	Nutrition	1212	9,3	0,07	54	1509	9,2	0,07	52	9 months	Pre and post intervent	Height	objectively assessed
																Weight	Objectively assessed
																BMI	Objectively assessed

																BMI-z	Objectively assessed
																BMI-percentile	Objectively assessed, CDC values
																Waist circumference	Objectively assessed
																Body fat (%)	Objectively assessed
																Systolic Blood Pressure	Objectively assessed
																Diastolic Blood Pressure	Objectively assessed
																Vegetable intake (freq/d)	Self-report questionnaire (School Physical Activity & Nutrition (SPAN) dietry screener)
																Fruit intake (freq/d)	Self-report questionnaire (School Physical Activity & Nutrition (SPAN) dietry screener)
																Sugar sweetened beverages (freq/	Self-report questionnaire (School Physical Activity & Nutrition (SPAN) dietry screener)
																BMI status (underweight, normal v	Objectively assessed
Demir	2021	Journal of Pediatric nu	Turkey	Quasi-experimental	Physical activity	55	10	0,27	46	73	10,1	0,29	48	2 schoolyears	Pre and post intervent	Food addiction	Self-report questionnaire
					Nutrition											BMI status (underweight, normal v	Objectively assessed, WHO
																Eating behavior score	Self-report questionnaire
																Over-eating and food addiction	Yale food addiction scale (adapted for children)
																Physical activity score	Self-report questionnaire
de Greeff	2016	J Sch Health	The Netherlands	RCT	Physical activity	181	8	0,7	55	195	8,2	0,8	59	22 weeks		BMI	objectively measured, and
																cardiovascular fitness, speed-coor	10x5 m shuttle run, in seconds
																cardiovascular fitness, cardiorespir	20 m endurance shuttle run, in
																muscular fitness, explosive strengt	standing broad jump, in cm
																muscular fitness, muscular endura	number of sit-ups in 30 s
																muscular fitness, static strength	handgrip strength in kg
de Henauw	2015	Obes Rev	Belgium	Quasi-experimental	Physical activity	5727	6,1	1,8		5314	6	1,8		2 years		BMI	objectively measured
					Nutrition											BMI z-scores	categorised based on IOTF
					other											weight status (and %)	categorised based on IOTF
																percentage body fat	calculated from skinfold measurements (sum of two sites)
																Body fat mass (kg)	objectively measured
																Body fat (peripheral/central)	objectively measured
																Body fat (central/peripheral)	objectively measured
																bioelectrical impedance	Leg-to-leg bioelectrical impedance [ohm]
																waist circumference	objectively measured
																hip circumference	objectively measured
																waist-to-hip ratio	calculated
																waist-to-height ratio	calculated
de Moraes	2017	Preventive medicine	Brazil	RCT	Nutrition	238	10,9	0,83		240	10,8	0,73		1 year	Bl, 8 months	Glucose (fasting) (mg/dL)	Blood samples after 10-12 hours of fasting. Measures obtained by enzymatic-colorimetric metod.
																Total cholesterol (mg/dl)	Blood samples after 10-12 hours of fasting. Measures obtained by enzymatic-colorimetric metod.
																Frequency of impaired fasting gluc	Calculated
																Frequency of moderately high tota	Calculated
																Frequency of hypercholesterolem	Calculated
																Total energy (kcal/day)	24-hr recall
																Carbohydrate (g/day)	24-hr recall
																Protein (g/day)	24-hr recall
																Lipids (g/day)	24-hr recall
																Soft drinks (g/day)	24-hr recall
																Fruit juices (g/day)	24-hr recall
Delaney	2017	Am J Clin Nutr	Australia	Cluster RCT	Nutrition	581	5 to 12			861	5 to 12			2 month	Bl, 2 month	Student lunch order - energy (kj)	Objectively measured via Online school-canteen ordering system
																Student lunch order - saturated fat	Objectively measured via Online school-canteen ordering system
																Student lunch order - suger (g)	Objectively measured via Online school-canteen ordering system
																Student lunch order - sodium (mg)	Objectively measured via Online school-canteen ordering system
																student lunch order - % of energy f	Objectively measured via Online school-canteen ordering system
																student lunch order - % of energy f	Objectively measured via Online school-canteen ordering system
																Nutritional quality of student onlin	Objectively measured via Online school-canteen ordering system
																Student lunch order - items that w	Objectively measured via Online school-canteen ordering system
																Student lunch order - items that w	Objectively measured via Online school-canteen ordering system
																mean weekly online canteen rever	Automatically collected by online canteen
																Acceptability of the intervention	Telephone reported
																Availability of menu items	Canteen data
																User engagement with the interve	Canteen data
de Ruyter	2012	NEJM	The Netherlands	RCT	Nutrition	319	8,2	1,8	46	322	8,2	1,8	47	18 months		Weight (kg)	objectively measured
																Height (cm)	objectively measured
																Height z score	calculated based on Dutch
																BM z-score	calculated based on Dutch
																Waist circumference (cm)	objectively measured
																Waist-to-height ratio (%)	
																Sum of four skinfolds (biceps, trice	objectively measured
																Fat mass (kg)	electrical impedance
																Fat mass (% of body weight)	electrical impedance
																Urinary Sucralose Concentration (r	liquid chromatography with
DeVault	2009	J Am Diet Assoc	USA	Controlled trial	Nutrition	71	10,1	range 9.1-11.4	44	69	9,9	range 9.2-11.7	59	9 weeks		Consumption total fat in previous c	CATCH Food Checklist, self-
					Physical activity											Consumption saturated fat in prev	CATCH Food Checklist, self-
																Consumption sodium in previous c	CATCH Food Checklist, self-
																Most commonly reported foods	CATCH Food Checklist, self-
																Food self-efficacy	Knowledge, Attitudes, and
																Food choice intentions	KAB questionnaire, self-report assisted by teacher
																Which food has more fat?	KAB questionnaire, self-report assisted by teacher
																Physical activity self-efficacy	KAB questionnaire, self-report assisted by teacher
																Physical activity knowledge	KAB questionnaire, self-report assisted by teacher
																Nutrition curriculum knowledge	KAB questionnaire, self-report assisted by teacher
																Attempted weight loss	KAB questionnaire, self-report assisted by teacher
																Healthy body size perception, gend	KAB questionnaire, self-report assisted by teacher
																Healthy body image	KAB questionnaire, self-report assisted by teacher
Diao	2020	Health and Quality of L	China	RCT	Nutrition	518	11,4	1,8	49	430	11,4	1,8	50			BMI	Objectively measured
					Physical activity		(INT + CON)				(INT + CON)					Quality of Life , total score	Questionnaire, self-report
																Quality of Life, physical dimension	Questionnaire, self-report
																Quality of Life, psychological dimen	Questionnaire, self-report
																Quality of Life, social dimension	Questionnaire, self-report
																Quality of Life, pubertal dimension	Questionnaire, self-report
Di Maglie	2021	Physiol Rep	Italy	Controlled trial	Physical Activity	80	12.1	0,5 (int+con)	51,3 (int+co	80	12,1 (int+cont)	0,5 (int+con)	51,3 (int+con)	6 months	Pre and post intervent	BMI percentile	objectively measured
																Proportion overweight,	objectively measured, CDC cut-offs
																Waist circumference	objectively measured
																Waist-to-height ratio	objectively measured
																Physical education/sport	parent-report questionnaire
																Hours of television viewing	parent-report questionnaire
																Hours spent on the computer	parent-report questionnaire
																Hours spent playing electronic	parent-report questionnaire

																Physical fitness - vertical jump	objectively assessed
																Physical fitness - standing broad ju	objectively assessed
																Physical fitness - rope jumps/min	objectively assessed
Dong	2021	Frontiers in Pediatrics	China	Controlled trial	Nutrition	25702	10,3	3,2	49,3	23175	11,3	3,3	49,6	6 months	Pre and post intervent	BMI, binominal (increase/decreas	objectively measured
					Physical activity											BMI value	objectively measured
					Environment											BMI z-scores,	objectively measured, CDC charts
																SBP,	objectively measured
																SPB z-scores, binominal (increase/	objectively measured
																DBP	objectively measured
																DBP z-scores, binominal (increase/	objectively measured
																% overweight/obesity, binominal (	objectively measured
Donnelly	2009	Prev Med	USA	RCT	Physical activity	814	8,3	0,4		713	8,2	0,4		3 years		BMI	objectively meaured
										Total group 52% girls						BMI percentiles	calculated based on CDC
																Physical activity levels, avarage 4 d	measured with accelerometer
																PA levels, Weekday (counts/min)	measured with accelerometer
																PA levels, Weekend day (counts/m	measured with accelerometer
																PA levels, During school (8 AM–2:5	measured with accelerometer
																PA levels, After school (3 PM–5:59	measured with accelerometer
																PA levels, Evening (6 PM–11 PM) (c	measured with accelerometer
																Minutes of MVPA (≥4 METs)	measured with accelerometer
																Intensity of classroom PA	System for Observation of
																Teacher participation in classroom	PA
																Reading skills	Wechsler Individual Achievement Test-2nd Edition
																Writing skills	Wechsler Individual Achievement Test-2nd Edition
																Mathematics skills	Wechsler Individual Achievement Test-2nd Edition
																Oral language skills	Wechsler Individual Achievement Test-2nd Edition
Donnelly	1996	Obes Res	USA	Controlled trial	Physical activity	102				236				2 years		percentage body fat	calculated
					Nutrition					Total groupmean age grade 3-5						height (cm)	objectively measured
																weight (kg)	objectively measured
																BMI	objectively measured
																Energy (kCal) school lunch	school-menu analysis,
																Tota energy 24h (kCal)	24h recall self-report
																Protein (g) school lunch	school-menu analysis, and 24h
																Total protein (g) 24 h	24h recall self-report
																Carbohydrate (g) school lunch	school-menu analysis, and 24h
																Total carbohydrate (g) 24h	24h recall self-report
																Fat (g) school lunch	school-menu analysis, and 24h
																Total fat (g) 24h	24h recall self-report
																Natrium (mg) school lunch	school-menu analysis, and 24h
																Total natrium (mg) 24h	24h recall self-report
																Fiber (g) school lunch	school-menu analysis, and 24h
																total fiber (g) 24h	24h recall self-report
																Nutrinion knowledge	test administered by teacher
																Physical activity outsideof school	self-report checklist
																Physical activity levels in classroom	measured with SOFIT
																physical fitness	one-mile walk/run
																resting systolic blood pressure	
																resting diastolic blood pressure	
																amount of oxygen consumed	measured with metabolic cart
																Peak aerobic capacity (maximum c	measured with metabolic cart
																lipids	blood sample analysis
																insulin/glucose levels	blood sample analysis
																iron	blood sample analysis
																ferritin	blood sample analysis
																Plasma triglycerides	blood sample analysis
																cholesterol	blood sample analysis
																HDL-cholesterol	blood sample analysis
Dring	2022	Scientific Reports	United Kingdom	Controlled trial	Physical activity	44				35				5 weeks	Bl, 5 weeks	Body mass (kg)	objectively measured
							Total group: 10.3 (0.8) years									BMI (kg/m2)	objectively measured and calculated
																BMI centile	objectively measured and calculated using British growth reference data
																Waist circumference (cm)	objectively measured
																Hip circumference (cm)	objectively measured
																Sum of skinfolds (mm) (4 skinfolds	objectively measured
																Multi-stage fitness test	objectively measureed
																Strooptest, response time (ms)	ojectively measured (cognitive functioning test)
																Strooptest, accuracy (%)	ojectively measured (cognitive functioning test)
																Sternberg paradigm, response tim	ojectively measured (cognitive functioning test)
																Sternberg paradigm, accuracy (%)	ojectively measured (cognitive functioning test)
																Flanker task, response time (ms)	ojectively measured (cognitive functioning test)
																Flanker task, accuracy (%)	ojectively measured (cognitive functioning test)
Drummy	2016	J Paediatr Child Health	Ireland	RCT	Physical activity	54				53				12 weeks		height (m)	objectively measured
										Total group: 9.5 years						weight (kg)	objectively measured
																BMI	objectively measured
																Sum of four skinfolds (biceps, trice	objectively measured
																weekday MVPA (min)	measured with accelerometer
El Harake	2018	Nutrients	Lebanon	RCT	Nutrition	114	11,16	0,31		89	10,89	0,47		2 years	Bl, 6 month, 1 year, 2 y	Nutrition knowledge score	Attitude and behaviour, 32-points-) questionnaire
																Nutrition attitude score	scale agree-disagree) in the KAB (knowledge, Attitude and behaviour, 32-points-) questionnaire
																Dietary and lifestyle behaviours sc	(knowledge, Attitude and behaviour, 32-points-) questionnaire
																Waist circumference	Objectively measured
																BMI for age z-score (BAZ)	Calculated age and gender specific, using WHO Antro Plus software
																Height-for-age z-scores (HAZ)	Calculated age and gender specific, using WHO Antro Plus software
																Weight-for-age z-scores (WAZ)	Calculated age and gender specific, using WHO Antro Plus software
																Waist-to-Height Ratio (WHtR)	Calculated by dividing waist circumference by height (cut off point 0,5)
																Dietary intake - energy intake (Kca	24-hr recall (During Face-to-face interviews with children and their mothers (as proxy respondents))
																Dietary intake - Macronutrients - C	24-hr recall (During Face-to-face interviews with children and their mothers (as proxy respondents))
																Dietary intake - Macronutrients - S	24-hr recall (During Face-to-face interviews with children and their mothers (as proxy respondents))
																Dietary intake - Macronutrients - D	24-hr recall (During Face-to-face interviews with children and their mothers (as proxy respondents))
																Dietary intake - Macronutrients - p	24-hr recall (During Face-to-face interviews with children and their mothers (as proxy respondents))
																Dietary intake - Macronutrients - T	24-hr recall (During Face-to-face interviews with children and their mothers (as proxy respondents))
																Dietary intake - Macronutrients - M	24-hr recall (During Face-to-face interviews with children and their mothers (as proxy respondents))
																Dietary intake - Macronutrients - P	24-hr recall (During Face-to-face interviews with children and their mothers (as proxy respondents))
																Dietary intake - Macronutrients - S	24-hr recall (During Face-to-face interviews with children and their mothers (as proxy respondents))
																Dietary intake - Macronutrients - T	24-hr recall (During Face-to-face interviews with children and their mothers (as proxy respondents))
																Dietary intake - Micronutrients - V	24-hr recall (During Face-to-face interviews with children and their mothers (as proxy respondents))

																Dietary intake - Micronutrients - V	24-hr recall (During Face-to-face interviews with children and their mothers (as proxy respondents))
																Dietary intake - Micronutrients - V	24-hr recall (During Face-to-face interviews with children and their mothers (as proxy respondents))
																Dietary intake - Micronutrients - V	24-hr recall (During Face-to-face interviews with children and their mothers (as proxy respondents))
																Dietary intake - Micronutrients - V	24-hr recall (During Face-to-face interviews with children and their mothers (as proxy respondents))
																Dietary intake - Micronutrients - V	24-hr recall (During Face-to-face interviews with children and their mothers (as proxy respondents))
																Dietary intake - Micronutrients - V	24-hr recall (During Face-to-face interviews with children and their mothers (as proxy respondents))
																Dietary intake - Micronutrients - V	24-hr recall (During Face-to-face interviews with children and their mothers (as proxy respondents))
																Dietary intake - Micronutrients - V	24-hr recall (During Face-to-face interviews with children and their mothers (as proxy respondents))
																Dietary intake - Micronutrients - V	24-hr recall (During Face-to-face interviews with children and their mothers (as proxy respondents))
																Dietary intake - Micronutrients - F	24-hr recall (During Face-to-face interviews with children and their mothers (as proxy respondents))
																Dietary intake - Micronutrients - Ir	24-hr recall (During Face-to-face interviews with children and their mothers (as proxy respondents))
																Dietary intake - Micronutrients - Zi	24-hr recall (During Face-to-face interviews with children and their mothers (as proxy respondents))
																Dietary intake - Micronutrients - C	24-hr recall (During Face-to-face interviews with children and their mothers (as proxy respondents))
																Dietary intake - Micronutrients - Pl	24-hr recall (During Face-to-face interviews with children and their mothers (as proxy respondents))
																Dietary intake - Micronutrients - M	24-hr recall (During Face-to-face interviews with children and their mothers (as proxy respondents))
																Dietary intake - Micronutrients - S	24-hr recall (During Face-to-face interviews with children and their mothers (as proxy respondents))
																Dietary intake - Micronutrients - P	24-hr recall (During Face-to-face interviews with children and their mothers (as proxy respondents))
Elder	2014	Salud Publica Mex	USA, Mexico	Controlled trial	Nutrition	368				373				10 weeks		using available water sources	Classroom Direct Observation
										Total groupmean age grade 1-6						having a water bottle with water o	Classroom Direct Observation
																water consumption at lunch time	Classroom Direct Observation
																water consumption in class	Classroom Direct Observation
																types of drinks consumed during lu	Classroom Direct Observation
																easily accessible water source in cl	Classroom Direct Observation
																teacher allowed time to leave the	Classroom Direct Observation
																presence of classroom material or	Classroom Direct Observation
																available water sources in the scho	School Environment Direct
																quality/cleanliness of the water	School Environment Direct
																usability of water fountains and ot	School Environment Direct
																number of students in line for the	School Environment Direct
																number of students that used the	School Environment Direct
																how long each student drank from	School Environment Direct
Elinder	2012	Int J Behav Nutr Phys A	Sweden	Controlled trial	Nutrition	471				328				2 years	Bl, 2 year	BMI z-score	calculated based on Swedisch reference charts
					Physical activity					Total groupmean age 6-16						weight status %	calculated based on IOTF cut-
																Consumption of breakfast (daily)	Health questionnaire,
																Consumption of lunch (daily)	Health questionnaire,
																Consumption of vegetables (2x dai	Health questionnaire,
																Consumption of fruit (2x daily)	Health questionnaire,
																Consumption of sweets (2x week)	Health questionnaire,
																Consumption of sweetened drinks	Health questionnaire,
																Membership of sport club	self-report questionnaire
																Sports participation (3x week)	self-report questionnaire
																Outside activities (30min/day)	self-report questionnaire
																Active transportation to school (wa	self-report questionnaire
																Physical activity during break (3 da	self-report questionnaire
																TV watching (3h/schoolday)	self-report questionnaire
																Self–esteem	self-report questionnaire
																Having a good feeling	self-report questionnaire
																Having one risk behaviour	self-report questionnaire
Fairclough	2013	BMC Public Health	UK	RCT	Nutrition	136	10,6	0,3		322	10,7	0,3		20 weeks		BMI	objectively measured and
					Physical activity											BMI z-score	off points
																waist circumference	objectively measured
																weight status %	off points
																LPA (min day)	measured with accelerometer
																MPA (min day)	measured with accelerometer
																VPA (min day)	measured with accelerometer
																aerobic fitness	20m shuttle run test
																Sedentary time (min/day)	measured with accelerometer
																Consumption of breakfast	self-report 24h dietary recall
																Consumption of fruits	self-report 24h dietary recall
																consumption of vegetables	self-report 24h dietary recall
Farmer	2017	Int J Obes	New Zealand	RCT	Physical activity	418	8	1,2	47	422	7,9	1,1	53	2 years		height (m)	objectively measured
																weight (kg)	objectively measured
																BMI	objectively measured and
																BMI z-scores	calculated based on WHO
																waist circumference	objectively measured
																waist-to-height ratio	objectively measured
																physical activity, counts per minute	measured with accelerometer
																minutes of moderate-to-vigorous	measured with accelerometer
																risk and challenge of play environn	direct observation
																engagement with natural element	direct observation
																ability to actively manipulate and	direct observation
																availability of wheeled play (for ex	direct observation
																availability of ball games	direct observation
																children to socialise with at play er	direct observation
																quality of independent access to p	direct observation
																Physical activiy school policies	self-report questionnaire by principals
Fetter	2018	Journal of the America	USA	RCT	Nutrition	54	9,5	0,4		38	9,6	0,4		9 months	Bl, 9 month	Sedentary behaviour	Objectively measured with accelerometers
					Physical activity											Physical activity - Easy PA	Objectively measured with accelerometers
																Physical activity - Moderate PA	Objectively measured with accelerometers
																Physical activity - Vigorous PA	Objectively measured with accelerometers
																Physical activity - Very vigorous PA	Objectively measured with accelerometers
																Physical activity - MVPA	Objectively measured with accelerometers
																mean BMI percentile	Measured (see note)
Ford	2013	J Sports Sci	UK	RCT	Physical activity	77				75				15 weeks	Bl, 15 week	height (m)	objectively measured
										Total group mean age 5-11 years						weight (kg)	objectively measured
																fat mass (kg)	objectively measured
																lean mass (kg)	objectively measured
																percentage body fat	calculated using the child
																BMI	objectively measured
																skinfold thickness, triceps	objectively measured
																skinfold thickness, calf	objectively measured
																skinfold thickness, abdominal	objectively measured
																skinfold thickness, suprailiac	objectively measured
																total body density	derived from skinfold thickness

																total body fat	derived from skinfold thickness
																mean weekday 9,00-15,00h physic	measured with accelerometer
																mean total weekday PA, in counts p	measured with accelerometer
																mean total weekend pA, in counts	measured with accelerometer
																total daily caloric intake (kcal/day)	self-report 3-Day Dietary Recall, with assistence from parents
Franckle, R.L	2017	Obesity (Silver Spring)	USA	RCT	Nutrition	nple at baseline)								2 years	(Pre-baseline 5 years e	BMI	Calculated with measured height and weight
					Physical activity											BMI percentiles	Standard definition (95 and above for childhood obesity) was used.
																Prevalence of obesity	Calculated
																SSB intake (soda and fruit punch),	Self-report questionnaire
																100% juice intake (1 or less times \	Self-report questionnaire
																Water intake (1 or more times yes	Self-report questionnaire
																Fruit and vegetable intake (5 times	Self-report questionnaire
																Screen time (< 2 hr daily)	Self-report questionnaire
																Sleep (10 hrs or more on a usual d	Self-report questionnaire
																physical activity (30 min. Or more	Self-report questionnaire
Fung	2012	Int J Behav Nutr Phys A	USA	RCT	Nutrition	293 (2008)			51	3645 (2008)			52	2 years		weight status (%)	categorised based on IOTF
Tran	2014	PLoS ONE			Physical activity	394 (2010)			57	3398 (2010)			51			Mean servings of fruits & vegetabl	self-report questionnaire
										Total group mean age 5th grade						Mean dietary energy intake (kcal)	self-report questionnaire
																overall diet quality (DQI-score)	self-report questionnaire, Diet
																MVPA levels over 7 dayperiod (PAC	self-report questionnaire
Gao	2017	Journal of sport and he	USA	RCT	Physical activity	85	8,27	0,7		79				1 year	Bl, 1 year, 2 year	Sedentary behaviour	Objectively measured with accelerometry
																Light Physical Activity	Objectively measured with accelerometry
																MVPA	Objectively measured with accelerometry
																Energy expenditure	Objectively measured with accelerometry
Gatto	2017	Pediatric obesity	USA	RCT	Other	172	9,3	0,9		147	9,3	0,9		12 weeks	Bl, 12 week	BMI percentile	Calculated using Centers for Disease Control and Prevention (CDC) cut-points for age and sex
					Nutrition											BMI z-score	Calculated using Centers for Disease Control and Prevention (CDC) cut-points for age and sex
																Waist circumference (cm)	Objectively measured using NHANES protocol
																Body fat percentage	Objectively measured
																Systolic blood pressure	Objectively measured
																diastolic blood pressure	Objectively measured
																Cholesterol total	Measured using enzymatic methods on a Stanbio Sirrus analyzer
																LDL-C	Measured using enzymatic methods on a Stanbio Sirrus analyzer
																HDL-C	Measured using enzymatic methods on a Stanbio Sirrus analyzer
																Tryglycerides	Measured using enzymatic methods on a Stanbio Sirrus analyzer
																Insulin (uU/ml)	Quantified using an ELISA kit
																HOMA-IR (Homeostatic model ass	Calculated as a measure of insulin resistance
																Glucose (mg/dL-1)	Assayed using a Yellow Springs Instruments analyzer
																Metabolic syndrome	Programme's criteria for adults
																Energy intake (kcal)	Food frequency questionnaire (Block Kids Food Screener, last week version)
																protein (g/day)	Food frequency questionnaire (Block Kids Food Screener, last week version)
																fat (g/day)	Food frequency questionnaire (Block Kids Food Screener, last week version)
																Carbohydrate (g/day)	Food frequency questionnaire (Block Kids Food Screener, last week version)
																Added sugar (tsp/day)	Food frequency questionnaire (Block Kids Food Screener, last week version)
																dietary fibre (g/day)	Food frequency questionnaire (Block Kids Food Screener, last week version)
																meat intake (OEd)	Food frequency questionnaire (Block Kids Food Screener, last week version)
																Dairy intake (CEe)	Food frequency questionnaire (Block Kids Food Screener, last week version)
																Whole grain intake	Food frequency questionnaire (Block Kids Food Screener, last week version)
																Vegetables intake (CE)	Food frequency questionnaire (Block Kids Food Screener, last week version)
																Fruit and fruit juice intake (CE)	Food frequency questionnaire (Block Kids Food Screener, last week version)
																Apples, bananas and oranges intak	Food frequency questionnaire (Block Kids Food Screener, last week version)
																Lettuce salad intkae (CE)	Food frequency questionnaire (Block Kids Food Screener, last week version)
																Green beans and peas intake (CE)	Food frequency questionnaire (Block Kids Food Screener, last week version)
																Tomatoes intake (CE)	Food frequency questionnaire (Block Kids Food Screener, last week version)
Gold	2017	Journal of school healt	USA	RCT	Nutrition	378	8,3	0,5		369	8,4	0,5		7 weeks	Bl, 7 week	Total fruit consumption	Self-report 13-item food frequency survey
																Total vegetables consumption	Self-report 13-item food frequency survey
																melon consumption	Self-report 13-item food frequency survey
																Berries consumption	Self-report 13-item food frequency survey
																Mixed fruit consumption	Self-report 13-item food frequency survey
																100% juice consumption	Self-report 13-item food frequency survey
																Dark green vegetables consumptic	Self-report 13-item food frequency survey
																Orange vegetables consumption	Self-report 13-item food frequency survey
																Starchy vegetables consumption	Self-report 13-item food frequency survey
																Beans and peas consumption	Self-report 13-item food frequency survey
																Snack consumption	Self-report 13-item food frequency survey
																Other vegetables consumption	Self-report 13-item food frequency survey
																Potato chips and friench fries cons	Self-report 13-item food frequency survey
Goswami	2021	International Journal o	Norway	Controlled trial	Nutrition	176	9,7	0,3	-	98	10	0,3	-	3 years	yearly	Muscle mass	objectively measured
					Physical activity											Bone mass	objectively measured
					Environment											Weight	objectively measured
																Weight (overweight)	objectively measured
																Height	objectively measured
																BMI	objectively measured
																BMI (overweight)	objectively measured
																Fat mass	objectively measured, bioimpedance
Graf	2005	J Sports Sci Med	Germany	Controlled trial	Physical activity	433	6,7	0,4	49	178	7,3	0,4	49	21 months		height (m)	objectively measured
Graf	2008	J Sports Sci												4 years		weight (kg)	objectively measured
																BMI	objectively measured and
																weight status %	based on German percentiles
																endurance performance,number c	6-minute run
																temporal coordination, number of	lateral jumping
																gross motor development	Balancing backwards
																gross motor development	One-legged obstacle jumping
																gross motor development	Sideways movements
																overall motor quotient	sum of the four excercises
Grao-Cruces	2016	Kinesiology	Spain	Controlled trial	Physical activity	66	11,3	0,5	64	76	11,5	0,5	49	12 weeks		BMI	objectively measured and
																number of steps a day, during scho	measured with pedometer
																number of steps a day, out-of-scho	measured with pedometer
																number of steps a day, during wee	measured with pedometer
																number of steps a day, during wee	measured with pedometer
																average number of steps a day, for	measured with pedometer
Grassi	2016	Public Health Nutr	Italy	Quasi-experimental	Nutrition	27			30	33			49	10 weeks	Bl, 10 week, 5.5 month	frequency of intake fresh fruits	self-report questionnaire
				Other						Total groupmean age 10 years						frequency of intake salad	self-report questionnaire
																frequency of intake raw vegetable	self-report questionnaire
																frequency of intake cooked vegeta	self-report questionnaire

																frequency of intake 100% fruit juic	self-report questionnaire
																self-efficacy for eating fruit and ve	self-efficacy scale
																motivation for healthy eating	motivation scale
																Children's perceived parental supp	1. Emotional Social Support scale
																parental behaviours to improve th	1. Perceived Parental Instrumental
																Parents' motivation for healthy eat	questionnaire for parents
																Parents' social support for their ch	questionnaire for parents
																behavioural food changes	focus group with children
																knowledge on food	focus group with children
																media knowledge	focus group with children
																media beliefs	focus group with children
																critical analysis	focus group with children
																expression skills	focus group with children
																reinforcement	focus group with children
																opinions on the intervention	focus group with children
																Fruit and vegetable availability at h	questionnaire for parents
																Fruit and vegetable accessibility at	questionnaire for parents
Greene	2019	American journal of pr	USA	Cluster RCT	Nutrition	1258				850				6 weeks	Bl, 6 week, 9 week	Fruit selection	Tray waste was observed and visually estimated by researchers
																Fruit consumption	Tray waste was observed and visually estimated by researchers
																Vegetable selection	Tray waste was observed and visually estimated by researchers
																Vegetable consumption	Tray waste was observed and visually estimated by researchers
																White milk selection	Tray waste was observed and visually estimated by researchers
																White milk consumption	Tray waste was observed and visually estimated by researchers
Grydeland	2014	Br J Sports Med	Norway	RCT	Nutrition	465	11,2	0,3	50	859	11,2	0,3	48	20 weeks		BMI	objectively measured and
					Physical activity											BMI z-score	calculated with WHO growth
																weight status %	calculated based on IOTF cut-
																waist circumference (cm)	objectively measured
																waist-to-height ratio	objectively measured and
Gutiérrez-Martínez	2018	Revista de saude publi	Colombia	RCT	Physical activity	, MARA+SMS 60)	10,4	0,7		67	10,6	0,8		10 weeks	Bl, 10 week	Mean minutes of daily MVPA	Objectively measured with accelerometers
																Fat percentage	abdominal perimeter and the middle third of the arm.
																BMI z-scores	Growth patterns of the WHO
																Frequency of PA during recess in tl	Objectively measured with accelerometers
																Most frequent activities during rec	Objectively measured with accelerometers
																MVPA on weekdays	Objectively measured with accelerometers
																Daily light PA	Objectively measured with accelerometers
																Light PA on weekdays	Objectively measured with accelerometers
																Daily physical inactivity	Objectively measured with accelerometers
																Physical inactivity on weekdays	Objectively measured with accelerometers
																Daily consumption of hih calorie fo	Self-report Questionnaire
																Daily consuption of fruits and vege	Self-report Questionnaire
Habib-Mourad	2014	BMC Public Health	Lebanon	RCT	Nutrition	193	10,3	0,9	43	181	10,1	1	47	3 months		height	objectively measured
				Feasibility	Physical activity											weight	objectively measured
																BMI	calculated
																waist circumference	objectively measured
																Breakfast intake	self-report questionnaire
																Number of snacks per day (3 or mo	self-report questionnaire
																Eating in front of TV	self-report questionnaire
																Eating out (3 or more times/week)	self-report questionnaire
																Chips consumption between meals	self-report questionnaire
																Chocolate consumption between r	self-report questionnaire
																Soft drinks consumption between	self-report questionnaire
																Sweetened drinks consumption be	self-report questionnaire
																Fruit consumption between meals	self-report questionnaire
																Sandwich consumption between n	self-report questionnaire
																Chips, bought from School shop	self-report questionnaire
																Chocolate, bought from School sho	self-report questionnaire
																Soft drinks, bought from School sh	self-report questionnaire
																Sweetened drinks, bought from Sc	self-report questionnaire
																Manoushe, bought from School sh	self-report questionnaire
																Croissant, bought from School sho	self-report questionnaire
																Playing at recess	self-report questionnaire
																After school Physical activity/ weel	self-report questionnaire
																Playing at home after-school	self-report questionnaire
																TV viewing during school days	self-report questionnaire
																TV viewing during week-end	self-report questionnaire
																Electronic games during schoolday	self-report questionnaire
																Electronic games during weekend	self-report questionnaire
																Nutrition Knowledge	self-report questionnaire
																Nutrition Self-efficacy	self-report questionnaire
Habib-Mourad	2020	BMC Public Health	Lebanon	RCT	Nutrition	701 and 699	9-12-yr-olds			344 and 532	9-12-yr-olds				Pre and post intervent	Dietary knowledge score (summar	self-report
					Physical activity											Dietary self-efficacy score (summa	self-report
																Daily breakfast intake	self-report
																Intake of fruits (at least twice per c	self-report
																Vegetable consumption (at least o	self-report
																Crisp consumption (at least once p	self-report
																Purchase of soft drinks (yes/no)	self-report
																Purchase of crisps (yes/no)	self-report
																Fruits consumed as snack (betwee	self-report
																Crisps consumed as snack (betwee	self-report
																After-school physical activity (at le	self-report
Habib-Mourad	2020	Nutrients	Lebanon	RCT	Nutrition	698	9,95	1,13	53,7	541	10,13	0,68	41,9	2 years	Pre, after 2 (post) and	BMI z-score	objectively measured
					Physical activity											OW/OB odds	objectively measured, WHO cut-offs
																Overweight/obesity prevalence	objectively measured, WHO cut-offs
																Fruit consumption	self-report questionnaire
																Raw veggies consumprion	self-report questionnaire
																Having chips	self-report questionnaire
																Drinking soft drinks	self-report questionnaire
																Drinking sweet drinks	self-report questionnaire
																Exercise after school	self-report questionnaire
																Physical activity after school	self-report questionnaire
																Nutrition knowledge score (sumsc	self-report questionnaire
																Self-efficacy score (self-confidence	self-report questionnaire
Habib-Mourad, 2023	Diff year f	Public Health Nutrition	Lebanon (2012), Jor	Cluster-randomised co	Dietary	6052	10,7	not reported	56,6	6200	10,5	not reported	60,4	one year	Pre (1 week before) an	consumption, ≥ twice/day (%)	
					Physical activity											twice/day (%)	

					Environment											three times/week (%)	
																Daily intake of breakfast (%)	
																Intake of fruits, ≥ twice/day (%)	
																(%)	
																Intake of chips, ≥ twice/day (%)	
																between meals (%)	
																chocolate, cookies, candiers,	
																Chips intake, between meals (%)	
																schop (y/n)	
																(y/n)	
																Nutrition knowledge score	
																Self-efficacy score	
																≥ twice/week (%)	
Hadi	2019	Pakistan Journal of Nu	Indonesia			31	9, 10 and 11 year olds		45	33	9, 10 and 11 year olds		52	6 months	Pre and post intervent	Food type (fastfood; non-fastfood)	Self-report questionnaire
																Meal frequency (excessive; good)	Self report questionnaire
																Food preference (like; dislike)	Self report quetsionnaire
																Physical activity (active; inaactive)	Self report quesitonnaire
																BMI	Objectively assessed
Harrison	2006	J Sci Med Sport	Ireland	Controlled trial	Physical activity	182	10,2	1,2	44	130	10,3	0,8	42	16 weeks		BMI	objectively measured and
																30 min blocks/day MVPA	Previous Day Physical Activity
																aerobic fitness (laps)	20m shuttle test
																30 min blocks/day screen time	Previous Day Physical Activity
																Physical activity self-efficacy	rating instrument
Hawkins	2018	International journal o	USA	RCT	Nutrition	1195	10,5	1,2		431	10,6	1,1		28 months	Bl, 18 month, 28 mont	weight	Objectively measured
																BMI percentile	Calculated using norms provided by Centers for Disease Control in 2000
																Energy selection (kcal/lunch)	Digital potography of foods method
																Energy consumtion (kcal/lunch)	Digital potography of foods method
																Plate waste energy (kcal/lunch)	Digital potography of foods method
																Sodium selection (mg)	Digital potography of foods method
																Sodium consumption (mg)	Digital potography of foods method
																Sodium plate waste (mg/lunch)	Digital potography of foods method
																Added sugar selection (tsp)	Digital potography of foods method
																Added sugar consumption (tsp)	Digital potography of foods method
																Added sugar plate waste (tsp/lunc	Digital potography of foods method
Hendy	2011	Appetite	USA	RCT	Nutrition	193			44	189			45	9 months		BMI	objectively measured and
					Physical activity					Total groupmean age grade 1-4						BMI percentiles	calculated based on CDC
																Consumption of low-fat low-sugar	direct observation of school
																Consumption of fruits and vegetab	direct observation of school
																number of steps	measured with pedometer
Herscovici	2013	Rev Panam Salud Publi	Argentina	RCT	Nutrition	216	9,6	0,8	53	171	9,8	0,7	47	6 months		BMI	objectively measured and
					Physical activity											BMI z-score	calculated based on CDC
																Consumption of vegetables	interview by dietitians
																Consumption of whole fruits	interview by dietitians
																Consumption of skim milk	interview by dietitians
																Consumption of low sugar cereals	interview by dietitians
																Consumption of orange juice	interview by dietitians
																Consumption sweets, 1/day	interview by dietitians
																Consumption of chocolate Consum	interview by dietitians
																consumption of salted packed sna	interview by dietitians
																consumption of sodas and sweete	interview by dietitians
																consumption of hotdogs/hamburg	interview by dietitians
Hollar (effective multi-	2010	J Health Care Poor Unc	USA	Quasi-experimental	Nutrition	3032				976				2 years		weight z-scores	objectively measured
Hollar (effect of 2 year..)	2010	Am J Public Health			Physical activity					Total group 51% girlsmean age 8 (4-13)						BMI z-scores	calculated based on CDC
Hollar (healthier options..)	2010	J Am Diet Assoc														BMI percentiles	calculated based on CDC
																weight status %	calculated based on CDC
																diastolic blood pressure	measured with electronic
																systolic blood pressure	measured with electronic
																change in math	Florida Comprehensive
																change in reading	Florida Comprehensive
Hopkins	2018	Public Health Nutrition	USA	RCT	Nutrition	(enhanced care)	7,35	0,33	54,05	21 (active contr	7,71	0,45	61,9	8 weeks	Pre and post intervent	Liked vegetables (food preference)	self-report questionnaire
					Physical activity					28 (standard ca	7,71	0,27	57,14			Liked fruit (food preference)	self-report questionnaire
																Liked vegetables & fruit (food pref	self-report questionnaire
																Total energy intake (kcal/day)	self-report; 24-h dietary recall
																Total vegetable intake (servings/da	self-report; 24-h dietary recall
																Whole fruit intake (servings/day)	self-report; 24-h dietary recall
																Fruit juice intake (servings/day)	self-report; 24-h dietary recall
																Total vegetable and fruit intake (se	self-report; 24-h dietary recall
																Sugar-sweetened beverage intake	self-report; 24-h dietary recall
																Sufficient vigorous physical activity	self-report questionnaire; Youth Risk Behavior Surveillance System criteria
																Sufficient moderate physical activi	self-report questionnaire; Youth Risk Behavior Surveillance System criteria
																Insufficient physical activity	self-report questionnaire; Youth Risk Behavior Surveillance System criteria
																Self concept: positive affect	self-report questionnaire
																Self concept: negative affect	self-report questionnaire
																Social support: caregiver support f	self-report questionnaire
																Social support: peer support for he	self-report questionnaire
																Social support: peer support for ur	self-report questionnaire
																BMI Z-score	objectively measured
Howe	2012	Pediatr Obes	USA	Controlled trial	Physical activity	11				26				9 weeks		BMI	objectively measured and
										Total groupmean age 3rd grade						weight (kg)	objectively measured
																weight status %	calculated based on CDC
																waist circumference	objectively measured
																total PA, min	measured with accelerometer
																light PA, min	measured with accelerometer
																moderate PA, min	measured with accelerometer
																MVPA, min	measured with accelerometer
																vigorous PA, min	measured with accelerometer
																very vigorous PA, min	measured with accelerometer
																physical activity energy expenditur	calculated with accelerometer
																sedentary behaviour, min	measured with accelerometer
																blood pressure	electronic monitor
																resting heart rate	electronic monitor
Hrafnkelsson	2014	Scand J Prim Health Ca	Iceland	RCT	Nutrition	128	7,4	0,3		139	7,3	0,3		2 years		Weight kg	objectively measured
					Physical activity											Height cm	objectively measured
																BMI kg/m	objectively measured and

																Percentage body fat	X-ray absorptiometry (DXA)
																Sum of five skinfolds (mm)	objectively measured
																Total cholesterol (mmol/L)	fasting blood sample analyses
																HDL cholesterol (mmol/L)	fasting blood sample analyses
																LDL-cholesterol (mmol/L)	fasting blood sample analyses
																Triglycerides (mmol/L)	fasting blood sample analyses
																Glucose (mmol/L)	fasting blood sample analyses
																HbA1C (%)	fasting blood sample analyses
																Serum-Insulin (mU/L)	fasting blood sample analyses
																resting SBP (mmHg)	meter
																resting DBP (mmHg)	meter
																Cardiorespiratory fitness (watts/kg	measured with maximal
																Fruit intake	records
																vegetable intake	
																fibre intake	
																MVPA, min	measured with acccelerometer
																PA during school, min	Teacher activity log book
Huang	2018	Health education & be	USA	RCT	Physical activity	24	9,8	0,8		30	10	0,7		2 months	1-2 wk before Bl, final	Child self-efficacy (to cycle to scho	Child-report self-administered survey
																Parent- child efficacy (to cycle to s	Parent-report self-administered survey
																Parent outcome expectations (to c	Parent-report self-administered survey
Huberty	2011	J Phys Act Health	USA	RCT	Physical activity	n=47, 49% girls				n=67, 54% girls				9 months		weight status %	calculated based on CDC
						ST group n=62, 60% girls				Total groupmean age grade 3-6							
						EQ group n=86, 52% girls										MVPA, % of time spent during rece	measured with accelerometer
Ickovics	2019	American journal of pr	USA	Cluster RCT	Nutrition	482	10,9	0,62		113	10,9	0,59		3 years	Bl, 1 year, 2 year, 3 yea	BMI percentile	Calculated based on Centers for Disease Control and Prevention age-adjusted and sex-adjusted growth charts
					Physical activity											BMI z-score	Calculated based on Centers for Disease Control and Prevention age-adjusted and sex-adjusted growth charts
																Nutritional behaviours - healthy fo	self-administered questionnaire
																Nutritional behaviours - unhealthy	self-administered questionnaire
																Nutritional behaviours - healthy dr	self-administered questionnaire
																Nutritional behaviours - consumpt	self-administered questionnaire
																Nutritional behaviours - fast food	self-administered questionnaire
																Proportion of students meeting PA	self-administered questionnaire
																Days per week with >60 min. of PA	self-administered questionnaire
Idamokoro, 2023	2022	International Journal o	South Africa	Quasi-experimental tw	Physical activity	57	7	0,7	52,6	36	7,4	0,7	52,8	9 weeks	Baseline, post-test afte	Body mass (kg)	
																Stature (cm)	
																Triceps skinfold (mm)	
																Subscapular skinfold (mm)	
																Waist circumference (cm)	
																Hip circumference (cm)	
																BMI (kg/m2)	
																Fat %	
																skinfolds)	
																criteria)	
Jakubowski	2018	MCN. The American jo	USA	RCT	Nutrition	36	2nd grade students (7 years)			35	2nd grade students (7 years)			8 weeks	Bl, 8 week	Healthy choices CATCH score (%)	self-report (CATCH survey)
																Nutritional knowledge score CATC	self-report (CATCH survey)
James	2004	BMJ	UK	RCT	Nutrition	325	8,7	0,9	48	319	8,7	0,9	51	1 year		BMI	objectively measured and
James	2007	BMJ												3 years		BMI z-score	calcuted using UK 1990 growth
																percentage children above the 91	calcuted using UK 1990 growth
																weight status %	calcuted using UK 1990 growth
																Total carbonated drinks, number o	self-report dietary questionnaire, over 2 weekdays and 1 weekend
																Carbonated drinks with sugar, nun	self-report dietary questionnaire, over 2 weekdays and 1 weekend
																Diet carbonated drinks, number of	self-report dietary questionnaire, over 2 weekdays and 1 weekend
																Carbonated drinks with sugar, nun	self-report dietary questionnaire, over 2 weekdays and 1 weekend
																Water, number of glasses in 3 days	self-report dietary questionnaire, over 2 weekdays and 1 weekend
Jansen	2011	Int J Pediatr Obes	The Netherlands	RCT	Physical activity	657 (group 1)	7,7	1	51	729	7,8	1	51	1 year		BMI	objectively measured and
					Nutrition	583 (group 2)	10,8	1	53	653	10,8	1	49			waist circumference (cm)	objectively measured
																weight status %	calculated based on IOTF cut-
																fitness	20 m Shuttle run
Jiang	2007	Child Care Health Dev	China	RCT	Nutrition	1029	8,4	1,4	48	1396	8,2	1,5	49	3 years		weight (kg)	objectively measured
					Physical activity											Height (cm)	objectively measured
																BMI	objectively measured and
																weight status %	calculated based on IOTF cut-
Johnston	2013	J Sch Health	USA	RCT	Physical activity	486	7,8	0,4	42	312	7,7	0,4	50	2 years		weight (kg)	objectively measured
					Nutrition											Height (cm)	objectively measured
																BMI	objectively measured and
																BMI z-score	calculated based on CDC growth data
																weight status %	calculated based on CDC growth data
																Grade Point Average (GPA)	Reported school grades
Jurić	2023	Prev Med Reports	Croatia	RCT	Physical activity	105	12,1	1,1		102	12,5	1,4		3 months	Bl, 3 months	Flamingo balance (n)	Eurofit test battery
																Plate tapping (sec)	Eurofit test battery
																Sit and reach (cm)	Eurofit test battery
																Standing long jump (cm)	Eurofit test battery
																Handgrip (kg)	Eurofit test battery
																Sit up in 30-sec (n)	Eurofit test battery
																Bent arm hang (sec)	Eurofit test battery
																10 x 5 shuttle run (sec)	Eurofit test battery
																20-m shuttle run (sec)	Eurofit test battery
																BMI (kg/m2)	objectively measured and
																Body fat (%)	Body composition analyser (Tanita)
Kain	2009	Public Health Nutr	Chile	Controlled trial	Nutrition	1759	10	2,3	49	671	9,9	2,2	39	2 years		BMI	objectively measured and
					Physical activity											BMI z-score	calculated based on CDC growth data
																weight status %	calculated based on CDC growth data
																waist circumference	objectively measured
																triceps skinfold thickness	objectively measured
																physical fitness	broad jump
																physical fitness	modified shuttle run test
																physical fitness	mile test
																physical fitness	20m shuttle run test
Kain	2014	J Obes	Chile	RCT	Nutrition	651	6,5	1,1	44	823	6,7	1,1	49	12 months		BMI z-score	calculated based on WHO
					Physical activity											weight status %	calculated based on WHO
																% snacks brought to school	
																% healthy food items brought to school	
																knowledge on healthy food	test of proportions
																Duration of physical education (PE	obervation of PE classes
																MVPA in PE, min	measured with pedometer

																% class time in MVPA	measured with pedometer
Kamin	2022	Nutrients	Slovenia	Quasi-experimental	Nutrition	(communication)	10-16-yr olds		49	164	10-16-yr olds		39	4-5 months	Pre and post intervent	BMI categories (underweight, norr	objectively measured; International Obesity Task Force criteria
					Environment	(environmental)	10-16-yr olds		45,7							BMI z-score	objectively measured
						97 (c and e)	10-16-yr olds		52,6							Drinking habits: water (mL/day)	self-report questionnaire (nr of drinking units e.g. one glass equivalent to 200mL)
																Drinking habits: beverages with su	self-report questionnaire (nr of drinking units e.g. one glass equivalent to 200mL)
																Drinking habits: juice (mL/day)	self-report questionnaire (nr of drinking units e.g. one glass equivalent to 200mL)
																Drinking habits: beverages with sw	self-report questionnaire (nr of drinking units e.g. one glass equivalent to 200mL)
																Drinking habits: beverages with su	self-report questionnaire (nr of drinking units e.g. one glass equivalent to 200mL)
																Drinking habits: sweet beverages (	self-report questionnaire (nr of drinking units e.g. one glass equivalent to 200mL)
Karczewski	2016	J Sch Health	USA	Quasi-experimental	Nutrition	88	8,5	1	49	37	8,3	0,6	70	6 months		weight status %	calculated based on CDC
					Physical activity											BMI percentiles	calculated based on CDC
Khan & Bell	2019	International Journal o	UK	Quasi-experimental	Nutrition	30	9,07	0,25	40	30	9,07	0,97	37,9	5 months	Pre and post intervent	Sedentary behaviour (minutes), du	objectively measured, accelerometer
					Physical activity											Light Physical Activity (minutes), d	objectively measured, accelerometer
																Moderate-to-vigorous Physical Act	objectively measured, accelerometer
																Daily vegetable consumption (freq	self-report questionnaire
																Daily fruit consumption (frequency	self-report questionnaire
																Attitude to eating vegetables	self-report questionnaire
																Attitude to eating fruits	self-report questionnaire
																Preferences of vegetable	self-report questionnaire
																Preferences of fruit	self-report questionnaire
																Knowledge of nutrition and plant s	self-report questionnaire
Kipping	2008	Arch Dis Child	UK	RCT	Nutrition	114	9,4	0,5	50	145	9,4	0,5	55	5 months		BMI	objectively measured and
					Physical activity											BMI z-score	calcuted using UK 1990 growth
																weight status %	calcuted using UK 1990 growth
																Walks/cycles to and from school	self-report questionnaire
																Screen viewing on weekdays (min)	self-report questionnaire
																Screen viewing on Saturdays (min)	self-report questionnaire
Klakk	2013	BMC Pediatr	Denmark	Quasi-experimental	Physical activity	351	9,3	0,9	54	281	9,4	0,9	47	2 years		BMI	objectively measured
																% total body fat	Dual Energy X ray
																weight status %	calculated based on IOTF
Klish	2012	J Pediatr Gastroenterol	USA	Controlled trial	Nutrition	779	8	2	46	510	7,9	2,1	49	9 months		weight status %	overweight P85< BMI <P95,
					Physical activity											overweight incidence	based on weight status
																obesity incidence	based on weight status
																overweight remission	based on weight status
																obesity remission	based on weight status
Kobel	2016	J Immigrant Minority H	Germany	RCT	Nutrition	318	7,2	0,7	53	207	7,1	0,7	49	1 year		BMI	objectively measured and
					Physical activity											BMI percentiles	calculated based on German
																weight status %	calculated based on German
																MVPA on >4 days/week >60 min/d	proxy-report questionnaire by
																Screen media >1 h/day	proxy-report questionnaire by
																consumption of sugar-sweetened l	proxy-report questionnaire by
																frequency of fruit and vegetable in	proxy-report questionnaire by
Kocken	2016	Nutrients	The Netherlands	RCT	Nutrition	615	9,2	0,6	52	497	9,1	0,6	51	2 years		BMI z-score	calculated based on IOTF cut-
					Physical activity											weight status %	calculated based on IOTF cut-
																hip-to-waist ratio z-score	objectively measured
																Total energy (kcal)	self-report questionnaire, 24h
																Total protein (en%) en% = percent	self-report questionnaire, 24h
																Total fat (en%)	self-report questionnaire, 24h
																Total saturated fat (en%)	self-report questionnaire, 24h
																Total carbohydrates (en%)	self-report questionnaire, 24h
																Total mono- and disaccharides (en	self-report questionnaire, 24h
																Fiber (g)	self-report questionnaire, 24h
																Breakfast intake Energy (en%)	self-report questionnaire, 24h
																Amount of fruits (g)	self-report questionnaire, 24h
																Amount of vegetables (g)	self-report questionnaire, 24h
																High fat or high energy snacks Ene	self-report questionnaire, 24h
																Total beverages Energy (en%)	self-report questionnaire, 24h
																Sugar sweetened beverages (en%)	self-report questionnaire, 24h
																Fruit juices (en%)	self-report questionnaire, 24h
																Other beverages (en%)	self-report questionnaire, 24h
																Daily breakfast	self-report questionnaire, 24h
																Daily fruit	self-report questionnaire, 24h
																Daily vegetables	self-report questionnaire, 24h
																Daily soft drinks	self-report questionnaire, 24h
																Daily savory snacks	self-report questionnaire, 24h
																Daily sweet snacks	self-report questionnaire, 24h
																Inactivity (min)	measured with accelerometer
																MVPA (min) (0.09 to 0.30)	measured with accelerometer
																Number of counts per minute	measured with accelerometer
																Norm active PA (>60min/d)	measured with accelerometer
																mean daily PA levels	measured with accelerometer
																screen time	measured with accelerometer
																knowledge	self-report questionnaire with
																Cognitive attitude toward the freq	self-report questionnaire with
																Affective attitude	self-report questionnaire with
																Perceived social norm of parents	self-report questionnaire with
																Self-efficacy	self-report questionnaire with
																Intention	self-report questionnaire with
Kriemler	2010	BMJ	Switzerland	RCT	Physical activity	297	9	0,4	52	205	9,1	0,5	51	1 year		BMI	objectively measured and
Meyer	2014	Plos One												3 years		waist circumference (cm)	objectively measured
																weight status %	calculated based on Swiss
																skinfold thickness, sum of triceps,	objectively measured
																PA, counts/min, over the whole da	measured with accelerometer
																PA, counts/min, time in school	measured with accelerometer
																PA, counts/min, time out of school	measured with accelerometer
																MVPA, min/day, over the whole da	measured with accelerometer
																MVPA,min/day, time in school	measured with accelerometer
																MVPA, min/day, time out of school	measured with accelerometer
																aerobic fitness	20 m shuttle run test
																Physical quality of life	self-report questionnaire,
																Psychological quality of life	self-report questionnaire,
																systolic blood pressure	measured with electronic
																diastolic blood pressure	measured with electronic
																Fasting glucose (mmol/L)	blood sample analysis

																HDL cholesterol (mmol/L)	blood sample analysis
																Triglycerides (mmol/L)	
																cardiovascular risk score	averaging the z scores of waist
Kühr	2019	Acta Paediatrica	Denmark	Controlled trial	Physical activity	676	8,4	1,4	55	502	8,5	1,5	49,7	5 years	Pre and yearly	BMI	objectively measured
																BMI SDS	objectively measured
																Overweight/obesity classification	objectively measured, obesity task force cut-offs
																Abdominal obesity	objectively measured, WC cut-offs
																Waist circumference	objectively measured
Koch	2019	Journal of Nutrition Education and Behavio		RCT	Physical activity	289 (curriculum)	10,5	0,53	44,3	301	10,8	0,63	48,5	1 school year	Pre and post intervent	Overweight/obesity prevalence (≥	objectively measured
					Nutrition	254 (wellness)	10,6	0,56	49,6							Frequency fruits and vegetables in	self-report questionnaires
					other	315 (both)	10,5	0,51	51,5							Fruit portion size	self-report questionnaires
																Vegetable portion size	self-report questionnaires
																Frequency soda, fruit drinks or ice	self-report questionnaires
																Frequency sports drinks or flavore	self-report questionnaires
																Portion size soda, fruit drinks or ic	self-report questionnaires
																Portion size sports drinks or flavor	self-report questionnaires
																Frequency processed packaged sn	self-report questionnaires
																Frequency chips	self-report questionnaires
																Frequency other salty snakcs	self-report questionnaires
																Frequency candy	self-report questionnaires
																Frequency donuts and pastries	self-report questionnaires
																Frequency cookies, brownies, pies	self-report questionnaires
																Portion sizze processed packaged	self-report questionnaires
																Freqyency fast food	self-report questionnaires
																Portion size fast food	self-report questionnaires
																Combo meal	self-report questionnaires
																Healthy options - salad, apples, fru	self-report questionnaires
																Healthy options - baked or grilled	self-report questionnaires
																Frequency physical actvity	self-report questionnaires
																Duration physical activity	self-report questionnaires
																Frequency television viewing	self-report questionnaires
																Frequency video game playing	self-report questionnaires
																Duration television viewing	self-report questionnaires
																Duration video game playing	self-report questionnaires
																Outcome expectation towards phy	self-report questionnaires
																Self-efficacy towards physical activ	self-report questionnaires
																Behavioral intention towards phys	self-report questionnaires
																Habit strenght towards physical ac	self-report questionnaires
																Outcome expectations towards TV	self-report questionnaires
																Self-efficacy towards TV viewing	self-report questionnaires
																Behavioral intention towards TV vi	self-report questionnaires
																Habit strenght towards TV viewing	self-report questionnaires
																Outcome expectations towards vic	self-report questionnaires
																Self-efficacy towards video game p	self-report questionnaires
																Behavioral intention towards videc	self-report questionnaires
																Habit strenght towards video gam	self-report questionnaires
																Outcome expectation towards frui	self-report questionnaires
																Self-efficacy toward fruits and veg	self-report questionnaires
																Behavior intention towards fruit ar	self-report questionnaires
																Habit strength of fruit and vegetab	self-report questionnaires
																Outcome expectation towards swe	self-report questionnaires
																Self-efficacy toward sweetened be	self-report questionnaires
																Behavior intention towards sweet	self-report questionnaires
																Habit strength of sweetened bever	self-report questionnaires
																Outcome expectation towards pro	self-report questionnaires
																Self-efficacy toward processed anc	self-report questionnaires
																Behavior intention towards proces	self-report questionnaires
																Habit strength of processed and pa	self-report questionnaires
																Outcome expectation towards fast	self-report questionnaires
																Self-efficacy toward fast food cons	self-report questionnaires
																Behavior intention towards fast fo	self-report questionnaires
																Habit strength of fast food consum	self-report questionnaires
																Goal-setting skills	self-report questionnaires
																Competence	self-report questionnaires
																Autonomous motivation	self-report questionnaires
Lambrick	2016	J Sports Sci		RCT	Physical activity	28	9,3	0,9	36	27	9,3	0,8	48	6 weeks		Height (cm)	objectively measured
																weight (kg)	objectively measured
																BMI	objectively measured and
																% body fat	objectively measured
																musclemass (kg)	objectively measured
																fat mass (kg)	objectively measured
																hip circumference (cm)	objectively measured
																waist circumference (cm)	objectively measured
																waist-to-hip ratio	objectively measured and
																Aerobic capacity = VO2 peak (ml /k	laboratory-based graded
																VO2 peak (ml /kg(FFM)/min)	GXT max
																maximal heart rate (beats/min)	GXT max
																minute ventilation = VE peak (l/mi	GXT max
																respiratory exchange ratio	GXT max
																Peak speed (km /hour)	GXT max
																Subjective perception of exertion (	Eston-Parfitt scale during GXT max
																VO2 (% of max GXT)	GXT submax
																VO2 (ml/kg/min)	GXT submax
																VO2 (ml/kg(FFM)/min)	GXT submax
																VE (l /min)	GXT submax
																Speed (km/hour)	GXT submax
																Speed (%)	GXT submax
Larson, J.N	2018	Clin Med Insights Pedia	USA	RCT	Physical activity	67	8 to 10			92	8 to 10			4 weeks	Bl, 4 wk (weekly during	Nr of steps (at school)	Objectively measured with accelerometers
					Nutrition					grade 4 and 5 students						Nr. of minutes in MVPA (at school)	Objectively measured with accelerometers
																Fruit and vegetable consumption (	Direct observation
Lazaar	2007	Acta Paediatr	France	RCT	Physical activity	197				228				6 months		weight (kg)	objectively measured
										Total group 50% girlsmean age 7.4 (0.8)						height (m)	objectively measured
																BMI	objectively measured and
																BMI z-score	reference data ?

																waist circumference (cm)	objectively measured
																sum of skinfold thickness (biceps, t	objectively measured and
																fat free mass	calculated from weight and
Lee	2016	Appl Nurs Res	Korea	Other	Nutrition	15			73	15			20	10 weeks		BMI	objectively measured and
					Physical activity					Total groupmean age grade 3-6						BMI percentiles	calculated based on CDC
					other											general self-esteem	self-report questionnaire (Coopersmith Self-Esteem Inventory)
																family self-esteem	self-report questionnaire (Coopersmith Self-Esteem Inventory)
																social self-esteem	self-report questionnaire (Coopersmith Self-Esteem Inventory)
																school self-esteem	self-report questionnaire (Coopersmith Self-Esteem Inventory)
Leuven	2018	Food Sci Nutr	The Netherlands	RCT	Nutrition	150	10 to 12			65	10 to 12			7 months	7 month	capability to identify vegetables	self-administered questionnaire
																Number of correctly identified veg	self-administered questionnaire
																preference for those vegetables	self-administered questionnaire
																the opinion regarding vegetables, ;	self-administered questionnaire
																Percentage of students that would	self-administered questionnaire
Li	2014	BMC Public Health	China	Controlled trial	Physical activity	388	10,2	2,3	47	533	10,6	2,2	47	12 weeks		BMI	objectively measured and
																weight status %	calculated based on Chinese
																incidence overweight/obesity	calculated based on Chinese
																remission overweight/obesity	calculated based on Chinese
																waist circumference, cm	objectively measured
																Skinfold thickness triceps, mm	objectively measured
																Skinfold thickness subscapular, mr	objectively measured
																Skinfold thickness abdominal, mm	objectively measured
																Total cholesterol, mmol/L	analysis of fasting blood sample
																Triglycerides, mmol/L	analysis of fasting blood sample
																HDL-C, mmol/L	analysis of fasting blood sample
																LDL-C, mmol/L	analysis of fasting blood sample
																Fasting glucose, mmol/L	analysis of fasting blood sample
																Duration of MVPA out of school, m	self-report questionnaire
																Duration of MVPA in school, min/c	self-report questionnaire
Li	2019	Plos medicine	China	RCT	Physical activity	832	6,2	0,4	44	809	6,1	0,4	47	12 months	Pre and post	BMI z-score	Objectively measured
					Nutrition											Prevalence overweight/obesity	Objectively measured, WHO 2007 BMI Growth Charts
																Waist circumference	Objectively measured
																Body fat percentage	Objectively measured
																Systolic blood pressure	Objectively measured
																Diastolic blood pressure	Objectively measured
																Consumption of fruit and vegetabl	Questionnaire, self-report
																Unhealthy snacks and sugar-sweet	Questionnaire, self-report
																Engagement in screen-based sede	Questionnaire, self-report
																Engagement in screen-based sede	Questionnaire, self-report
																Engaging in active sports, dance, o	Questionnaire, self-report
																Sedentary time	Objectively measured, accelerometer
																Sedentary time	Questionnaire, parent-reported (min/24 hours)
																MVPA time	Objectively measured, accelerometer
																MVPA time	Parent-reported (min/24 hours)
																Achieving at least 60 min of MVPA	Objectively measured, accelerometer
																Achieving at least 60 min of MVPA	Questionnaire, parent-report
																Health-related quality of life	Questionnaire, self-report; PedsQL total score & CHU9D utility score
																Social acceptance	Questionnaire, self-report (Kidscreen-52 bullying)
Lin	2019	Journal of Pediatric Nu	Taiwan	RCT	Nutrition	92	9	0,6	42	109	9,1	0,6	49	8 weeks	Pre and post	Weight	Objectively measured
					Physical activity											Height	Objectively measured
																Height z-score	Objectively measured
																BMI	Objectively measured
																BMI z-score	Objectively measured, CDC growth charts
																Physical activity (/active lifestyle) k	Questionnaire, self-report
																Physical activity behaviors score	Questionnaire, self-report
																Interests in NASA and space explo	Questionnaire, self-report
																Healthy diet knowledge score	Questionnaire, self-report
																Healthy diet behaviors score	Questionnaire, self-report
Liu	2007	Biomed Environ Sci	China	Controlled trial	Physical activity	328			54	425			51	9 weeks		height (cm)	objectively measured
Liu	2008	Obes Rev								Total group mean age 6-12 years						weight (kg)	objectively measured
																BMI	objectively measured and
																weight status %	calculated based on Chinese
																PA energy expenditure, kcal/day	self-report questionnaire or
																moderate PA energy expenditure,	self-report questionnaire or
																vigorous PA energy expenditure, k	self-report questionnaire or
																PA duration, h/day	self-report questionnaire or
																moderate PA duration, h/day	self-report questionnaire or
																vigorous PA duration, h/day	self-report questionnaire or
																PA energy expenditure, kcal per se	physical activity monitor
																Intensity of PA kcal/kg/h = metabo	physical activity monitor
Liu	2019	Childhood obesity (pri	China	Cluster RCT	Nutrition	930	9,15	0,75		959	9,06	0,58		12 months	Bl, 6 month 12 month	BMI	Objectively measured
					Physical activity											BMI z-score	Calculated using WHO references
																BMI status	according to chinese cutoffs
																Knowledge score (obesity related)	Self-report questionnaire
																No of days doing >1hr/day MVPA/	Self-report questionnaire
																Vegetable consumption	Self-report questionnaire
																Fruit consumption	Self-report questionnaire
																Meat consumtion	Self-report questionnaire
																SSB consumption	Self-report questionnaire
																Physical activity	Self-report questionnaire
																Screen time	Self-report questionnaire
Liu	2022	JAMA Pediatr	China	RCT	Physical activity	686	9.6	0.4	49.9	676	9.6	0.4	47.0	9 months	Bl, 4 and 9 months	BMI	Objectively measured
					Nutrition											BMI z-score	Calculated
																Body fat (%)	Objectively measured
																Waist circumference	Objectively measured
																Waist-to-hip ratio	Calculated
																Obesity (%)	Objectively measured, Chinese and WHO references
																Overweight/obesity (%)	Objectively measured, Chinese and WHO references
																In the action stage of behavioral change (%)	Self-report questionnaire
																Not drinking sugar sweetened beverages (%)	Self-report questionnaire
																Not eating fried food (%)	Self-report questionnaire

																Not eating Western fastfood (%)	Self-report questionnaire
																Screentime, <2hours/day (%)	Self-report questionnaire
																Daily physical activity with parents (days/week)	Self-report questionnaire
																Daily MVPA (days/week)	Self-report questionnaire
																Emotional overeating, score point	Self-report questionnaire
																Satiety responsiveness, score point	Self-report questionnaire
																Obesity related knowledge, score point	Self-report questionnaire
																Physical fitness	Objectively measured
																No. of rope jumps within 1 minute	Objectively measured
																Shuttle run (sec)	Objectively measured
																Long standing jump (cm)	Objectively measured
																No. of sit-ups within 1 minute	Objectively measured
																Diastolic blood pressure (mm Hg)	Objectively measured
																Systolic blood pressure (mm Hg)	Objectively measured
Llargues	2011	J Epidemiol Communit	Spain	RCT	Nutrition	272	6	0,3	46	237	6	0,3	46	2 years		BMI	objectively measured and
Mora	2015	Econ Hum Biol			Physical activity									6 years		weight status %	calculated based on IOTF cut-
Llargues	2017	Eur J Public Health												6 years		Eats fruit or juice daily	self-report questionnaire
																Eats a second fruit	self-report questionnaire
																Eats vegetables daily	self-report questionnaire
																Eats vegetables more than once da	self-report questionnaire
																Eats fish ≧2 times per week	self-report questionnaire
																Eats once or more weekly in a fast	self-report questionnaire
																Eats legumes ≧2 times perweek	self-report questionnaire
																Eats pasta or rice almost ≧5 per w	self-report questionnaire
																Eats pastries ≧ 3 times per week a	self-report questionnaire
																Eats nuts ≧2 times per week	self-report questionnaire
																Has milk or dairy products daily	self-report questionnaire
																Eats sweets several times a day	self-report questionnaire
																fizzy drinks ≧3 per week	self-report questionnaire
																Eats at school	self-report questionnaire
																Eats proper breakfast	self-report questionnaire
																Walks to school	self-report questionnaire
																PA after school	self-report questionnaire
																TV hours	self-report questionnaire
Llaurado, 2024	2018	Public Health	Spain	Parallel-cluster randomised controlled		375	9,2	0,5	48.8 (comple	327	9,2	0,5	51.8 (complet	10 months	baseline, 10, 16 month	fruit	
																MVPA	
																serving/day	
																(week + weekend)	
																once/week	
																day	
Lloyd	2012	BMJ Open	UK	RCT	Nutrition	80	9,7	0,3	50	122	9,7	0,3	50	2 years		BMI	objectively measured and
					Physical activity											BMI z-score	calculcated according to
																% Body fat	bioelectrical impedance
																% Body fat z-score	calculcated according to
																Waist circumference (cm)	objectively measured
																Waist circumference z-score	calculcated according to
																Weight status %	calculated based on IOTF cut-off
																Consumption of energy-dense sna	self-report questionnaire
																Consumption of healthy snacks	self-report questionnaire
																Positive food markers	self-report questionnaire
																Negative food markers	self-report questionnaire
																Duration of TV viewing (hours/day	self-report questionnaire
																Sedentary activities (hours/day)	self-report questionnaire
																MVPA (mins/day)	measured with accelerometer
Lloyd	2018	The Lancet Child and A	UK	Cluster RCT	Nutrition	676	9,8	0,3		648	9,7	0,3		3 school terms, i.e. appr	Bl, 12 month, 18 mont	Change in BMI standard deviation	Calculated using LMS method for normalised growth standards
					Other											Weight status (obesity, overweigh	based on Cole criteria
					Physical activity											waist circumference SDS (standarc	Objectively measured
																percentage body fat SDS (standarc	Objectively measured
																Weekly acceleration	Objectively measured with accelerometers
																Daily total PA, min	Objectively measured with accelerometers
																Daily light PA, min	Objectively measured with accelerometers
																Daily moderate PA, min	Objectively measured with accelerometers
																Daily moderate to vigorous PA, mi	Objectively measured with accelerometers
																Daily vigorous PA, min	Objectively measured with accelerometers
																Daily sedentary, min	Objectively measured with accelerometers
																daily energy dense snacs intake (al	Self-report questionnaire
																Daily healthy snacks intake (all day	Self-report questionnaire
																Daily positive food markers intake	Self-report questionnaire
																Daily negative food markers intake	Self-report questionnaire
Lloyd	2017 (1)	BMC public health	UK	RCT	Nutrition	676	9,8	0,3		648	9,7	0,3		1 year	24 month	BMI	Objectively measured
Lloyd	2017 (2)	Trials	UK		Other											BMI sds	Objectively measured and calculated using UK 1990 BMI reference curves for children
																waist circumference	Objectively measured
																waist circumference SDS (standarc	Objectively measured and calculated using UK 1990 waist circumference reference curves for children
																Percentage of body fat	Objectively measured
																Percentage of body fat SDS after e	Objectively measured and calculated using UK 1990 body fat percentage reference curves for children
																Percentage of body fat SDS	Objectively measured and calculated using UK 1990 body fat percentage reference curves for children
																Energy dense snacks (weekday, we	Self-report questionnaire
																Healthy snacks (weekday, weekend	Self-report questionnaire
																Positive markers (weekday, weeke	Self-report questionnaire
																Negative markers (weekday, week	Self-report questionnaire
																Physical activity - sedentary	Objectively measured with accelerometers
																PA - light	Objectively measured with accelerometers
																PA - moderate	Objectively measured with accelerometers
																PA - vigorous	Objectively measured with accelerometers
																PA - MVPA	Objectively measured with accelerometers
																Mean of mg value	Objectively measured with accelerometers
López Sánchez,	2017	Science and Sports	Spain	Cluster RCT	Physical activity	60		0,49		60	females (n=54) 1	0,48		12 weeks	Bl, 12 week	Body weight	Objectively measured with Tanita bioelectrical impedance analysis

											males (n=66) 10,36					BMI	Objectively measured and calculated
											total group					Fat free mass	Objectively measured with Tanita bioelectrical impedance analysis (BIA)
																Fat free mass percentage	Objectively measured with Tanita bioelectrical impedance analysis (BIA)
																Fat-free mass index	Objectively measured with Tanita bioelectrical impedance analysis (BIA)
																Fat mass	Objectively measured with Tanita bioelectrical impedance analysis (BIA)
																Fat mass percentage	Objectively measured with Tanita bioelectrical impedance analysis (BIA)
																fat mass index	Objectively measured with Tanita bioelectrical impedance analysis (BIA)
																Total body water (liter)	Calculated from BIA data
Long	2020	Preventive Medicine	US	Controlled trial	Physical activity	2730	8,7	not reported	49	3152	8,7	not reported	48	Fall 2013 to spring 2015		BMI	Objectively measured
					Nutrition									1 year and ~9 months?		BMI z-score	Objectively measured
																% students engaging in MVPA	SOPLAY observation tool
																% students engaging in sedentary l	SOPLAY observation tool
																Step counts (pedometer)	Objectively measured (pedometer)
																Sports drink/sweetened fruit punc	Self-report questionnaire
																Regular soda	Self-report questionnaire
																Sports drink/sweetened fruit punc	Self-report questionnaire
																Battered or fried chicken, other fri	Self-report questionnaire
																Grain-based desserts, cookies, cak	Self-report questionnaire
Long	2022	BMC Medicine	South Africa, Tanzar	RCT	Nutrition	PA = 347	8,8	[8,7-8,9] (95% CI)	51	335	8.0	[7,8-8,1]	46	Not clear; 9 months (firs	Bl, after 9 month	Fat mass	Objectively measured
Long	2024	Am J Prev Med			Physical activity	A + MMNS = 297	8,3	[8.2-8.5]	49						29 months	Fat free mass	Objectively measured
						MMNS = 325	8,2	[8.0-8.3]	46							Truncal fat mass	Objectively measured
																Truncal fat free mass	Objectively measured
Lynch	2016	J Prim Care Community	USA	RCT	Nutrition	29			55	37			56	4 months		BMI	objectively measured and
					Physical activity					Total group median age 8 (7-9) yr						BMI percentiles	No reference data reported
																Eating more fruits or vegetables pe	proxy-report questionnaire (Healthy Habits Survey), by parents
																eating less takeout or fast food	proxy-report questionnaire (Healthy Habits Survey), by parents
																eating breakfast more often	proxy-report questionnaire (Healthy Habits Survey), by parents
																eating at the dinner table more oft	proxy-report questionnaire (Healthy Habits Survey), by parents
																Drinking less soda, punch, fruit dri	proxy-report questionnaire (Healthy Habits Survey), by parents
																drinking more water	proxy-report questionnaire (Healthy Habits Survey), by parents
																drinking more non-fat or reduced f	proxy-report questionnaire (Healthy Habits Survey), by parents
																drinking less whole milk	proxy-report questionnaire (Healthy Habits Survey), by parents
																Less time watching screens	proxy-report questionnaire (Healthy Habits Survey), by parents
																increasing the amount of sleep pe	proxy-report questionnaire (Healthy Habits Survey), by parents
																removing the TV from the bedroor	proxy-report questionnaire (Healthy Habits Survey), by parents
																more time in active play	proxy-report questionnaire (Healthy Habits Survey), by parents
																number of steps per day	measured with pedometer
Lyu	2022	Nutirients	China	RCT	Physical activity	605	9,6	0,5	49,8	571	9,6	0,5	48,2	9 months	Bl, 9 months	BMI (kg/m2)	objecctively measured
					Nutrition											BMI z-score	objectively measured and calculated based on WHO standards
					Environment											Waist circumference (cm)	objecctively measured
					other											Body fat (%)	objecctively measured
																Cereals, under-intake	Food frequency questionnaire
																Vegetables, under-intake	Food frequency questionnaire
																Fruits, under-intake	Food frequency questionnaire
																Milk and dairy products, under-int	Food frequency questionnaire
																Soybean and soybean products, ur	Food frequency questionnaire
																Red meat, poultry and game, unde	Food frequency questionnaire
																Fish and shrimp, under-intake	Food frequency questionnaire
																Eggs, under-intake	Food frequency questionnaire
																Water intake	Food frequency questionnaire
																Cereals, over-intake	Food frequency questionnaire
																Read meat, poultry and game, ove	Food frequency questionnaire
																Eggs, over-intake	Food frequency questionnaire
																Sugar sweetened beverages, over-	Food frequency questionnaire
																Unhealthy snacks, over-intake	Food frequency questionnaire
																Inadequate dietary diversity	Food frequency questionnaire
																Higher bound score (dietary qualit	self-report questionnaire
																Lower bound score (dietary quality	self-report questionnaire
																Diet quality distance	self-report questionnaire
Madsen	2015	Child Obes	USA	RCT	Nutrition	583				296				2 years		BMI z-score	objectively measured and
					Physical activity					Total groupmean age grade 3-5						MVPA, min during school	measured with accelerometer
																sedentary behaviour, min during sc	measured with accelerometer
																cardiorespiratory fitness	1-mile run
																fruit consumption (cups)	estimation based on digital
																vegetable consumption (cups)	estimation based on digital
																consumption of milk	self-report questionnaire,
																consumption of sweetened bevera	self-report questionnaire,
																eating at restaurants	self-report questionnaire,
																consumption of salty or sweet sna	self-report questionnaire,
																Dietary knowledge	self-report questionnaire
Magnusson	2012	Health Educ Res	Iceland	RCT	Physical activity	128	7,3	0,3	51	138	7,4	0,3	60	2 years		BMI	objectively measured
					Nutrition											Skinfolds (sum of 5)	objectively measured
																Waist circumference (cm)	objectively measured
																Lean mass (kg)	dual energy x-ray scan (DEXA)
																Fat mass (%)	dual energy x-ray scan (DEXA)
																Fat mass, z-score	calculated
																Cardiorespiratory fitness (W/kg)	ergometer test
																Cardiorespiratory fitness , z-score	calculated
																Maximum heart beats/min	objectively measured
Manley	2014	J Pediatr Nurs	USA	RCT	Physical activity	55	11,4	0,6	51	61	11,7	0.8	51	13 weeks		relative BMI	calculated based on CDC
					other											physical activity, steps	measured with pedometer
																aerobic fitness	1-mile walk test
																self-efficacy for PA	self-report questionnaire
Marcus	2009	Int J Obes (Lond)	Sweden	RCT	Nutrition	1670	7,4	1,3	49	1465	7,5	1,3	49	4 years		BMI z-score	calculated based on IOTF cut-off
					Physical activity											weight status (%)	calculated based on IOTF cut-off
																PA, counts/min during school time	measured with accelerometer
																PA, counts/min during after school	measured with accelerometer
																PA, counts/min during evening tim	measured with accelerometer
																Total PA, counts/min	measured with accelerometer
																Consumption of fruit and vegetabl	proxy-report questonnaire, by
																Consumption of sweet drinks	proxy-report questonnaire, by
																Consumption of ice cream, cookies	proxy-report questonnaire, by
																Consumption of crisps/nuts	proxy-report questonnaire, by
																Consumption of fast food	proxy-report questonnaire, by

																Consumption of bread	proxy-report questonnaire, by
																Consumption of cereals	proxy-report questonnaire, by
																Consumption of dairy products	proxy-report questonnaire, by
																Breakfast eating habits	proxy-report questonnaire, by
																Dinner eating habits	proxy-report questonnaire, by
																Eating attitudes	self-report questionnaire,
Marsigliante, 2022	2017/18	Frontiers in Nutrition	Italy	Randomised controllec	Diet	198	9,1	0,5	52,5	200	9,1	0,7	50	6 months	Baseline, 6 month FU	BMI percentile	
																Disease Control and Prevention	
																Waist circumference (cm)	
																Waist-to-height ratio	
																Breakfasting, morning	
																Snacking, morning	
																Snacking, afternoon	
																Intake of fruit, morning	
																Intake of sweets, morning	
																Intake of chips, morning	
																Intake of cold cuts, morning	
																Intake of fruit, afternoon	
																Intake of sweets, afternoon	
																Intake of chips, afternoon	
																Intake of cold cuts, afternoon	
																Intake of fruits/day	
																Intake of vegetables/day	
																Intake of sweets/day	
																Intake of sweetened drinks	
Marsigliante	2023	International Journal o	Italy	Randomised controllec	Physical activity	157	9,9	0,7	54,1	153	9,9	0,5	56,2	6 months	Baseline, 6 month FU	BMI (kg/m2)	
																BMI percentile	
																Waist circumference (cm)	
																using waist circumference)	
																Waist-to-height ratio	
																Disease Control and Prevention	
																Standing long jump (m)	
																endurance)	
																Sit and reach test (cm)	
																number of responses; CP, nr of	
																(Warwick-Edinburgh)	
Martınez Vizcaıno	2008	Int J Obes (Lond)	Spain	RCT	Physical activity	513	9,4	0,7	49	606	9,5	0,7	51	9 months		Weight status %	calculated based on IOTF
Salcedo Aguilar	2010	J Pediatr												2 years		BMI	objectively measured and
																Triceps skinfold thickness (mm)	objectively measured
																% Body fat	Bioelectrical impedance
																resting SBP (mm Hg)	automatic sphygmomanometer
																resting DBP (mm Hg)	automatic sphygmomanometer
																Total cholesterol (mg/dl)	fasting blood sample analysis
																Apolipoprotein B (mg/dl)	fasting blood sample analysis
																Apolipoprotein A-I (mg/dl)	fasting blood sample analysis
																Triglycerides (mg/dl)	fasting blood sample analysis
Martínez-Vizcaíno	2014	Int J Behav Nutr Phys A	Spain	RCT	Physical activity	420	9,4	0,7	55	492	9,5	0,7	49	1 schoolyear		BMI	objectively measured and
																weight status (%)	calculated based on IOTF cut-off
																Triceps skinfold thickness	objectively measured
																Body fat %	bioimpedance analysis
																Fat-free mass, kg	bioimpedance analysis
																Waist circumference, cm	objectively measured
																LDL-chol, mg/dl	fasting blood sample analyses
																Triglycerides/HDL-chol	fasting blood sample analyses
																Insulin, µU/mL	fasting blood sample analyses
																C-reactive protein	fasting blood sample analyses
																Mean arterial pressure	calculated based on DBP and SBP, measured with electronic monitor
																MVPA, min/day	measured with accelerometer
																Sedentary time (min/day)	measured with accelerometer
Martinez-Vizcaino	2022	Scand J Med Sci Sports	Spain	RCT	Physical activity	248	10.0	0.7	51.6	239	10.1	0.7	52.7	7 months	Bl and 8 months	Waist circumference (cm)	Objectively measured
																Ratio waist circumference (cm) and height (cm)	Calculated
																Fat mass (%)	Objectively measured
																Fat-free mass (kg)	Objectively measured
																BMI (kg/m2)	Objectively measured
																BMI z-score	Calculated
																Underweight (%)	Calculated
																Overweight/obesity (%)	Calculated
																Systolic blood pressure (mm Hg)	Objectively measured
																Diastolic blood pressure (mm Hg)	Objectively measured
																Pulse pressure (mm Hg)	Calculated
																Mean arterial pressure (mm Hg)	Calculated
																Total cholesterol (mg/dL)	Objectively measured
																HDL cholesterol (mg/dL)	Objectively measured
																LDL cholesterol (mg/dL)	Objectively measured
																Triglyceride (mg/dL)	Objectively measured
																Triglyceride/HDL cholesterol	Calculated
																non-HDL cholesterol (mg/dL)	Objectively measured
																Glucose (mg/dL)	Objectively measured
																Log_insulin (µU/ml)	Objectively measured
																HbA1c (%)	Objectively measured
																Metabolic syndrome index	Calculated
																C-reactive proteine (mg/L)	Objectively measured
																Cardio respiratory fitness (stages)	Objectively measured
																Cardio respiratory fitness (VO 2 max, ml/Kg/min)	Objectively measured
																Cardio respiratory fitness (laps)	Objectively measured
																Velocity/agility (sec)	Objectively measured
																Flexibility (cm)	Objectively measured

																Upper body muscular strength (kg dynanometer/kg body weight)	Calculated
																Handgrip strength (kg)	Objectively measured
																Standing long jump test (cm)	Objectively measured
																Lower body muscular strength (cm long jump/kg body weight)	Calculated
McMurray	2002	J Adolesc Health	USA	RCT	Physical activity	(Education only)	12,1	0,1	55	247	12,1	0,1	55	8 weeks		Weight (kg)	objectively measured
						319 (Exercise only)	12,3	0,1	55							Height (cm)	objectively measured
						308 (Education & Exercise)	12,3	0,1	55							BMI	objectively measured and
																Skinfolds thickness, sum of triceps,	objectively measured
																resting SBP (mmHg)	measured with sphygmomano-
																resting DBP (mmHg)	measured with sphygmomano-
																Aerobic power VO2max(mL/kg/mi	submaximal cycle ergometry
McWhanell	2008	Int J Sports Med	UK	Controlled trial	Physical activity	5 (Lifestyle pass)	11	0,3	59	30	11	0,3	59	9 weeks		weight (kg)	objectively measured
						16 (Structured exercise)	11,1	0,3	59							height (m)	objectively measured
																BMI	objectively measured and
																waist circumference (cm)	objectively measured
																% body fat	dual-energy X-ray
																% trunk fat	DEXA
																Fat mass index (g/m2)	DEXA
																Lean mass index (g/m2)	DEXA
																Bone Mineral Density (BMD) Total	DEXA
																Bone Mineral Content (BMC) Total	DEXA
																BMD Femoral Neck (g/cm2)	DEXA
																BMC Femoral Neck (g)	DEXA
																BMD Lumbar Spine (g/cm2)	DEXA
																BMC Lumbar Spine (g)	DEXA
Menor-Rodriguez, 2022	2014/15	Children	Spain	Semi-experimental unc	Nutrition	479	8,7	1,5	53,9	No contol	No contol	No contol	No contol	6 months	Baseline, 4mo, 7 mo FU	Obesity Task Force cut offs (%)	
																Completing 5 daily meals (%)	
																Eating breakfast (%)	
																Eating mid-morning snack (%)	
																Eating lunch (%)	
																Eating afternoon snack (%)	
																Eating supper (%)	
																(%)	
																(%)	
																breakfast (%)	
																breakfast (%)	
																at breakfast (%)	
																at breakfast (%)	
																at breakfast (%)	
																(%)	
																meats, for lunch (%)	
																pulses, for lunch (%)	
																as afternoon snack (%)	
																afternoon snack (%)	
																afternoon snack (%)	
																afternoon snack (%)	
																pulses, as afternoon snack (%)	
																for supper (%)	
																(%)	
																(%)	
																supper (%)	
																pulses, for supper (%)	
Merrotsy	2019	Public health nutrition	Ireland	Controlled trial	Nutrition	6-yr-olds: 49	6,1	0,3	52	(characteristics for INT + CON; not separately reported)				2 schoolyears (Oct-14 -	Pre and post	BMI	Objectively measured
					Physical activity	10-yr-olds: 52	9,9	0,4	48	(characteristics for INT + CON; not separately reported)						Systolic blood pressure	Objectively measured
																Diastolic blood pressure	Objectively measured
																Waist-to-height ratio	Objectively measured
																Run time (measure of cardiorespir	Objectively measured; 550m walk/run test
																Nutritional knowledge (overall sco	Self-report questionnaire
																Fibre intake	Self-report questionnaire (4-d food diary)
																Na intake	Self-report questionnaire; micronutrient intake
																Ca intake	Self-report questionnaire
																Fe intake	Self-report questionnaire
																Fat (% of energy) intake	Self-report questionnaire; macronutrient intake
																Saturated fat (% of energy) intake	Self-report questionnaire
																Sugar (% of energy) intake	Self-report questionnaire
																Protein (% of energy) intake	Self-report questionnaire
																Total servings of fruits and vegetal	Self-report questionnaire
																Number of unhealthy snacks /day	Self-report questionnaire
Morais	2021	European Journal of Public Health	Brazil	RCT	Physical activity	Pgroup (508)	11,55	1,48	47,6	1196	11,53	1,5	47	5 months	Baseline (Feb/Mar) and FU (Nov/Dec)	Physical activity (mins/week)	Self-report questionnaire
					Nutrition Environment	PEGroup (807)	11,57	1,4	48,3							Achieving 150mins plus of PA	Self-report questionnaire
Morgado	2023	Front Pediatr	Portugal	RCT	Physical activity	FG: 23	8,6	0,8	23	8,6	0,7			12 weeks	Bl, 13 weeks	Height	Objectively measured
					Nutrition	NFG: 21	9,4	0,3								Weight	Objectively measured
																BMI	Objectively measured
																BMI z-score	Objectively measured and calculated based on Cole criteria
																WC (cm)	Objectively measured
																Waist-to-height ratio	objectively measured and calculated
																Body fat (%)	Bioimpedance measure
																Muscle mass (kg)	Bioimpedance measure
																Fat-free mass (kg)	Bioimpedance measure
																Systolic blood pressure (mmHg)	Objectively measured
																Diastolic blood pressure (mmHg)	Objectively measured
																resting HR (beats/min)	Objectively measured
																Horizontal jump length (cm)	Objectively measured
																20 meter sprint (s)	Objectively measured
																Shuttle run (m)	Objectively measured
																Sports club active (%)	
																MVPA (min/day)	Accelerometer-based
																Incex FAI (body image)	Collins' child figure drawing scale

																Physical health	PedsQL, self-report
																Psychosocial health	PedsQL, self-report
																Quality of Life	PedsQL, self-report
																NKO score	PedsQL, self-report
																Energy (kcal)	24-hour recall
																Energy/kg	24-hour recall
																Protein (g)	24-hour recall
																Carbohydrate (g)	24-hour recall
																Fiber (g)	24-hour recall
																Sugar (g)	24-hour recall
																Fat (g)	24-hour recall
																Saturated fat (g)	24-hour recall
																Sodium (mg)	24-hour recall
																Fruit intake, daily, <6 times/week	24-hour recall
																100% juice intake, daily, <6 times/	24-hour recall
																Soup intake, daily, <6 times/week	24-hour recall
																Vegetable intake, daily, <6 times/v	24-hour recall
																Cereal intake, daily, <6 times/weel	24-hour recall
																Cookie intake, daily, <6 times/wee	24-hour recall
																Fish intake, daily, <6 times/week	24-hour recall
																Meat intake, daily, <6 times/week	24-hour recall
																Eggs intake, daily, <6 times/week	24-hour recall
																Milk intake, daily, <6 times/week	24-hour recall
																Cheese intake, daily, <6 times/wee	24-hour recall
																Yogurt intake, daily, <6 times/wee	24-hour recall
																Sweet snacks intake, daily, <6 time	24-hour recall
																Salty snacks intake, daily, <6 times	24-hour recall
																Soft drinks intake, daily, <6 times/	24-hour recall
Muller	2019	International journal o	South Africa	RCT	Physical activity	300	10,1	0,9	50	446	9,9	1	50	20 weeks	Pre and post	Cardiorespiratory fitness: shuttle r	Objectively measured
					Other (hygiene)											Cardiorespiratory fitness: VO2max	Objectively measured
																BMI z-score	Objectively measured and calculated using WHO guidelines
																Skinfold thickness	Objectively measured; triceps/subscapular
																Physical activity score (PA in the pe	Self-report questionnaire
																Prevalence of overweight and obe	Objectively measured; WHO guidelines
																Prevalence of stunting	Objectively measured, height-for-age Z-score
																Prevalence of anaemia	Objectively measured; Hb concentration
																Prevalence of infection: soil transn	Objectively measured; stool sample
																Prevalence of infection: intestinal	Objectively measured; stool sample
Muckelbauer	2009	Obes Facts	Germany	RCT	Nutrition	1641	8,3	0,7	50	1309	8,3	0,8	50	1 school year		weight status (%)	calculated based on IOTF cut-off
Muckelbauer	2009	Pediatrics														BMI	objectively measured and calculated
																BMI z-score	calculated based on IOTF cut-off
																incidence rate	calculated based on IOTF cut-off
																admission rate	calculated based on IOTF cut-off
																Mean consumption of water, glass	self-report questionnaire,
																mean consumption of soft drinks/j	self-report questionnaire,
Müller	2001	Int J Obes (Lond)	Germany	RCT	Nutrition	344	6,3	5,9-6,5	51	1420	6.3	6.0-6.5	49	4 years		height (m)	objectively measured
Danielzik	2007	Acta Pediatr			Physical activity											weight (kg)	objectively measured
Plachta-Danielzik	2007	Obesity														BMI	objectively measured
																waist circumference	objectively measured,
																Skinfold thickness, triceps	objectively measured,
																weight status %	calculated based on BMI, WC
																fat mass	bioelectrical impedance
																incidence of overweight	calculated based on BMI, WC
																remission of overweight	calculated based on BMI, WC
																Healthy eating index %	questionnaire
																Nutrition Knowledge	self-report questionnaire
																Daily fruit and vegetable consump	self-report questionnaire
																Daily intake of low fat food	self-report questionnaire
																physical activity %	questionnaire
																Membership of sport club	self-report questionnaire
																media time %	questionnaire
																TV watching (hours/day)	self-report questionnaire
																lifesyle %	questionnaire
Muros	2013	Pediatr Exerc Sci	Spain	RCT	Nutrition	32 (at follow-up)			40	32 (at follow-up)			66	7 weeks		Weight (kg)	objectively measured
					Physical activity					Total groupmean age 10.6 (0.4)						BMI (kg/m2)	objectively measured
																% body fat	calculated
																Skinfold thickness sum of 6: tricep	objectively measured
																Skinfold thickness sum of 8: tricep	objectively measured
																resting SBP (mmHg)	measured with electronic
																resting DBP (mmHg)	measured with electronic
																Total cholesterol (mg/dl)	fasting blood sample analyses
																HDL-chol (mg/dl)	fasting blood sample analyses
																LDL-chol (mg/dl)	fasting blood sample analyses
																Triglycerides (mg/dl)	fasting blood sample analyses
																Energy (Kcal)	self-report questionnaire
																Proteins (g)	self-report questionnaire
																Carbohydrates (g)	self-report questionnaire
																Fats (g)	self-report questionnaire
																Cholesterol (mg)	self-report questionnaire
																Dietary Habits (points)	self-report questionnaire
																Cereal/similar	self-report questionnaire
																Breakfast (% consumption per day	self-report questionnaire
																Pasta or Rice +3 times/wk	self-report questionnaire
																Physical Activity (points)	self-report questionnaire
																Physical exercise (hours/day)	self-report questionnaire
																Television watching (hours/day)	self-report questionnaire
																maximal oxygen uptake (VO2max)	20m shuttle run test
Narayanan	2019	Preventing Chronic Dis	USA	Quasi-experimental	Physical activity	School 1: 51	10,1	0,3	52	School 1: 35	10,1	0,2	60	4 years		BMI percentile	Objectively measured
					Nutrition	School 2: 80	10,2	0,4	52	School 3: 85	10,3	0,5	48			Systolic blood pressure	Objectively measured
																Diastolic blood pressure	Objectively measured
																Physcial activity (sports participati	Self-report questionnaire
																Water intake	Self-report questionnaire
																Sweetened beverage intake	Self-report questionnaire

Ng	2022	Human nutrition & me	Malaysia	RCT	Nutrition	43	18 10-yr-olds; 25 11-yr-olds		65	43	14 10-yr-olds; 29 11-yr-olds		56	12 weeks	Pre and post	BMI for age (z-score)	Objectively measured and calculated
																Waist circumference	Objectively measured
																Body fat (%)	Objectively measured
																Consumption of: whole grains	Self-report questionnaire
																Consumption of: refined grains	Self-report questionnaire
																Consumption of: vegetables	Self-report questionnaire
																Consumption of: fruits	Self-report questionnaire
																Consumption of: meat/poultry	Self-report questionnaire
																Consumption of: legumes	Self-report questionnaire
																Consumption of: fish	Self-report questionnaire
																Consumption of: dairy	Self-report questionnaire
																Consumption of: processed foods	Self-report questionnaire
																Consumption of: sweetened bever	Self-report questionnaire
Nqweniso	2021	South African Journal c	South Africa	RCT	Nutrition	451	8-11 year olds		48	447	8-11 year olds		49	10 weeks	Pre and post	BMI	Objectively measured
					Physical activity											BMI for age (z-score)	Objectively measured
																Body fat (%)	Objectively measured; skinfolds, slaughter equation
Nickel	2021	Maternal & Child Nutri	Canada	RCT	Physical activity	157	60,5% ≤ 8 years		48,2	154	89% ≤ 8 years		48,7	21 weeks	Pre and post intervent	BMI z-score	objectively measured, CDC growth charts
					Nutrition		39,5% > 8 years				11% > 8 years					Waist circumference	objectively measured
					other		(6-12 yr-olds)				(6-12 yr-olds)					Dietary intake, frequency of eating	self-report questionnaire
																Healthy living knowledge (whether	self-report questionnaire
																Self-efficacy	self-report questionnaire
Nogueira	2014	Bone	Australia	Controlled trial	Physical activity	76 (girls group)	10,5	0,6	100	75	10,7	0,6	100	9 months		Weight (kg)	objectively measured
Nogueira	2015	Pediatr Exerc Sci				104 (boys	10,5	0,5	0	68	10,7	0,6	0			Lean mass (kg)	DEXA
																Fat mass (kg)	DEXA
																Standing height (m)	objectively measured
																Sitting height (m)	objectively measured
																Waist circumference (cm)	objectively measured
																Maturity	Years to age of peak height
																Muscle power	Vertical jump (cm)
																aerobic capacity	20-metre shuttle-run test
																max oxygen consumption (VO2 ma	calculated based on velocity and
																Resting heart rate (beats/min)	objectively measured
																resting BP (mm Hg)	measured with sphygmomanometer
																Stiffness index (%)	measured with quantitative ultrasonometry
																Broadbent ultrasound attenuation	ultrasonometry
																Whole body bone mineral content	dual-energy X-ray
																Whole body bonemineral density (	DXA
																lumbar spine bone mineral conten	DXA
																lumbar spine bonemineral density	DXA
																lumbar spine index of bone structu	DXA
																femoral neck bone mineral conten	DXA
																femoral neck bonemineral density	DXA
																Tibial total content 4% (mg)	peripheral Quantitative
																Tibial trabecular density 4% (mg/c	pQCT
																Tibial cortical density 38% (mg/cm	pQCT
																Radial total content 4% (mg)	pQCT
																Radial trabecular density 4% (mg/c	pQCT
																Radial cortical density 38% (mg/cm	pQCT
Nogueira	2017	Pediatric exercise scier	Australia	RCT	Physical activity	155	12,2	1		85	12,3	0,6		9 months	Bl, 9 month, 21 month	Weight	Objectively measured
																Height	Objectively measured
																BMI	Objectively measured
																Sitting height	Objectively measured
																Biological maturity (APHV)	Calculated using standard algorithm
																Waist circumference	Objectively measured
																Resting heart rate	Objectively measured
																Blood pressure	Objectively measured
																Vertical jump	Objectively measured
																Estimated VO2max	Objectively measured via 20-m shuttle run test
																BUA (broad ultrasound attenuatio	Objectively measured via quantitative ultrasonometry
																SI% (stiffness index percentage)	Objectively measured via quantitative ultrasonometry
Norman	2024	Prevention Science	Sweden	RCT	Physical activity	152	6,3	0,3	49,3	193	6,3	0,3	50,8	7 months	Bl, 7 months	Healthy food	Parent-report
					Nutrition											Unhealhty food	Parent-report
					other											Sweet beverages	Parent-report
																MVPA, weekdays	Accelerometr
																MVPA, weekend days	Accelerometr
																MVPA, non-school time	Accelerometr
																Sedentary, weekdays	Accelerometr
																Sedentary, weekend days	Accelerometr
																Sedentary, non-school time	Accelerometr
																BMI sds	Objectively measured and calculated using Swedisch reference standard
																Waist circumference (cm)	Objectively measured
Olvera	2024	Ann Nutr Metab	Mexico	RCT	Nutrition	142	9,6	0,6	52,7	165	9,7	0,6	58,3	6 months	Bl, 6 months	Water	24-hour recall
																Water flavored with fruits and add	24-hour recall
																Flavored milk	24-hour recall
																Fruit juice and soft drinks	24-hour recall
																All sugar sweetened beverages	24-hour recall
																Energy (kcal)	24-hour recall
Paulsamy, 2021	Not repor	Healthcare	India	Randomised controllec	Nutrition	520	13.2 (not repo	Not reported	55,6	485	13.2 (not reporte	Not reported	57,9	6 months	Baseline, post1 (3 mo),	BMI status, WHO cut offs	
					Physical activity											Systolic blood pressure (mmHg)	
																Diastolic blood pressure (mmHg)	
																Effective score for dietary habits	
																activity	
																Effective score for sleep	
Peralta	2009	Prev Med	Australia	RCT	Physical activity	16				17				6 months		BMI	objectively measured and
									Total group 0% boysmean age 12.5 (0.4)							Waist circumference (cm)	objectively measured
																Body fat (%)	measured with body-fat analyzer
																Cardiorespiratory fitness (MFT laps	20m shuttle run test
																Small Screen Recreation weekday	self-report questionnaire
																Small Screen Recreation weekend	self-report questionnaire
																Consumption sweetened beverage	self-report questionnaire
																Consumption Fresh fruit (servings	self-report questionnaire
																Weekday MPA (min/day)	measurd with accelerometer
																Weekday VPA (min/day)	measurd with accelerometer
																Weekday MVPA (min/day)	measurd with accelerometer

																Total weekday PA (counts/min)	measurd with accelerometer
																Weekend MPA (min/day)	measurd with accelerometer
																Weekend VPA (min/day)	measurd with accelerometer
																Weekend MVPA (min/day)	measurd with accelerometer
																Total weekend PA (counts/min)	measurd with accelerometer
Pena	2021	Obesity	Chile	RCT	Nutrition	1611	11,1	0,8	42	709	11,2	0,8	35	7 months	Pre and after 4 and 7 n	BMI z	Objectively measured; WHO growth reference
					Physical activity											Waist circumference	Objectively measured
																BMI	Objectively measured
																Systolic blood pressure	Objectively measured
																Diastolic blood pressure	Objectively measured
Polman, 2023	2016/17	Psychology & Health		Randomized controllec	Physical activity	54	8,15	0,86	63	52	8,38	0,93	53,8	4 months	Baseline, mid, post	BMI-z	
					Diet (Water)	44	8,73	0,66	52,2							Water consumption	
					Physical activity + wate	51	8,84	1,07	45,1							consumption	
																Cardiorespiratory physical fitness	
Polonsky	2019	JAMA Pediatrics	USA	Cluster RCT	Physical activity	639	10,8	0,96		723	10,8	0,96		2.5 years	Bl, 1.5 year, 2.5 year	BMI percentiles	Objectively measured anc calculated with Centers for Disease Control and Prevention (CDC) 2000 reference data.
					Nutrition											BMI z-scores	Objectively measured anc calculated with Centers for Disease Control and Prevention (CDC) 2000 reference data.
																Incidence of obesity	Calculated based on seks-specific BMI-for-age percentiles
																Combined incidence of overweigth	Calculated based on seks-specific BMI-for-age percentiles
																Prevalence of obesity	Calculated based on seks-specific BMI-for-age percentiles
																Combined prevalence of overweig	Calculated based on seks-specific BMI-for-age percentiles
																School breakfast program particip	Teacher-reported
Pongutta, 2023	2018/19	Public Health	Hong Kong	Quasi-experimental	Diet	896	7,7	1,08	49,3	1779	7,8	1,05	45,9	2 years	Baseline and 7 other ti	BMI (kg/m2)	
					Other unrelated to obesity											BMI-for-age z-score	
																Height-for-age z-score	
																Incidence of wasted children (%)	
																Incidence of stunted children (%)	
																Overweight and obesity (%)	
Pope	2016	Pediatr Phys Ther	USA	Controlled trial	Nutrition	92			57	130			43	2 school years		BMI percentiles	calculated based on CDC
					Physical activity				Total groupmean age grade 1-8							Weight status %	calculated based on CDC
																High blood pressure	measured with electronic
																At risk blood pressure	measured with electronic
																Normal blood pressure	measured with electronic
																Fit in performing curl-ups	recorded by reseachers
																Fit in performing push-ups	recorded by reseachers
																Fit on the walk/run	recorded by reseachers
																Body figure perception	questionnaire
																Body figure preference	questionnaire
																Knowledge on Active Lifestyle	test
																Knowledge on Good nutrition	test
Qian	2019	Public health nutrition	China	Cluster RCT	Nutrition	2086	8 to 9 years	Means (SDs) not reported		2396	8 to 9 years	Means (SDs) not reported				Eat breakfast (every day)	Health bureau personnel administered - student-surveys
					Environment											Proper vegetable-to-meat/egg cor	Health bureau personnel administered - student-surveys
																Quantity of vegetables eaten per c	Health bureau personnel administered - student-surveys
																Quantity of fruits eaten per day (2	Health bureau personnel administered - student-surveys
																Consume diary (every day)	Health bureau personnel administered - student-surveys
																Quantity of water per day (4 cups c	Health bureau personnel administered - student-surveys
																Frequency of eating fried foods or	Health bureau personnel administered - student-surveys
																Frequency of eating (KFC or similar	Health bureau personnel administered - student-surveys
																Frequency of eating close to bedtir	Health bureau personnel administered - student-surveys
																Avarage number of students' eatin	Calculated from nine possible eating behaviours
Ramirez-Rivera	2021	International Journal o	Mexico		Nutrition	21	10,2	0,43 ?		20	10,3	0,48 ?		9 weeks	Pre, 9 weeks, 23 weeks	BMI z-score	objectively measures, WHO software
					Physical activity											Weight	objectively measured
					other											Height	objectively measured
																Waist circumference	objectively measured
																Body fat percentage	Objectively measured, bioimpedance
																Systolic blood pressure, mmHg	Objectively measured
																Diastolic blood pressure, mmHg	Objectively measured
																Cardiorespiratory fitness (VO2max	Shuttle run, Vo2 estimated
																Physical activity (daily, hours)	Self-report questionnaire, HBSC
																Sedentary activities (daily, hours)	Self-report questionnaire, HBSC
																Nutrition knowledge (points)	self-report questionnaire
Recasens	2019	Clinical Nutrition	Spain	Cluster RCT	Physical activity	272	15-16	Means (SDs) not reported		237	15-16	Means (SDs) not reported		2 years	Bl, 2 year, 4 year, 6 yea	BMI	Task Force
					Nutrition											Weight status (low weight, normal	Calculated by Garrows' criteria
																daily time spent watching televisio	Self-report questionnaire
																after-school physical activity (yes c	Self-report questionnaire
Robbins	2020	Journal School Nursing	USA	Controlled trial	Nutrition	38	11,3	0,8	31,6	43	11,9	0,8	67,4	12 weeks	Pre and post intervent	Body fat percentage	objectively measured
					Physical activity											BMI	objectively measured, CDC growht charts
					Other											BMI percentile	objectively measured, CDC growht charts
																BMI z-score	objectively measured, CDC growht charts
																Physical activity, minutes/hour	objectively measured, accelerometer
																Self-efficacy towards physical activ	self-report questionnaire
																Social support for physical activity	self-report questionnaire
																Autonomous motivation for physic	self-report questionnaire
																Controlled motivation for physical	self-report questionnaire
																Motivation for physical activity	self-report questionnaire
																Fruit intake	self-report questionnaire, 24-hour dietary assessment
																Vegetable intake	self-report questionnaire, 24-hour dietary assessment
																Diet quality score (0-100)	self-report questionnaire, 24-hour dietary assessment
																Self efficacy towards healthy eatin	self-report questionnaire
																Parental support for healhy eating	self-report questionnaire
																Peer support for healthy eating	self-report questionnaire
																Autonomous motivation for health	self-report questionnaire
																Controlled motivation for healthy	self-report questionnaire
																Motivation for healthy eating	self-report questionnaire
																Self-regulation healthy eating	self-report questionnaire
Robbins	2020	American Journal of He	USA	??	Physical activity	??	??	??	??	??	??	??	??	17 weeks	Baselined and post	Body fat (%)	Bioimpedance
																BMI (kg/m2)	Measured
																BMI (percentile)	CDC
																BMI (z-score)	CDC
																MVPA	ActiGraph GT3X-plus accelerometer
																Cardiorespiratory fitness (VO2max	Progressive shuttle run, laps converted to V02max
Robinson	1999	JAMA	USA	RCT	Physical activity	92	9	0,6	45	100	8,9	0,7	49	6 months		BMI	objectively measured and
					other											Triceps skinfold thickness (mm)	objectively measured
																waist circumference (cm)	objectively measured
																hip circumference (cm)	objectively measured

																waist-to-hip ratio	objectively measured
																TV watching (hours/week)	self-report questionnaire, and
																Videotapes (hours/week)	self-report questionnaire, and
																Video games (hours/week)	self-report questionnaire, and
																Meals in front of television	self-report questionnaire, and
																Frequency of snacking in front of t	self-report questionnaire, and
																Other SB (hours/day)	self-report questionnaire, and
																Daily servings of high-fat foods	self-report questionnaire, and
																Daily servings of highly advertised	self-report questionnaire, and
																PA, metabolic equivalent- weighte	self-report questionnaire, and
																Cardiovascular fitness	20m shuttle run test
Ronca, 2024	2018–19,	Obesity Facts	UK	Cluster Controlled	sedentary	3529	Not reported	Not reported	47,1	308	Not reported	Not reported	49	3 school years overall, 1	term 1, term 3	Overall score (PAQ-C)	
					Physical activity											PE score (PAQ-C)	
																Breaktime score (PAQ-C)	
																Outside school score (PAQ-C)	
																Sport participation score (PAQ-C)	
																Waist-to-height ratio	
Roccaldo	2017	International journal o	Italy	Cluster RCT	Nutrition	395	9,6	0,3		99	9,6	0,3		6 weeks	Bl, 6 week	A fruit/fruit juice every day	Self-report questionnaire
Roccaldo	2024	Nutrients				386	10,7	0,3	51,8	98	10,6	0,4	3,1		1 year	A second fruit every day	Self-report questionnaire
																Vegetables regularly every day	Self-report questionnaire
																Vegetables more than once a day	Self-report questionnaire
																Fish regularly (at least 2-3 mes a c	Self-report questionnaire
																Fast food restaurant more than on	Self-report questionnaire
																Pulses more than once a week	Self-report questionnaire
																Pasta/rice 5 or more mes per wee	Self-report questionnaire
																Cereals or grains (bread, etc.) for b	Self-report questionnaire
																Nuts regularly (at least 2-3 mes p	Self-report questionnaire
																Olive oil at home	Self-report questionnaire
																Skips breakfast	Self-report questionnaire
																A dairy product for breakfast	Self-report questionnaire
																Commercially baked goods for bre	Self-report questionnaire
																2 yoghurts and/or some cheese (4	Self-report questionnaire
																Sweets and candy several mee ev	Self-report questionnaire
																Adherance to a Mediterean Diet (K	Calculated from the self-report questionnaire
																BMI	Objectively measured, calculated using WHO (1995) reccommendations
																BMI category (thinnes-normal wei	International Obesity Task Force (IOTF) (Cole &
Rosário	2012	Int J Environ Res Publi	Portugal	RCT	Nutrition	231	8,3	1,2	50	233	8,2	1,2	53	6 months		BMI z-score	calculated based on IOTF cut-off
																weight status %	calculated based on IOTF cut-off
																overweight incidence	calculated based on IOTF cut-off
																overweight remission	calculated based on IOTF cut-off
																obesity incidence	calculated based on IOTF cut-off
																obesity remission	calculated based on IOTF cut-off
																Energy (kcal/day)	24-h dietary recall, by interviews
																Physical activity index	proxy-report by parents
Rosário	2017	Health promotion prac	Portugal	Cluster RCT	Other	233	8,3	1,2		231	8,2	1,2		5 months	Bl, 5 month	Diet Quality Index-International Sc	The score for each component was the sum of scores for each group in that component
																BMI	Objectivelymeasured height and weight
																BMI category	IOTF cut-points
																Physical activity	Parental report
																Variaty score (DQI-I)	Variety included the overall food group variety and within-group variety for protein source.
																Adequacy score (DQI-I)	Adequacy comprised the vegetable group, fruit, grain, fiber, protein, iron, calcium, and vitamin C.
																Moderation score (DQI-I)	Moderation included the total fat, saturated fat, cholesterol, sodium, and empty-calorie foods categorized into tiers
																Overall balance score (DQI-I)	Overall balance of diet included the proportionality of energy source of the macronutrient and fatty acid
																Vegetable intake	24-hr dietary recall obtained by nutritionists and/or trained interviewers
																Fruit intake	24-hr dietary recall obtained by nutritionists and/or trained interviewers
																Grain intake	24-hr dietary recall obtained by nutritionists and/or trained interviewers
																Fiber intake	24-hr dietary recall obtained by nutritionists and/or trained interviewers
																Protein intake	24-hr dietary recall obtained by nutritionists and/or trained interviewers
																Iron intake	24-hr dietary recall obtained by nutritionists and/or trained interviewers
																Calcium intake	24-hr dietary recall obtained by nutritionists and/or trained interviewers
																Vitamin C intake	24-hr dietary recall obtained by nutritionists and/or trained interviewers
																Total fat intake	24-hr dietary recall obtained by nutritionists and/or trained interviewers
																Saturated fat intake	24-hr dietary recall obtained by nutritionists and/or trained interviewers
																Cholesterol intake	24-hr dietary recall obtained by nutritionists and/or trained interviewers
																Sodium intake	24-hr dietary recall obtained by nutritionists and/or trained interviewers
																Empty-calorie foods intake	24-hr dietary recall obtained by nutritionists and/or trained interviewers
Rozenkranz, 2023	2018	Journal of Sport and H	United States	Cluster-randomized tri	Environment	907	9-11 yrs	Not reported	47,8	805	9-11 yrs	Not reported	47,8	4 months	Baseline, 4 mo	Physical activity (YAP score)	
					physical activity											score)	
					sedentary											Dietary intake (YAP score)	
					diet											MVPA (min/day)	
																Sedentary behaviour (min/day)	
Rush	2012	Br J Nutr	New Zealand	RCT	Nutrition	n=492, 51% girls				Control group 1 n=434, 51% girls				2 years		BMI z-score	calculated based on the 1990 UK
					Physical activity					Total groupmean age 5-7 years						% body fat sds	Bioelectrical impedance
						Intervention group 2 n=200, 51% girls				Control group 2n=226, 50% girls						resting SBP sds	measured with electronic
										Total group mean age 10-12 years						resting DBP sds	measured with electronic
Sadeghi	2019	BMC Public Health	USA	Quasi experimental	Nutrition	387	6,02	1,34		313	5,93	1,28		on-going but 941 days f	Bl and every 6 months	BMI percentiles	Calculated using the Centers for Disease Control and Prevention (CDC) reference.
																BMI	
					Physical activity											zBMI	Calculated using the Centers for Disease Control and Prevention (CDC) reference.
																Waist circumference-to-height rati	Objectively measured
Safdie	2013	Salud Publica Mex	Mexico	RCT	Nutrition	252 (Basis	9,7	0,6	48	354	9,8	0,8	49	18 months		BMI	objectively measured and
					Physical activity	254 (Plus group)	9,7	0,7	54							weight status %	calculated based on IOTF cut-off
					Environment											Intake highly recommended food	direct observation and
																Intake non recommended food	direct observation and
																Intake food recommended for con	direct observation and
																Availability highly recommended f	assessed by nutritionist at school
																Availability non- recommended fo	assessed by nutritionist at school
																Availability food recom- mended f	assessed by nutritionist at school
																Availability of potable drinking wat	direct observation
																MVPA, min during PE	direct observation
																MVPA, min during recess	direct observation
																Number of steps taken during scho	measured with pedometer
Sahota	2001	BMJ	UK	RCT	Nutrition	314	8,4	0,6	49	322	8,4	0,6	41	12 months		BMI z-score	calculated based on the 1990 UK
					Physical activity											Intake foods high in fat	24h recall and 3 day food diaries
					Environment											Intake foods and drinks high in sug	24h recall and 3 day food diaries
																Fruit intake	24h recall and 3 day food diaries

																Vegetable intake	24h recall and 3 day food diaries
																Physical activity	self-report questionnaire
																Sedentary behaviour	self-report questionnaire
																Self-perception scholastic compete	self-report questionnaire
																Self-perception behavioural condu	self-report questionnaire
																Self-perception social acceptance	self-report questionnaire
																Self-perception athletic competen	self-report questionnaire
																Self-perception physical appearan	self-report questionnaire
																Global self-worth	self-report questionnaire
																Dieting restraint	self-report questionnaire
																Body shape preference	self-report questionnaire
																Self reported behaviour change	Focus group meeting
																Sophistocation of ideas and vocab	Focus group meeting
																Willingness and confidence to sha	Focus group meeting
																Basic knowledge	Focus group meeting
																Greater understanding of the healt	Focus group meeting
Sahota	2019	Pilot and feasibility stu	UK	RCT	Nutrition	188	7,2	1,1	49	170	7,2	1,1	49	17 months (2 academic	Pre and post	Nutrition knowledge	Self report questionnaire
					Physical activity											Healthy/balance diet knowledge	Self report questionnaire
																Physical activity knowledge	Self report questionnaire
																Overall healthy lifestyle knowledg	Self report questionnaire
																Water intake	Self report questionnaire
																Diet fizzy drinks intake	Self report questionnaire
																Full sugar fizzy drinks intake	Self report questionnaire
																Intake of chocolate, biscuits, swee	Self report questionnaire
																Pies and patries intake	Self report questionnaire
																Take-away style foods (pizza, keba	Self report questionnaire
																Crisps intake	Self report questionnaire
																Meat and meat alternatives (incluc	Self report questionnaire
																Fruit (including dried fruit) intake	Self report questionnaire
																Vegetables (including tomatoes, b	Self report questionnaire
																Attitude towards vegetables (like c	Self report questionnaire
																Attitude towards fruits (like or not	Self report questionnaire
																Attitude towards sports (like or no	Self report questionnaire
																Body shape satisfaction (as part of	Self report questionnaire
																Dietary restraint score (dieting beh	Self report questionnaire
																BMI SD	Self report questionnaire? WHO growth reference
Shang	2020	Preventive Medicine	China	RCT	Nutrition	Only Diet: 631	9,2	1,5	51	468	9,2	1,5	42	1 year	Pre and post	Clustered metabolic risk score (fat	Objectively measured
					Physical activity	Only PA: 599	9,1	1,6	48								
						Combined: 3501	9,1	1,4	49	3373	9	1,4	50				
Sanchez-Martinez	2021	Nutrients	Spain	Controlled trial	Nutrition	1151	8-9 year olds at baseline		49	1230	8-9 year olds at baseline		52	3 years	Pre and post	Overweight/obesity prevalence, b	Objectively measured
					Physical activity											Dinner in front of TV (y/n)	
																Eating at school (y/n) (1 healthy meal per day)	
																Proper breakfast (y/n) (carbohydrates, dairy and fruits)	
																Frequenting fast-food restaurants (y/n)	
																Eating alone (y/n) (better accompanied)	
																Out-of-school physical activitty (y/n)	
																Leisure time physical activity (more or less than 2 h/wk)	
																Screen time on weekdays (Mon-Thurs) (more or less than 2 h/day)	
																Screen time on weekend days (Fri-Sun) (more or less than 2 h/day)	
																Frequency of water	
																Frequency of fruits and vegetables	
																Frequency of meat	
																Frequency of occasional sweets	
																Frequency of occasional fried potatoes	
																Global nutrition score	
Sallis	1993	Ann N Y Acad Sci	USA	RCT	Physical activity	led intervention)			44	198			49	2 years		BMI	objectively measured and
						151 (Specialistled intervention)			40	Total group: 9.3 (0.5) years						sum of skinfold thickness (calf and	objectively measured and
Salmon	2008	Int J Obes (Lond)	Australia	RCT	Physical activity	BM group: n=66				62				2 years		BMI	objectively measured and
						FMS group: n=74				Total group: 10.8 (0.4) years, 51% girls						weight status %	calculated based on IOTF cut-off
						BM/FMS group: n=93										TV viewing (min/week)	self-report questionnaire
																Computer use (min/week)	self-report questionnaire
																Electronic games use (min/week)	self-report questionnaire
																Counts per day	measured with accelerometer
																Moderate PA (min/day)	measured with accelerometer
																Vigorous PA (min/day)	measured with accelerometer
																Fundamental movement skills z-sc	assessed by trained staff
																PA enjoyment	self-report questionnaire
Salmon	2023	Br J Sports Med	Australia	RCT	Physical activity	PA: n=161				149				30 months	Bl, 18 months, 30 mon	Sedentary time, weekday (min)	Accelerometer-based
					other	SB: n= 124				Total group: 8.4 (0.7), 57% girls						Sedentary time, average day (min)	Accelerometer-based
						PA + SB: n=159										Sedentary breaks, weekday (freq)	Accelerometer-based
																Sedentary breaks, average day (fre	Accelerometer-based
																MVPA, weekday (min)	Accelerometer-based
																MVPA, average day (freq)	Accelerometer-based
																BMI z-score	objectively measured and calculated using US data
																Waist circumference (cm)	objectively measured
																Systolic blood pressure (mmHg)	objectively measured
																Diastolic blood pressure (mmHg)	objectively measured
Santos	2014	JAMA Pediatr	Canada	RCT	Physical activity	340	9,3	range 9.1-9.3	54	307	8,8	range 8.6-9.0	48	9 months		BMI z-score	calculated based on CDC
					Nutrition											waist circumference	objectively measured and
					other											Average daily step count	measured with pedometer
																Activity frequency	self-report questionnaire
																Cardiovascular fitness	20-m shuttle run
																Frequency of food intake	self-report questionnaire
																Frequency of drink intake	self-report questionnaire
																Self-esteem/self-efficacy	self-report questionnaire
																Nutrition knowledge Food	self-report questionnaire
																Nutrition knowledge Drinks	self-report questionnaire
																Healthy habits	self-report questionnaire
																Dieting behavior/thoughts	self-report questionnaire
Santos-Beneit	2024	Journal of the America	Spain	RCT	Physical activity	972	6,3	0,3	50	798	6,4	0,3	44,2	6 years	Bl, 3 years, 6 years	BMI-z	objectively measured and calculated based on CDC
					Nutrition											WC-z	objectively measured and calculated based on Third National Health and Nutrition Examination Survey cutoff points
																waist-to-height ratio	objectively measured and calculated based on Third National Health and Nutrition Examination Survey cutoff points
																KAH overall score	knowledge-attitudes-habits (KAH) survey

																Knowledge	knowledge-attitudes-habits (KAH) survey
																Attitude	knowledge-attitudes-habits (KAH) survey
					other											Habits	knowledge-attitudes-habits (KAH) survey
Scherr	2017	J Nutr Educ Behav	USA	RCT	Nutrition	245	9,5	0,4	49	198	9,6	0,4	52	6 months		BMI z-score	objectively measured and calculated based on CDC growth charts
					Physical activity											BMI percentiles	calculated based on CDC
					Environment											waist-to-height ratio	objectively measured and calculated
																weight status %	calculated based on CDC
																nutrition knowledge	self-report questionnaire
																vegetable identification	self-report questionnaire
																vegetable preferences	self-report questionnaire
																Science process skills	skill test
																Fruit intake, servings	self-report questionnaire
																Vegetable intake, servings	self-report questionnaire
																dietary-related parenting practices	self-report questionnaire,
																Authorative parenting	self-report questionnaire,
																measures of restriction	self-report questionnaire,
																pressure to eat	self-report questionnaire,
Scherr	2021	Health Promotion Prac	United States	Non-randomised, 3 grc	Nutrition	7 (education grp)	Not reported	Not reported	56,9	25	Not reported	Not reported	50	1 academic year (~9mo)	Pre (Sept), post (June)	Nutrition knowledge score	
						75 (extender grp)	Not reported	Not reported	49,4							BMI percentile-for-age	
																Vegetable identification	
Schwartz	2016	JAMA Pediatr	USA	Controlled trial	Nutrition	n=374 schools				n=482 schools				1 school year?		BMI z-score	calculated based on CDC growth charts
					Environment					Total group, n=1065562 children, 50% girls, mean age Kindergarten-8th grade						weight status %	calculated based on CDC
																total milk purchase	data obtained from NYCDOE
																Mean total milk half-pints, purchas	data obtained from NYCDOE
																Mean chocolate milk half-pints, pu	data obtained from NYCDOE
																Mean low-fat/skim milk half-pints,	data obtained from NYCDOE
Sevinc	2008	Turk J Med Sci	Turkey	RCT	Nutrition	vention group 1)			50	2821			49	8 months		BMI	objectively measured and
					Physical activity	vention group 2)			50	Total groupmean age grde 1-7							
Sgambato	2019	British Journal of Nutri	Brazil	RCT	Nutrition	1290	11,5	1,43	48,1	1157	11,5	1,46	47,9	one school year	Pre and post intervent	BMI	objectively measured
					Physical activity											Body fat percentage	objectively measured
																Milk intake	self-report questionnaire
																Beans intake	self-report questionnaire
																Water intake	self-report questionnaire
																Fruits intake	self-report questionnaire
																Vegetables intake	self-report questionnaire
																Cookies intake	self-report questionnaire
																Sodas intake	self-report questionnaire
																Pocessed meat intake	self-report questionnaire
																Fruit-based drinks intake	self-report questionnaire
																Physical activity, minutes per week	self-report questionnaire
Shamah Levy	2012	BMC Public Health	Mexico	RCT	Nutrition	510			52	509			50	7 months		BMI	objectively measured and
					Physical activity					Total groupmean age 5th grade						weight status %	calculated based on IOTF
					Environment											Dietary intake, Energy (Kcal)	proxy-report questionnaire,
																Dietary intake, Carbohydrates (g)	proxy-report questionnaire,
																Dietary intake, Lipids (g)	proxy-report questionnaire,
																PA status %	proxy-report questionnaire (?)
																Eating knowlegde	proxy-report questionnaire (?)
																PA knowledge	proxy-report questionnaire (?)
																Eating Self-efficacy level	proxy-report questionnaire (?)
																PA Self-efficacy level	proxy-report questionnaire (?)
Shofan	2011	Eur J Clin Nutr	Israel	Controlled trial	Nutrition	82				27				2 years		height (cm)	objectively measured
					Physical activity					Total group 50% girls mean age 9.0 (0.4)						weight (kg)	objectively measured
																BMI	objectively measured and
																BMI percentiles	reference data ?
Sichieri	2009	Public Health Nutr	Brazil	RCT	Nutrition	526	10,9	0,8	53	608	10,9	0,8	53	7 months		BMI	objectively measured and
Sichieri	2013															weight status %	calculated based on IOTF cut-off
																weight	objectively measured
																Carbonated beverages (ml/d) per c	24h recall self-report
																Daily intake of juices (ml)	24 recall self-report questionnaire
																Water (glasses/day)	*24 recall self-report questionnaire*
																Sodas (glasses/day)	*24 recall self-report questionnaire*
																Guaraná (glasses/day)	*24 recall self-report questionnaire*
																Fruit juice (glasses/day)	*24 recall self-report questionnaire*
																Milk (glasses/day)	*24 recall self-report questionnaire*
Siegrist	2013	Scand J Med Sci Sports	Germany	RCT	Physical activity	427				297				1 year		BMI	objectively measured and
					Environment					total group: 48% girls,mean age 8.4 (0.7)						BMI z-score	calculated based on IOTF cut-off
																Waist circumference (cm)	objectively measured
																weight status %	calculated based on German reference data
																PA days/week (>60 min)	self-report questionnaire
																Participation in local sport club	self-report questionnaire
																Physical fitness (points, t-score)	Munich fitness test
																Consuming media (computer use, T	self-report questionnaire
Siegrist	2018	Atherosclerosis	Germany	Cluster RCT	Physical activity	331	11,1	0,6		257	11,1	0,6		18 months	Bl ,18 month	BMI	Objectively measured and calculated
					Other											BMI-SDS	Determined using the LMS method using national reference data.
																systolic blood pressure (mm/Hg)	Objectively measured after 5 min rest
																diastolic blood pressure (mm/Hg)	Objectively measured after 5 min rest
																Total cholesterol (mg/dl)	Objectively measured (using standard procedures manufacturer's recommedations)
																HDL (mg/dl)	Objectively measured (using standard procedures manufacturer's recommedations)
																Triglycerides (mg/dl)	Objectively measured (using standard procedures manufacturer's recommedations)
																Glucose (mg/dl)	Objectively measured (using standard procedures manufacturer's recommedations)
																Leptin (ng/nl)	Objectively measured (using commercially available quantative sandwich ELISAs)
																Adiponectin (ug/ml)	Objectively measured (using commercially available quantative sandwich ELISAs)
																Insulin (uU/ml)	Objectively measured (automated immunoanalyzer system)
																Il-6 (pg/ml)	Objectively measured (automated immunoanalyzer system)
																hsCRP (mg/L0	Objectively measured (high sensitive C-reactive protein by an immunoturbodimetric CPR-Latex assas)
																Central Retinal Arteriolar Equivaler	Objectively measured and researcher observed (Static Retinal Vessel Analyser)
																Central Retinal Venular Equivalent	Objectively measured and researcher observed (Static Retinal Vessel Analyser)
																Arteriolar-to-venular diameter rati	Objectively measured and researcher observed (Static Retinal Vessel Analyser)
																Physical fitness (points)	Objectively measures (6-item test battery)
																Physical activity (days/week)	self-report questionnaire
																Media use (h/day)	self-report questionnaire
																PA/school (min/week)	self-report questionnaire
Sigmund	2012	BMC Public Health	Czech Republic	Controlled trial	Physical activity	88			49	88			47	2 years		weigth status %	calculated based on WHO
Sigmund	2013	Int J Environ Res Public Health								total groupmean age 6.7 (0.5)				4 years		Mean daily steps counts, schoolda	measured with accelerometer,

																Mean daily steps counts, schoolda	measured with accelerometer,
																Mean daily steps counts, schoolda	measured with accelerometer,
																Mean daily steps counts, weekend	measured with accelerometer,
																Mean daily activity energy expend	measured with accelerometer,
																Mean daily activity energy expend	measured with accelerometer,
																Mean daily activity energy expend	measured with accelerometer,
																Mean daily activity energy expend	measured with accelerometer,
Simon	2008	Int J Obes (Lond)	France	RCT	Physical activity	475	11.6	0.6	52.6	479	11.7	0.7	47.4	4 years	Bl, yearly measurements (blood samples every 2 years	BMI (kg/m2)	Objectively measured
Simon	2014	Int J Obes (Lond)														BMI, age and gender adjusted	Calculated
																Body fat (%)	Objectively measured
																Fat mass index (kg/m2)	Calculated
																Fat-free mass index (kg/m2)	Calculated
																Overweight prevalence (%)	Calculated
																Supervised leisure physical activity (hours/week)	Self-report questionnaire
																TV/video time (min/day)	Self-report questionnaire
																Active commuting, home-school (min/day)	Self-report questionnaire
																Intention towars physical activity, score	Self-report questionnaire
																Self-efficacy (physical activity), score	Self-report questionnaire
																Plasma glucose (mg/100 ml)	Objectively measured
																Plasma insulin (µU/ml)	Objectively measured
																HOMA	Calculated
																Plasma triglycerides (mg/100 ml)	Objectively measured
																Plasma total cholesterol (mg/100 ml)	Objectively measured
																Plasma HDL cholesterol (mg/100 ml)	Objectively measured
																Systolic blood pressure (mm Hg)	Objectively measured
																Diastolic blood pressure (mmg Hg)	Objectively measured
Smith	2013	J Sch Nurs	USA	RCT	Physical activity	72				88				8 weeks		BMI	objectively measured and
					Nutrition				Total group 48% girlsmean age 9.2 (0.7)							Resting SBP	objectively measured
																Resting DBP	objectively measured
																Healthy eating habits	self-report questionnaire
																PA habits	self-report questionnaire
																Nutritional knowledge	self-report questionnaire
																Attitudes towards eating healthfull	self-report questionnaire
																Attitudes towards PA	self-report questionnaire
																Perceived support	self-report questionnaire
																Self-efficacy	self-report questionnaire
																Intention	self-report questionnaire
Speroni	2007	J Sch Nurs	USA	Controlled trial	Nutrition	80	9,4		57	105	9,2		44	24 weeks		BMI percentiles	calculated based on CDC growth
					Physical activity											Waist circumference	objectively measured
																Body self-perception	self-report questionnaire
																Satisfaction with food choices	self-report questionnaire
																Satisfaction with activitychoices	self-report questionnaire
																Satisfaction with family food choic	self-report questionnaire
																Satisfaction with family activity ch	self-report questionnaire
Spiegel	2006	Obesity (Silver Spring)	USA	RCT	Nutrition	534				479				1 school year		BMI	objectively measured and
					Physical activity				Total group mean age grade 4-5							Weight status %	calculated based on CDC growth
																PA during schoolday, min/week	self-report questionnaire
																PA outside school, min/week	self-report questionnaire
																Consumption of fruit	self-report questionnaire
																Consumption of green salad	self-report questionnaire
																Consumption of potato	self-report questionnaire
																Consumption of carrots	self-report questionnaire
																Consumption of other vegetables	self-report questionnaire
																Consumption of 100% fruit juice	self-report questionnaire
Stea	2018	PLoS One	USA	Cluster RCT	Nutrition	322	26,5		0,53	759	26,5		0,51	1 year	Bl, 1 year, 2year, 4 yea	Perceived weight status	Self-reported
															Bl, 4 year, 14 year	BMI	Calculated based on self-reported height and weigth
																Weight status (overweight or obes	WHO definition
Story	2003	Ethnicity and Disease	USA	RCT	Nutrition	26	9.4	0.9	100	28	9.1	0.8	100	12 weeks	Bl and 12 weeks	BMI (kg/m2)	Objectively measured
					Physical activity											Waist circumference (cm)	Objectively measured
																Total counts/min	Objectively measured
																MVPA (min)	Objectively measured
																GEMS activity questionnaire, mets-adjusted physical activity score	Self-report questionnaire
																Fruit juice and vegetable (servings/day)	Self-report questionnaire
																Water (servings/day)	Self-report questionnaire
																Total energy intake (Kcal)	Self-report questionnaire
																% energy from fat	Self-report questionnaire
																Healthy choice behavioral intentions	Self-report questionnaire
																Self efficacy for healthy eating	Self-report questionnaire
																Diet knowledge	Self-report questionnaire
																Fruit and vegetable snack availability	Self-report questionnaire
																Parent encouragement for healthy eating	Self-report questionnaire
																Physical activity self-concept	Self-report questionnaire
																Physical activity preference	Self-report questionnaire
																Sedentary activity preference	Self-report questionnaire
																Positive expectance for physcial activity	Self-report questionnaire

																Self efficacy physical actcivity	Self-report questionnaire
																Physical activity home environment	Self-report questionnaire
																Silhouettes—look like you	Self-report questionnaire
																Silhouettes—like to look	Self-report questionnaire
																Silhouettes - difference (current-ideal)	Self-report questionnaire
																Weight concern—moderate behaviors	Self-report questionnaire
																Weight concern—unhealthy behaviors	Self-report questionnaire
																Tried to loose weight (% sometimes or a lot)	Self-report questionnaire
																Availability of higher-fat foods	Parent-report questionnaire
																Availability of lower-fat foods	Parent-report questionnaire
																Low-fat food practices	Parent-report questionnaire
																Motivation for healthy eating	Parent-report questionnaire
																Self-efficacy for healthy food preparation	Parent-report questionnaire
																Sweetened beverage availability	Parent-report questionnaire
																Vegetable availability	Parent-report questionnaire
																Fruit availability	Parent-report questionnaire
																Bottled water availability	Parent-report questionnaire
																% energy from fat	Parent-report questionnaire
																Fruit intake	Parent-report questionnaire
																Vegetable intake	Parent-report questionnaire
																Motivation for physical activity	Parent-report questionnaire
																Self-efficacy for physical activity with daughter	Parent-report questionnaire
																Parent support of daughter's activity level	Parent-report questionnaire
																Daughter TV watching	Parent-report questionnaire
Stock	2007	Pediatrics	Canada	Prospective, controlled	Physical activity	228	4th to 7th grade		56,1	132	4th to 7th grade		53,8	School year	Baseline (Sept), FU (June)	BMI	Calculated
																Height	Objectively
																Weight	Objectively
																Fitness	9 minute run
																Body image perception	Figural Rating Scale
																Food preoccupation	Healthy Living Questionnaires designed by study team
																Knowledge about nutrition	Healthy Living Questionnaires designed by study team
																Knowledge about physical activity	Healthy Living Questionnaires designed by study team
																Frequency of healthy eating	Healthy Living Questionnaires designed by study team
																Frequency of exercise	Healthy Living Questionnaires designed by study team
																Frequency of healthy-living behaviours	Healthy Living Questionnaires designed by study team
																Health attitudes	Healthy Living Questionnaires designed by study team
																Emotional health	Healthy Living Questionnaires designed by study team
																Self-competence - cognitive (emphasizing academic performance_	Perceived Competence Scale for Children or pictorial scale of Perceived Competence and Social Acceptance for Young Children
																Self-competence - social (emphasizing peer relationships),	Perceived Competence Scale for Children or pictorial scale of Perceived Competence and Social Acceptance for Young Children
																Self-competence - physical (emphasizing sports and outdoor games)	Perceived Competence Scale for Children or pictorial scale of Perceived Competence and Social Acceptance for Young Children
																Self-worth	Perceived Competence Scale for Children or pictorial scale of Perceived Competence and Social Acceptance for Young Children
																Disordered eating symptoms	Children's Eating Attitude Test (ChEAT)
																Blood pressure (mmHg)	Objectively
																Heart rate (bpm)	Objectively
Tarro	2014	Trials	Spain	RCT	Nutrition	1222				717				28 months		Weight status %	calculated based on IOTF cut-off
					Physical activity				Total group 50% girlsmean age 8.4 (0.6)							BMI	objectively measured and
																BMI z-score	calculated based on WHO Global
																Waist circumference (cm)	objectively measured
																Consumption of breakfast	self-report questionnaire
																Consumption of dairy products	self-report questionnaire
																Consumption of cereals	self-report questionnaire
																Consumption of pastry	self-report questionnaire
																Consumption of fruit or natural jui	self-report questionnaire
																Consumption of vegetables, daily	self-report questionnaire
																Consumption of vegetables, >1 pe	self-report questionnaire
																Consumption of fish, regularly	self-report questionnaire
																Consumption of fast food, >1 per v	self-report questionnaire
																Consumption of legumes >1 per w	self-report questionnaire
																Consumption of candy >1 per day	self-report questionnaire
																Consumption of pasta or rice, daily	self-report questionnaire
																Cooking with olive oil at home	self-report questionnaire
																Consumption of sandwich	self-report questionnaire
																Consumption of juice packaged/so	self-report questionnaire
																TV or video games, h/day	self-report questionnaire
																After-school PA, h/week	self-report questionnaire
																Leisure-time activity in park or gar	self-report questionnaire
																Leisure-time activity in park or gar	self-report questionnaire
																Sport with father >3 h/week	self-report questionnaire
																Sport with mother >3 h/week	self-report questionnaire
Taylor	2006	Int J Pediatr Obes	New Zealand	Controlled trial	Nutrition	207	8	1,7	46	177	7,9	1,5	50	1 year		BMI z-score	calculated based on CDC growth
					Physical activity											Waist circumference (cm)	objectively measured
																Pulse rate (counts)	measured with automated
																Resting SBP	measured with automated
																Resting DBP	measured with automated
																Average accelerometry counts	measured with accelerometer

																Time in light activity	measured with accelerometer
																Time in moderate activity	measured with accelerometer
																Time in vigorous activity	measured with accelerometer
																Time in moderate/vigorous activity	measured with accelerometer
																Time in sedentary activity	measured with accelerometer
Taylor	2007	Am J Clin Nutr	New Zealand	Controlled trial	Nutrition	250	7,7	1,8	45	219	7,7	1,6	51	2 years		height z-score	calculated based on CDC
Taylor	2008	Am J Clin Nutr			Physical activity									4 years		weight z-score	calculated based on CDC
																BMI z-score	calculated based on CDC
																weight status %	calculated based on CDC
																waist circumference	objectively measured
																resting systolic BP (mmHg)	measured with automated
																resting diastolic BP (mmHg)	measured with automated
																Pulse rate (beats/min)	measured with automated
																Carbonated beverages (servings)	self-report questionnaire
																Fruit drinks or juice (servings)	self-report questionnaire
																Flavored milk (servings)	self-report questionnaire
																Water (servings)	self-report questionnaire
																Total sweet drinks (servings)	self-report questionnaire
																Fruit (servings)	self-report questionnaire
																Vegetables (servings)	self-report questionnaire
																television viewing time	self-report questionnaire
																physical activity	self-report questionnaire
																mean accelerometry counts	measured with accelerometer
Telford	2012	Am J Public Health	Australia	RCT	Physical activity	312				308				2 years		BMI	objectively measured and
										Total groupmean age grade 3						% body fat	calculated based on DXA data
																PA, steps/day	measured with pedometer
																Cardiorespiratory fitness	20m shuttle run test
																Numeracy	test administered by teacher
																Writing skills	test administered by teacher
																Reading skills	test administered by teacher
Teo	2021	Nutrients	Malaysia	RCT	Nutrition	251	8,9	1,4	43	272	9	1,4	54	3 months	Pre, post and 3 months	Frequency of breakfast consumpti	Self report questionnaire
					Environment											Frequency of lunch consumption	Self report questionnaire
					Physical activity											Frequency of dinner consumption	Self report questionnaire
																Frequency of morning tea snacking	Self report questionnaire
																Frequency of afternoon tea snacki	Self report questionnaire
																Frequency of supper snacking	Self report questionnaire
																Physical activity score	Self report questionnaire; Level of PA (5-point scale; PAQ-C)
																Body weight	Objectively measured
																Height	Objectively measured
																BMI for age Z score	Objectively measured
																Cognitive performance	Objectively measured; Raven's Colored Progressive Matrices (CPM)
Thivel	2011	Eur J Pediatr	France	RCT	Physical activity	229			51	228			49	6 months		Weight (kg)	objectively measured
										Total groupmean age 6-10 years						Waist circumference (cm)	objectively measured
																BMI	objectively measured
																Skinfold thickness, sum of biceps, t	objectively measured
																Fat free mass (FFM) (kg)	objectively measured
																Cycling peak power (W)	measured with ergometer
																Cycling peak power (W/kg)	measured with ergometer
																Cycling peak power (W/kgFFM)	measured with ergometer
																Cardiorespiratory fitness (stage)	20-m shuttle run
																Heart rate peak (bpm)	measured with heart rate
																Heart rate rest (bpm)	measured with heart rate
																Heart rate end (bpm)	measured with heart rate
																Heart rate reserve (bpm)	measured with heart rate
Treu	2015	Am J Health Promot	USA	Quasi-experimental	Nutrition	416 (SI group)	8,7	0	46	559	8,7	0,4	48	1 school year		Weight, pounds	objectively measured
					Physical activity	512 (EI group)	8,7	0,4	49							BMI	objectively measured and
																BMI z score	calculated based on CDC
																VO2max	15 or 20m shuttle run test
																Abdominal strength	measured by Curl-ups
																Upper body strength	measured by 90-degree
																Back extensor	measured by Trunk Lift
																Left flexibility	measured by Back Saver Sit &
																Right flexibility	
																Asthma medication use n(%)	data collected by school nurse
																ADHD medication use n(%)	data collected by school nurse
																Reading fluency	Academic performance tests
																Reading comprehension	Academic performance tests
																Math computation performance	Academic performance tests
																Math problem solving skills	Academic performance tests
																Referrals for disciplinairy action	recorded by teachers
																Classroom behaviour	recorded by teachers
																Nutrition knowledge	self-report questionnaire
Tucker	2011	J Community Health		Quasi-experimental	Nutrition	41 (EH-school)				29 (EH-school)				1 school year		BMI	objectively measured and
			USA	Controlled trial	Physical activity	25 (HB-school)				Total group 53% girls, mean age grade 4-5						BMI percentile	calculated based on CDC growth
					Environment					4 (HB-school)						Weight status %	calculated based on CDC growth
										Total group 48% girls,mean age grade 4-5						Servings of fruits or vegetables per	Interview: Healthy Habits Survey
																Dinners with family per week	Interview: Healthy Habits Survey
																Breakfasts per week	Interview: Healthy Habits Survey
																Fast food per week	Interview: Healthy Habits Survey
																Servings of 100% fruit juice per day	Interview: Healthy Habits Survey
																Servings of fruit/sports drinks per d	Interview: Healthy Habits Survey
																Servings of soda/punch per day	Interview: Healthy Habits Survey
																Minutes of TV per day	Interview: Healthy Habits Survey
																TV in room	Interview: Healthy Habits Survey
																PC in room	Interview: Healthy Habits Survey
																Average steps/day	measured with pedometer
van den Berg	2020	Childhood obesity	USA	RCT	Nutrition	336 (WAT)	72,5% ≤8yrs, 27,5% ≥9yrs		48,5	285	72,4% ≤8yrs, 27,6% ≥9yrs		54,7	one school year	Pre and post intervent	Vegetable preference	self-report questionnaire
					Physical activity	347 (LGEG)	75,5% ≤8yrs, 24,5% ≥9yrs		50,1							Vegetable tasted	self-report questionnaire
						358 (combined)	62,7% ≤8yrs, 37,3% ≥9yrs		50,4							Nutrition knowledge	self-report questionnaire
																Gardening together with parents	self-report questionnaire
																Vegetable consumption (previous	self-report questionnaire
																Moderate or vigorous physical acti	self-report questionnaire
																Sedentary behaviour, hours per da	self-report questionnaire
																Home availability vegetables	parent-report questionnaire

																Engaging in physical activity togeth	parent-report questionnaire
																BMI percentile	unknown, not reported
Verjans-Janssen	2020	IJBNPA	the Netherlands	Controlled trial	Nutrition	419	8,5	1,1	54	104	8,5	1,1	54	2 years ?	Pre, after 1 and 2 years	BMI z-score	Objectively measured, Dutch reference data
					Physical activity											% of time spent in sedentary behav	Objectively measured; accelerometer
																% of time spent in light physical ac	Objectively measured; accelerometer
																% of time spent in moderate-to-vig	Objectively measured; accelerometer
																Vector magnitude Counts per minu	Objectively measured; accelerometer
																Consumption of breakfast	Self report questionnaire
																Consumption of fruit and vegetabl	Self report questionnaire
																Consumption of candy, coockies or	Self report questionnaire
																Consumption of sugar-sweetened	Self report questionnaire
																Consumption of water at school	Self report questionnaire
																Adherence fruit recommendation	Parent report questionnaire
																Adherence vegetable recommenda	Parent report questionnaire
																Daily consumption of snacks	Parent report questionnaire
																Daily consumption of sugar-sweete	Parent report questionnaire
																Daily consumption of water	Parent report questionnaire
Vieira, 2021	2011/12	International Journal o	Portugal	Non-randomised contr	Nutrition	219	11,2	0,6	47,5	230	11,2	0,6	51,7	8 months	Baseline, 8 month FU	Height (cm)	
					Physical activity											Height z-score	
																Weight (kg)	
																Weight z-score	
																BMI (kg/m2)	
																BMI z-score	
																Waist circumference (cm)	
																Waist-to-height ratio	
																(servings/day)	
																Soft drink intake (servings/day)	
																(servings/day)	
																High-fat food (servings/day)	
																(servings/day)	
																Physical activity (min/day)	
																(min/day)	
Viggiano	2018	European Journal of Pe	Italy	Cluster RCT (pilot)	Nutrition	837	7 to 11			476	7 to 11			8 months	Bl, 8 month, 18 month	BMI z-score	Objectively measured and calculated
					Physical activity											Physical activity: frequency (time/v	Children (HBSC) physical activity questionnaire)
																Physical activity: duration (min/we	Children (HBSC) physical activity questionnaire)
																Junk food intake	self-report questionnaire (food diary)
																Healthy food intake	self-report questionnaire (food diary)
Wadolowska	2019	Nutrients	Poland	RCT	Nutrition	319	11,9	0		145	11,9	0,1		9 months	Bl, 9 month	BMI-for-age	Objectivley measured and calculated
					Other											z- WHtR	Objectivley measured and calculated
																z-waist circumference	Objectivley measured and calculated
																Dietary data	Self-report via short-form food frequency questionnaire (SF-FFQ4PolishChildren)
																Sscreen time	Self report questionnaire
																Adherence to WHO recommendat	Self report questionnaire
																Diet quality score - pro-helathy pH	Self report questionnaire
																pHDI (pro-healthy diet index) > Me	Self report questionnaire
																Diet quality score - non-healthy nH	Self report questionnaire
																nHDI (non-healthy diet index) > Me	Self report questionnaire
																nutrition knowledge score	Self report questionnaire
Walther	2009	Circulation	Germany	RCT	Physical activity	109	11,1	0,7	47	73	11,1	0,7	42	1 year		BMI percentiles	calculated based on German
																BMI z-score	calculated based on German
																Fat-free mass %	Bioelectrical impedance
																Total body water content %	Bioelectrical impedance
																VO2max, mL/min/kg	Measured with spirometry
																VO2 per beat, mL/beat/kg	Measured with spirometry
																Physical work capacity, W	Measured with spirometry
																Max heart rate, bpm	Measured with spirometry
																Max SBP, mmHg	Measured with spirometry
																Max DBP, mmhg	Measured with spirometry
																Motor quotient score	motor functions: balancing backward, jumping over a certain height, jumping sideways, and shifting platforms sideways
																CD45low/CD34+/KDR+, 1/106 leuk	flow cytometry in blood
																CD34+/KDR+, 1/mL blood	flow cytometry in blood
																CD34, 1/mL blood	flow cytometry in blood
																Migratory capacity, cells/10*3 seed	measured in blood sample
																HDL cholesterol, mmol/L	blood sample analysis
																LDL cholesterol, mmol/L	blood sample analysis
																Total cholesterol, mmol/L	blood sample analysis
																Triglycerides, mmol/L	blood sample analysis
Wang	2018	International journal o	China	Cluster RCT	Physical activity	5275	10,5	0,02		4583	10,5	0,02		1 year	Bl, 1 year	BMI	Objectively measured
																BMI z-score	for Chinese children
																Weigth status (overweight and obe	Assessed according to the specifically recommended age- and gender-specific references for Chinese children
																Weigth status (obese %)	Assessed according to the specifically recommended age- and gender-specific references for Chinese children
																The mean value of the 7 days' MVI	self-report PA questionnaire (CPAIQ Children Physical Activity Item Questionnaire)
																The percentage of students with in	self-report PA questionnaire (CPAIQ Children Physical Activity Item Questionnaire)
																Consumption of red meat (n / 7 da	self-report via validated food frequency questionnaire
																Consumption of Soft drinks (n / 7 d	self-report via validated food frequency questionnaire
Wang, 2022	2013/14	Frontiers in Pediatrics	China	Multi-center, cluster-c	Environment	30.997	11	3,3	48,8	27477	10,7	3,3	48,8	6 months	Baseline, 6.7 month FU	(%)	
					Physical activity											plain bottled water or sugar-	
					Diet											dietary pagoda should be eaten	
					Family											health (%)	
																status through effort (%)	
																vegetables and less high-energy	
																Belief, exercise to lose weight (%)	
																servings/day (%)	
																beverages, 0 cups/week (%)	
																days/week (%)	
																days/week (%)	
Warren	2003	Health Promot Int	UK	RCT	Nutrition	56 (Eat Smart)	6,1	0,6	45	54	6,1	0,6	46	20 weeks		BMI	objectively measured and
					Physical activity	54 (Play Smart)	6,1	0,6	50							weight status %	calculated based on IOTF cut-off
						54 (Eat/Play	6,1	0,7	56							Nutrition Knowledge	questionnaire
																Mode of transportation to school	proxy-report questionnaire by
																Activities at break times	proxy-report questionnaire by
																PA outside school	proxy-report questionnaire by
																Consumption of vegetables, per we	24-hour recall + Food frequency questionnaire, by parents
																Consumption of salads, per week	24-hour recall + Food frequency questionnaire, by parents

																Consumption of fresh fruit, per we	24-hour recall + Food frequency questionnaire, by parents
																Consumption of other fruit, per we	24-hour recall + Food frequency questionnaire, by parents
																Consumption of confectionery, per	24-hour recall + Food frequency questionnaire, by parents
																Consumption of crisps, per week	24-hour recall + Food frequency questionnaire, by parents
																Consumption of fat	24-hour recall + Food frequency questionnaire, by parents
																Consumption of fiber	24-hour recall + Food frequency questionnaire, by parents
Waters	2017	BMC public health	Australia	Cluster RCT	Other	1426	4 to 13			1460	4 to 13			3,5 year	Bl, 3,5 year, 5 year	BMI z-score	reference curves
																weight (kg)	Objectively measured
																bmi	Objectively measured and calculated
																waist (cm)	Estimated by Baseline school mean bmi z-score used as proxy as waist not measured at baseline
																overweight/ obese	calculated using WHO reference and cut-off points
																obese	calculated using WHO reference and cut-off points
																F+V intake	Parent report; child self-report; lunchbox assesssment
																Sugary drink consumption	Parent report; child self-report; lunchbox assessment
																Family food habits	Parent report; child self-report;
																Sedentary behaviour	Parent report; child self-report;
																Physical activity	Parent report; child self-report;
																Activity intensities	Parent report; child self-report;
																Child experience	Child self-report QoL via Kidscreen; child focus groups
																Impacts on the school, home and c	activities; observations (SOPLAY); Process evaluation using monitoring maps, photos, and audit; Parent report of funds
Weber	2017	Experimental and Clini	Germany	randomised	Physical activity	70	9	0,7		125	9,3	0,8		10 months	Bl, 6 month, 10 month	Physical fitness and motor skills (C	Researcher-administered CHECK test
																10 m sprint (SDS lms)	Researcher-administered CHECK test
																Ball legs wall (SDS lms)	Researcher-administered CHECK test
																Obstacle race (SDS lms)	Researcher-administered CHECK test
																Medicine ball throw (SDS lms)	Researcher-administered CHECK test
																Standing long jump (SDS lms)	Researcher-administered CHECK test
																Sit-ups (SDS lms)	Researcher-administered CHECK test
																Stand and reach (SDS lms)	Researcher-administered CHECK test
																6 min run (SDS lms)	Researcher-administered CHECK test
																Dietary behaviour	Self-report questionnaire
																Dietary knowledge	Self-report questionnaire
																Daily physical activity	Objectively measured with accelerometry
																BMI	Objectively measured and calculated
																Muscle mass	Objectively measured using Bioimpedance analysis
																fat mass	Objectively measured using Bioimpedance analysis
																body fat percentage	Objectively measured using Bioimpedance analysis
Weber	2020	Exp Clin Endocrinol Dia	Germany	Controlled trial	Nutrition	166	9,3	0,7	52	139	9,9	0,8	50		Pre, post and 3 months	Nutrition-related skills	myself without any help"
																Nutrition Behavioural developmen	Series of questions on aspects of which the children pay attention to during a meal
																Nutrition Knowledge	Agreement on food related statements, and
																Nutrition Knowledge	Questions on recommended quantities of food and beverages
																Nutrition Knowledge	Creating a balanced breakfast
																Nutrition Attitudes	Series of questions on enjoyment of meal preparation and eating atmosphere
Wendel	2016	Am J Public Health	USA	RCT	Physical activity	62 (TT group)			47	49 (CC group)			51	2 years		BMI	objectively measured and
					Environment	59 (TC group)			51	Total groupmean age grade 2-3						BMI percentiles	calculated based on CDC
						23 (CT group)			57							weight status %	calculated based on CDC
Widhalm	2018	Obesity facts	Austria	Cluster RCT	Nutrition	82	11,69	0,67		59	12,04	0,59		1 yr	6, 12, 18 and 24 month	Weight	Objectively measured via bioimpedance analysis
					Other											body fat percentage	Objectively measured via bioimpedance analysis
																BMI SDS	calculated according to Must and Anderson
																Nutritional knowledge	Self-administered questionnaire (quiz)
																Weekly consumption of Fast food	determined retrospectively using the Food Frequency Questionnaire for children and adolescents
																Weekly consumption of Salty snac	determined retrospectively using the Food Frequency Questionnaire for children and adolescents
																Weekly consumption of Instant me	determined retrospectively using the Food Frequency Questionnaire for children and adolescents
																Weekly consumption of Sweets	determined retrospectively using the Food Frequency Questionnaire for children and adolescents
																Physical fitness	Objectively measured via Deutsche Motorik Test 6–18
																Motivation for physical activity	Phychological factors, Researcher administered interdisciplinary test system 'AD-EVA'
																Uncontrolled and unhealthy eating	Phychological factors, Researcher administered interdisciplinary test system 'AD-EVA'
																pre-clinical eating disorders	Phychological factors, Researcher administered interdisciplinary test system 'AD-EVA'
Williamson	2012	Obesity (Silver Spring)	USA	RCT	Nutrition	713 (Primary)	10,5	1,2	59	587	10,6	1,2	60	28 months		BMI z-score	calculated based on NHANES
					Physical activity	760 (Primary +										% body fat	Bioelectrical impedance
					Environment											Total energy intake (kCal)	Observation using photography
																Energy from total fat (kCal)	Observation using photography
																Energy from saturated fat (kcal)	Observation using photography
																Energy from protein (kCal)	Observation using photography
																Energy from carbohydrate (kcal)	Observation using photography
																PA time (min)	self-report SAPAC questionnaire
																SB time (min)	self-report SAPAC questionnaire
Williamson	2007	Obesity (Silver Spring)	USA	RCT	Nutrition	313				348				18 months		BMI z-score	calculated based on NHANES
					Physical activity					Total group 50% girlsmean age 9.2 (4.1)						% body fat	Bioelectrical impedance
																Total kilocalories, food selection	Observation using photography
																Total kilocalories, plate waste	Observation using photography
																Total kilocalories, food intake	Observation using photography
																Carbohydrate, food selection	Observation using photography
																Carbohydrate, plate waste	Observation using photography
																Carbohydrate, food intake	Observation using photography
																Protein, food selection	Observation using photography
																Protein, plate waste	Observation using photography
																Protein, food intake	Observation using photography
																Fat, food selection	Observation using photography
																Fat, plate waste	Observation using photography
																Fat, food intake	Observation using photography
																Saturated fat, food selection	Observation using photography
																Saturated fat, plate waste	Observation using photography
																Saturated fat, food intake	Observation using photography
																PA time (min)	self-report SAPAC + Godin-Shephard Leisure Time Physical Activity Questionnaire
																TV/Video (min)	self-report SAPAC
																Depression	self-report questionnaire
																Self-esteem	self-report questionnaire
																Eating disorder symptoms	self-report questionnaire
Wolfe, 2021	2014/15	Journal of School Healt	United States	Quasi-experimental	Nutrition	561	Not reported (	Not reported (3rd-(	48,5	No control	No control	No control	No control	~6 weeks	Baseline (~6 weeks bef	Overall dietary intake score	
					Physical activity											Vegetable intake score	
																Fruit intake score	
																food intake score	
																Milk intake score	
																Whole grains intake score	

																family	
																Facts labels	
																food intent to consume score	
																Tried new food	
																Asked family to buy vegetables	
																know' to a frequency	
																to a frequency	
Wright	2013	Int J Nurs Stud	USA	RCT	Nutrition	121	9	1,6	58	130	8,3	1,1	62	16 months		BMI	objectively measured and
Wright	2014	J Prev Interv Community			Physical activity											BMI z-score	calculted based on CDC growth
																Waist circumference	objectively measured
																Number of days with at least 60 m	self-report questionnaire
																Participation in team sports	self-report questionnaire
																Daily physical education class atter	self-report questionnaire
																TV viewing (h/day)	self-report questionnaire
																Computer/video game use(h/day)	self-report questionnaire
Wright	2012	Child Obes	USA	RCT	Nutrition	121	9	1,6	58	130	8,3	1,1	62	58 weeks		BMI	objectively measured and
					Physical activity											BMI z-score	calculated based on CDC
					Environment											French fries or chips intake	self-report questionnaire
																Vegetable intake	self-report questionnaire
																Beans intake	self-report questionnaire
																Fruit intake	self-report questionnaire
																100% Fruit juice intake	self-report questionnaire
																Sweets intake	self-report questionnaire
																Always reads nutrition labels	self-report questionnaire
																Always eats high-fiber cereal	self-report questionnaire
																Always eats whole-wheat bread	self-report questionnaire
																Always drinks 100% fruit juice	self-report questionnaire
																Always eats veggies for dinner	self-report questionnaire
																Food intentions	self-report questionnaire
																Food knowledge	self-report questionnaire
																Self-efficacy: Healthy food choices	self-report questionnaire
Xu	2015	Plos One	China	RCT	Nutrition	605	10,2	0,5	46	503	10,2	0,5	42	1 school year		Height (cm)	objectively measured
					Physical activity											Weight (kg)	objectively measured
																BMI	objectively measured and
																Weight status %	Calculated based on reference data for Chinese children by the Group of China Obesity Task Force
																Frequency jogging/running	self-report questionnaire
																Frequency of walking	self-report questionnaire
																Frequency of ball playing	self-report questionnaire
																Way of commuting to school	self-report questionnaire
																Frequency of screen time	self-report questionnaire
																Intake red meat	self-report questionnaire
																Intake fried snacks	self-report questionnaire
																Intake soft drinks	self-report questionnaire
																Intake vegetables	self-report questionnaire
Xu	2017	Asia Pacific journal of c	China	Cluster RCT	Nutrition	3773	9	1,4		3944	9	1,4		1 year	Bl, 1 year	BMI z scores	Calculated according to WHO growth references
																BMI	Objectively measured
																Being overweight (BMI between 8!	China (WGOC)
																Being obese (BMI above 95 th perc	China (WGOC)
																Being overweight and obese comb	China (WGOC)
																Waist circumference	Objectively measured
																Body composition	hand to foot bioelectrical impendence device
																Body fat percentage	Calculated using the prediction equations developed by Deurenberg, using data from bioelectrical impedance
																Serum glucose (mmol/L)	Determined by glucose-oxidase method
																Total cholesterol (mmol/L)	kits
																Insulin (mU/mL)	the morning after 10-14 h of overnight fasting.)
																Triglycerides (mmol/L)	kits
																low-density lipoprotein cholestero	kits
																high density lipoprotein cholesterc	kits
																Nutritional knowledge	Self-report questionnaire
																Nutritional attitude	Self-report questionnaire
																Nutritional practice	Self-report questionnaire
																Physical activity time	Self-report questionnaire
Xu	2020	Plos One	China	RCT	Nutrition	ive intervention)	9	1,2	51,8	1916 (control f	9	1,2	51,6	1 year (2 semesters)	Pre and post intervent	Overweight, obesity prevalence	objectively measured, China national standard
					Physical activity	:ducation group)	9,4	1,3	58	122 (control fo	9,1	1,4	42,6			Dietary diversity score, 9 food grou	self-report questionnaire
						303 (Physical Activity group)	9	1,3	53,5							Dietary diversity score, 28 food grc	self-report questionnaire
																Food variety score, 9 food groups (	self-report questionnaire
																DDS9 breakfast	self-report questionnaire
																DDS28 breakfast	self-report questionnaire
																FVS breakfast	self-report questionnaire
																DDS9 lunch	self-report questionnaire
																DDS28 lunch	self-report questionnaire
																FVS lunch	self-report questionnaire
																DDS9 supper	self-report questionnaire
																DDS28 supper	self-report questionnaire
																FVS supper	self-report questionnaire
																Cereal consumption, frequency	self-report questionnaire
																Meat consumption, frequency	self-report questionnaire
																Vegetables consumption, frequenc	self-report questionnaire
																Fruits consumption, frequency	self-report questionnaire
																Dairy consumption, frequency	self-report questionnaire
																Eggs consumption, frequency	self-report questionnaire
																Fish and shellfish consumption, fre	self-report questionnaire
																Fungi and algae consumption, freq	self-report questionnaire
																Dried legumes consumption, frequ	self-report questionnaire
																Nuts and seeds consumption, freq	self-report questionnaire
																Snack consumption, frequency	self-report questionnaire
																Fast foods consumption, frequenc	self-report questionnaire
																Beverages consumption, frequenc	self-report questionnaire
																Sugars and preserves consumptior	self-report questionnaire
Xu	2020	International Journal o	China	RCT	Nutrition	3431	9,1	1,4	49,3	3333	9,1	1,4	49,4	1 year (2 semesters)	Pre and post intervent	Energy expenditure	objectively measured
					Physical activity											Cereal consumption, frequency	self-report questionnaire
																Meat consumption, frequency	self-report questionnaire
																Vegetables consumption, frequenc	self-report questionnaire
																Fruits consumption, frequency	self-report questionnaire

																Dairy consumption, frequency	self-report questionnaire
																Eggs consumption, frequency	self-report questionnaire
																Fish and shellfish consumption, fre	self-report questionnaire
																Fungi and algae consumption, freq	self-report questionnaire
																Dried legumes consumption, frequ	self-report questionnaire
																Nuts and seeds consumption, freq	self-report questionnaire
																Snack consumption, frequency	self-report questionnaire
																Fast foods consumption, frequenc	self-report questionnaire
																Beverages consumption, frequenc	self-report questionnaire
																Sugars and preserves consumptior	self-report questionnaire
																Diastolic blood pressure, mmHg	objectively measured
																Systolic blood pressure, mmHg	objectively measured
																Incidence of high blood pressure	objectively measured, P95 cutoff
Yang	2017	International journal o	Korea	experimental study	Environment	418	10,9	1,6		350	11,0	1,5		1 year	Bl, 1 year	BMI	Objectively measured and calculated
																BMI z-score	Calculated based on the 2007 Korean standard growth table.
																BMI percentile	Calculated based on the 2007 Korean standard growth table.
																Waist circumference (cm)	Objectively measured
																WHtR	Objectively measured
																Body fat percentage	Objectively measured with bioelectrical impedance analysis
																Fat mass (kg)	Objectively measured with bioelectrical impedance analysis
																Muscle mass (kg)	Objectively measured with bioelectrical impedance analysis
																SBP (mm Hg)	Objectively measured with a mercury sphygmomanometer
																DBP (mm HG)	Objectively measured with a mercury sphygmomanometer
																Cardiopulmonary endurance	students, respectively)
																Flexibility	Objectively measured via Upper-body bending
																Muscular strength	Objectively measured via grasping
																Agility	Objectively measured via a standing long jump
																Student health behaviors	Researcher administered student-questionnaire group-survey
																Physical activity habits score	Researcher administered student-questionnaire group-survey
																Dietary habits score - usual dietary	Researcher administered student-questionnaire group-survey
																Dietary habits score - eating behav	Researcher administered student-questionnaire group-survey
Yin	2012	Child Obes	USA	RCT	Physical activity	292	8,7	0,6	53	284	8,7	0,5	52	3 years		Height (cm)	objectively measured
																Weight (kg)	objectively measured
																BMI z-score	?
																Waist circumference	objectively measured
																% body fat	measured with DXA
																Cardiorespiratory fitness (bpm)	measured (heart rate) after submaximal bench-stepping test
																Total Cholesterol/HDL ratio	non-fasting blood analyses
																Resting SBP mmHg	Objectively measured
																Resting DBP mmHg	Objectively measured
Yin	2005	Obesity research	USA	CT	Physical activity	312				289				3 years	Bl and 8 months	BMI (kg/m2)	Objectively measured
Gutin	2008	Int J Pediatr Obes			Nutrition		Total group: mean age: 8.7 (SD: 0.5); % girls: 52							(Year 1 = 8 months)		Body fat (%)	Objectively measured
					Other											Bone mineral density	Objectively measured
																Fat mass (kg)	Objectively measured
																Fat-free mass (kg)	Objectively measured
																Cardiovascular fitness (bpm)	Objectively measured, heart rate at completion of submaximal bench-stepping test
																Systolic blood pressure (mm Hg)	Objectively measured
																Diastolic blood pressure (mm Hg)	Objectively measured
																Total cholesterol (mg/dL)	Objectively measured
																HDL cholesterol (mg/dL)	Objectively measured
																Total cholesterol/HDL cholesterol	Calculated
Zammit	2018	International Journal o	Tunisia	RCT	Other	105	12,39	0,68		99	12,4	0,72		3 years	Bl, 3 year, 4 year	Tobacco-use	Self-administered questionnaire
																New experimentation with Tobacc	Self-administered questionnaire
																Participants who think that tobacc	Self-administered questionnaire
																Participants who think that tobacc	Self-administered questionnaire
																Sedentary on school days	Self-administered questionnaire
																Sedentary on sundays	Self-administered questionnaire
																Practice of recommended physical	Self-administered questionnaire
																Recommended amount of fruit an	Self-administered questionnaire
																Fried food intake (rarely or never)	Self-administered questionnaire
																Fast food intake (never in past wee	Self-administered questionnaire
																BMI	adolescents that pass through a BMI of 25 kg/m2 at age 18
																Weight excess (proportion of parti	Objectively measured (categorized in normal weight and excess weight)
Zhou	2020	Journal of Public Healt	China	RCT	Nutrition	612	10,2	0,5	46	513	10,2	0,5	42	1 year	Pre and post	Obesity prevalence	Objectively measureed; working group obesity in China (cut-offs)
					Physical activity											Obesity incidence (new cases)	Objectively measureed; working group obesity in China (cut-offs)
					Environment											Obesity remission	Objectively measureed; working group obesity in China (cut-offs)
																Height	Objectively measureed
																Weight	Objectively measureed
																BMI	Objectively measureed
																BMI z	Objectively measureed
																Red/white meat consumption	Self report questionnaire
																Vegetables consumption	Self report questionnaire
																Running time	Self report questionnaire
																Play ball time	Self report questionnaire
																Screen time	Self report questionnaire
																Awareness of fatty meat consump	Self report questionnaire
																Awareness of frequent fried snack	Self report questionnaire
																Awareness of frequent soft drinks	Self report questionnaire
																Awareness of shorten time spent i	Self report questionnaire
																Awareness of prolonged TV tiome	Self report questionnaire

Supplementary File 3. Reference list of studies included in the scoping review

1. Adab P, Pallan MJ, Cade J, Ekelund U, Barrett T, Daley A, et al. Preventing childhood obesity, phase II feasibility study focusing on South Asians: BEACHeS. BMJ Open. 2014;4(4):e004579.
2. Akdemir M, Donmez L, Polat H. The Effect of Nutritional and Physical Activity Interventions on Nutritional Status and Obesity in Primary School Children: A Cluster Randomized Controlled Study. Kuwait Med J. 2017;49(2):105–13.
3. Alexander AG, Grant WL, Pedrino KJ, Lyons PE. A prospective multifactorial intervention on subpopulations of predominately Hispanic children at high risk for obesity. Obesity (Silver Spring). 2014;22(1):249–53.
4. Allafi AR, Author A, Department of Food Science a, Nutrition CoLSKUPOB, Safat K, Correspondence A, et al. Effects of rewards and pedometer-feedback on children's physical activity: A school-based intervention study. Progress in Nutrition. 2020;22(1):122–6.
5. Almas A, Islam M, Jafar TH. School-based physical activity programme in preadolescent girls (9-11 years): a feasibility trial in Karachi, Pakistan. Arch Dis Child. 2013;98(7):515–9.
6. Angelopoulos PD, Milionis HJ, Grammatikaki E, Moschonis G, Manios Y. Changes in BMI and blood pressure after a school based intervention: the CHILDREN study. Eur J Public Health. 2009;19(3):319–25.
7. Ariza C, Sánchez-Martínez F, Serral G, Valmayor S, Juárez O, Pasarín M, et al. The Incidence of Obesity, Assessed as Adiposity, Is Reduced After 1 Year in Primary Schoolchildren by the POIBA Intervention. Journal of nutrition [Internet]. 2019; 149(2):[258–69 pp.]. Available from: https://www.cochranelibrary.com/central/doi/10.1002/central/CN-02000017/full.
8. Arlinghaus KR, Moreno JP, Reesor L, Hernandez DC, Johnston CA. Companeros: High School Students Mentor Middle School Students to Address Obesity Among Hispanic Adolescents. Prev Chronic Dis. 2017;14:E92.
9. Bacardi-Gascon M, Perez-Morales ME, Jimenez-Cruz A. A six month randomized school intervention and an 18-month follow-up intervention to prevent childhood obesity in Mexican elementary schools. Nutr Hosp. 2012;27(3):755–62.
10. Banchonhattakit P, Tanasugarn C, Pradipasen M, Miner KR, Nityasuddhi D. Effectiveness of School Network for Childhood Obesity Prevention (Snocop) in Primary Schools of Saraburi Province, Thailand. Se Asian J Trop Med. 2009;40(4):816–34.
11. Barnes C, Hall A, Nathan N, Sutherland R, McCarthy N, Pettet M, et al. Efficacy of a school-based physical activity and nutrition intervention on child weight status: findings from a cluster randomized controlled trial. Preventive medicine [Internet]. 2021; 153:[106822 p.]. Available from: https://www.cochranelibrary.com/central/doi/10.1002/central/CN-02336207/full.
12. Bartelink NHM, van Assema P, Kremers SPJ, Savelberg H, Oosterhoff M, Willeboordse M, et al. Can the Healthy Primary School of the Future offer perspective in the ongoing obesity epidemic in young children? A Dutch quasi-experimental study. BMJ Open. 2019;9(10):e030676.
13. Bilic-Kirin V, Burazin J, Buljan V, Milicic V, Gmajnic R. Influence of Health Education Regarding Correct Diet on Anthropometric Indexes in Children. Collegium Antropol. 2013;37(4):1089–94.
14. Bilinska I, Kryst L. Effectiveness of a school-based intervention to reduce the prevalence of overweight and obesity in children aged 7-11 years from Poznan (Poland). Anthropol Anz. 2017;74(2):89–100.
15. Brandstetter S, Klenk J, Berg S, Galm C, Fritz M, Peter R, et al. Overweight prevention implemented by primary school teachers: a randomised controlled trial. Obes Facts. 2012;5(1):1–11.
16. Breheny K, Passmore S, Adab P, Martin J, Hemming K, Lancashire E, et al. Effectiveness and cost-effectiveness of The Daily Mile on childhood weight outcomes and wellbeing: a cluster randomised controlled trial. International journal of obesity (2005) [Internet]. 2020; 44(4):[812–22 pp.]. Available from: https://www.cochranelibrary.com/central/doi/10.1002/central/CN-02076208/full.

17. Breslin G, Brennan D, Rafferty R, Gallagher AM, Hanna D. The effect of a healthy lifestyle programme on 8-9 year olds from social disadvantage. Arch Dis Child. 2012;97(7):618–24.
18. Bundy A, Engelen L, Wyver S, Tranter P, Ragen J, Bauman A, et al. Sydney Playground Project: A Cluster-Randomized Trial to Increase Physical Activity, Play, and Social Skills. J School Health. 2017;87(10):751–9.
19. Burgermaster M, Gray HL, Tipton E, Contento I, Koch P. Testing an Integrated Model of Program Implementation: the Food, Health & Choices School-Based Childhood Obesity Prevention Intervention Process Evaluation. Prev Sci. 2017;18(1):71–82.
20. Busch V, Steenkamer I, van Nassau F, van Opdorp P, van Houtum L, Verhoeff A, et al. The Effects of the Jump-In Whole-School Intervention on the Weight Development of Children in Amsterdam, the Netherlands. J Sch Health. 2024;94(1):37–46.
21. Caballero B, Clay T, Davis SM, Ethelbah B, Rock BH, Lohman T, et al. Pathways: a school-based, randomized controlled trial for the prevention of obesity in American Indian schoolchildren. American Journal of Clinical Nutrition. 2003;78(5):1030–8.
22. Canaway A, Frew E, Lancashire E, Pallan M, Hemming K, Adab P. Economic evaluation of a childhood obesity prevention programme for children: Results from the WAVES cluster randomised controlled trial conducted in schools. PLoS One. 2019;14(7):e0219500.
23. Cao Z, Hua J, Zhang D, Thapa J, Wang S. A cohort study assessing the sustainable long-term effectiveness of a childhood-obesity intervention in China. International journal of epidemiology [Internet]. 2019; 48(1):[108–15 pp.]. Available from: https://www.cochranelibrary.com/central/doi/10.1002/central/CN-02078605/full.
24. Cao ZJ, Wang SM, Chen Y. A randomized trial of multiple interventions for childhood obesity in China. Am J Prev Med. 2015;48(5):552–60.
25. Centeio EE, McCaughtry N, Moore EWG, Kulik N, Garn A, Martin J, et al. Building healthy communities: A comprehensive school health program to prevent obesity in elementary schools. Prev Med. 2018;111:210–5.
26. Centis E, Marzocchi R, Di Luzio R, Moscatiello S, Salardi S, Villanova N, et al. A controlled, class-based multicomponent intervention to promote healthy lifestyle and to reduce the burden of childhood obesity. Pediatr Obes. 2012;7(6):436–45.
27. Chawla N, Panza A, Sirikulchayanonta C, Kumar R, Taneepanichskul S. Effectiveness Of A School-Based Multicomponent Intervention On Nutritional Status Among Primary School Children In Bangkok, Thailand. J Ayub Med Coll Abbottabad. 2017;29(1):13–20.
28. Chesham RA, Booth JN, Sweeney EL, Ryde GC, Gorely T, Brooks NE, et al. The Daily Mile makes primary school children more active, less sedentary and improves their fitness and body composition: a quasi-experimental pilot study. BMC Med. 2018;16(1):64.
29. Cohen JF, Kraak VI, Choumenkovitch SF, Hyatt RR, Economos CD. The CHANGE study: a healthy-lifestyles intervention to improve rural children's diet quality. J Acad Nutr Diet. 2014;114(1):48–53.
30. Coknaz D, Mirzeoglu AD, Atasoy HI, Alkoy S, Coknaz H, Goral K. A digital movement in the world of inactive children: favourable outcomes of playing active video games in a pilot randomized trial. Eur J Pediatr. 2019;178(10):1567–76.
31. Cunha DB, de Souza Bda S, Pereira RA, Sichieri R. Effectiveness of a randomized school-based intervention involving families and teachers to prevent excessive weight gain among adolescents in Brazil. PLoS One. 2013;8(2):e57498.
32. da Silva LS, Fisberg M, de Souza Pires MM, Nassar SM, Sottovia CB. The effectiveness of a physical activity and nutrition education program in the prevention of overweight in schoolchildren in Criciuma, Brazil. Eur J Clin Nutr. 2013;67(11):1200–4.
33. Dagger RM, Davies IG, Mackintosh KA, Stone GL, George KP, Fairclough SJ, et al. The CHANGE! Project: Changes in Body Composition and Cardiorespiratory Fitness in 10- to 11-Year-Old Children After Completing the CHANGE! Intervention. Pediatr Exerc Sci. 2018;30(1):81–9.
34. Dalma A, Petralias A, Tsiampalis T, Nikolakopoulos S, Veloudaki A, Kastorini CM, et al. Effectiveness of a school food aid programme in improving household food insecurity; a cluster randomized trial. Eur J Public Health. 2020;30(1):171–8.

35. Dämon S, Dietrich S, Widhalm K. PRESTO—Prevention Study of Obesity: A project to prevent obesity during childhood and adolescence. Acta Paediatrica. 2005;94(0):47–8.
36. Danielzik S, Pust S, Muller MJ. School-based interventions to prevent overweight and obesity in prepubertal children: process and 4-years outcome evaluation of the Kiel Obesity Prevention Study (KOPS). Acta Paediatr. 2007;96(454):19–25.
37. Davis JN, Pérez A, Asigbee FM, Landry MJ, Vandyousefi S, Ghaddar R, et al. School-based gardening, cooking and nutrition intervention increased vegetable intake but did not reduce BMI: Texas sprouts - a cluster randomized controlled trial. Int J Behav Nutr Phys Act. 2021;18(1):18.
38. Davis S, Gomez Y, Lambert L, Skipper B. Primary prevention of obesity in American Indian children. Ann N Y Acad Sci. 1993;699:167–80.
39. de Greeff JW, Hartman E, Mullender-Wijnsma MJ, Bosker RJ, Doolaard S, Visscher C. Effect of Physically Active Academic Lessons on Body Mass Index and Physical Fitness in Primary School Children. J School Health. 2016;86(5):346–52.
40. De Henauw S, Huybrechts I, De Bourdeaudhuij I, Bammann K, Barba G, Lissner L, et al. Effects of a community-oriented obesity prevention programme on indicators of body fatness in preschool and primary school children. Main results from the IDEFICS study. Obes Rev. 2015;16 Suppl 2:16–29.
41. de Moraes MM, Mediano MFF, de Souza RAG, Moura AS, da Veiga GV, Sichieri R. Discouraging soft drink consumption reduces blood glucose and cholesterol of Brazilian elementary students: Secondary analysis of a randomized controlled trial. Prev Med. 2017;100:223–8.
42. de Ruyter JC, Olthof MR, Seidell JC, Katan MB. A trial of sugar-free or sugar-sweetened beverages and body weight in children. N Engl J Med. 2012;367(15):1397–406.
43. Delaney T, Wyse R, Yoong SL, Sutherland R, Wiggers J, Ball K, et al. Cluster randomized controlled trial of a consumer behavior intervention to improve healthy food purchases from online canteens. Am J Clin Nutr. 2017;106(5):1311–20.
44. Demir D, Bektas M. The Effect of an Obesity Prevention Program on Children's Eating Behaviors, Food Addiction, Physical Activity, and Obesity Status. J Pediatr Nurs. 2021;61:355–63.
45. DeVault N, Kennedy T, Hermann J, Mwavita M, Rask P, Jaworsky A. It's All About Kids: Preventing Overweight in Elementary School Children in Tulsa, OK. J Am Diet Assoc. 2009;109(4):680–7.
46. Di Maglie A, Marsigliante S, My G, Colazzo S, Muscella A. Effects of a physical activity intervention on schoolchildren fitness. Physiol Rep. 2022;10(2):e15115.
47. Diao H, Wang H, Yang L, Li T. The impacts of multiple obesity-related interventions on quality of life in children and adolescents: a randomized controlled trial. Health Qual Life Outcomes. 2020;18(1):213.
48. Dong Y, Zou Z, Wang H, Dong B, Hu P, Ma Y, et al. National School-Based Health Lifestyles Intervention in Chinese Children and Adolescents on Obesity and Hypertension. Front Pediatr. 2021;9:615283.
49. Donnelly JE, Greene JL, Gibson CA, Smith BK, Washburn RA, Sullivan DK, et al. Physical Activity Across the Curriculum (PAAC): a randomized controlled trial to promote physical activity and diminish overweight and obesity in elementary school children. Prev Med. 2009;49(4):336–41.
50. Donnelly JE, Jacobsen DJ, Whatley JE, Hill JO, Swift LL, Cherrington A, et al. Nutrition and physical activity program to attenuate obesity and promote physical and metabolic fitness in elementary school children. Obes Res. 1996;4(3):229–43.
51. Dring KJ, Hatch LM, Williams RA, Morris JG, Sunderland C, Nevill ME, et al. Effect of 5-weeks participation in The Daily Mile on cognitive function, physical fitness, and body composition in children. Sci Rep. 2022;12(1):14309.
52. Drummy C, Murtagh EM, McKee DP, Breslin G, Davison GW, Murphy MH. The effect of a classroom activity break on physical activity levels and adiposity in primary school children. J Paediatr Child Health. 2016;52(7):745–9.

53. El Harake MD, Kharroubi S, Hamadeh SK, Jomaa L. Impact of a Pilot School-Based Nutrition Intervention on Dietary Knowledge, Attitudes, Behavior and Nutritional Status of Syrian Refugee Children in the Bekaa, Lebanon. Nutrients. 2018;10(7).
54. Elder JP, Holub CK, Arredondo EM, Sánchez-Romero LM, Moreno-Saracho JE, Barquera S, et al. Promotion of water consumption in elementary school children in San Diego, USA and Tlaltizapan, Mexico. Salud Publica Mexico. 2014;56:S148–S56.
55. Elinder LS, Heinemans N, Hagberg J, Quetel AK, Hagströmer M. A participatory and capacity-building approach to healthy eating and physical activity - SCIP-school: a 2-year controlled trial. Int J Behav Nutr Phy. 2012;9.
56. Fairclough SJ, Hackett AF, Davies IG, Gobbi R, Mackintosh KA, Warburton GL, et al. Promoting healthy weight in primary school children through physical activity and nutrition education: a pragmatic evaluation of the CHANGE! randomised intervention study. Bmc Public Health. 2013;13.
57. Farmer VL, Williams SM, Mann JI, Schofield G, McPhee JC, Taylor RW. The effect of increasing risk and challenge in the school playground on physical activity and weight in children: a cluster randomised controlled trial (PLAY). Int J Obes (Lond). 2017;41(5):793–800.
58. Fetter DS, Scherr RE, Linnell JD, Dharmar M, Schaefer SE, Zidenberg-Cherr S. Effect of the Shaping Healthy Choices Program, a Multicomponent, School-Based Nutrition Intervention, on Physical Activity Intensity. J Am Coll Nutr. 2018;37(6):472–8.
59. Ford PA, Perkins G, Swaine I. Effects of a 15-week accumulated brisk walking programme on the body composition of primary school children. J Sports Sci. 2013;31(2):114–22.
60. Franckle RL, Falbe J, Gortmaker S, Barrett JL, Giles C, Ganter C, et al. Student obesity prevalence and behavioral outcomes for the massachusetts childhood obesity research demonstration project. Obesity (Silver Spring). 2017;25(7):1175–82.
61. Fung C, Kuhle S, Lu C, Purcell M, Schwartz M, Storey K, et al. From "best practice" to "next practice": the effectiveness of school-based health promotion in improving healthy eating and physical activity and preventing childhood obesity. Int J Behav Nutr Phy. 2012;9.
62. Gao Z, Pope Z, Lee JE, Stodden D, Roncesvalles N, Pasco D, et al. Impact of exergaming on young children's school day energy expenditure and moderate-to-vigorous physical activity levels. J Sport Health Sci. 2017;6(1):11–6.
63. Gatto NM, Martinez LC, Spruijt-Metz D, Davis JN. LA sprouts randomized controlled nutrition, cooking and gardening programme reduces obesity and metabolic risk in Hispanic/Latino youth. Pediatr Obes. 2017;12(1):28–37.
64. Gold A, Larson M, Tucker J, Strang M. Classroom Nutrition Education Combined With Fruit and Vegetable Taste Testing Improves Children's Dietary Intake. J School Health. 2017;87(2):106–13.
65. Goswami N, Trozic I, Fredriksen MV, Fredriksen PM. The effect of physical activity intervention and nutritional habits on anthropometric measures in elementary school children: the health oriented pedagogical project (HOPP). Int J Obes (Lond). 2021;45(8):1677–86.
66. Graf C, Koch B, Falkowski G, Jouck S, Christ H, Staudenmaier K, et al. School-based prevention: effects on obesity and physical performance after 4 years. J Sports Sci. 2008;26(10):987–94.
67. Graf C, Koch B, Falkowski G, Jouck S, Christ H, Stauenmaier K, et al. Effects of a school-based intervention on BMI and motor abilities in childhood. J Sport Sci Med. 2005;4(3):291–9.
68. Grao-Cruces A, Ruiz-López R, Moral-García JE, Ruiz-Ariza A, Martínez-López EJ. Effects of a Steps/Day Programme with Evaluation in Physical Education on Body Mass Index in Schoolchildren 11-12 Years of Age. Kinesiology. 2016;48(1):132–41.
69. Grassi E, Evans A, Ranjit N, Pria SD, Messina L. Using a mixed-methods approach to measure impact of a school-based nutrition and media education intervention study on fruit and vegetable intake of Italian children. Public Health Nutr. 2016;19(11):1952–63.
70. Greene KN, Gabrielyan G, Just DR, Wansink B. Fruit-Promoting Smarter Lunchrooms Interventions: Results From a Cluster RCT. Am J Prev Med. 2017;52(4):451–8.
71. Grydeland M, Bjelland M, Anderssen SA, Klepp KI, Bergh IH, Andersen LF, et al. Effects of a 20-month cluster randomised controlled school-based intervention trial on BMI of school-aged boys and girls: the HEIA study. Br J Sports Med. 2014;48(9):768–73.

72. Gutierrez-Martinez L, Martinez RG, Gonzalez SA, Bolivar MA, Estupinan OV, Sarmiento OL. Effects of a strategy for the promotion of physical activity in students from Bogota. Rev Saude Publica. 2018;52:79.
73. Habib-Mourad C, Ghandour LA, Maliha C, Awada N, Dagher M, Hwalla N. Impact of a one-year school-based teacher-implemented nutrition and physical activity intervention: main findings and future recommendations. Bmc Public Health. 2020;20(1).
74. Habib-Mourad C, Ghandour LA, Moore HJ, Nabhani-Zeidan M, Adetayo K, Hwalla N, et al. Promoting healthy eating and physical activity among school children: findings from Health-E-PALS, the first pilot intervention from Lebanon. Bmc Public Health. 2014;14.
75. Habib-Mourad C, Maliha C, Kassis A, Nguyen AT, Ammar D, Haji E, et al. A randomised controlled school-based nutritional intervention in five Middle Eastern countries: Ajyal Salima improved students' dietary and physical activity habits. Public Health Nutr. 2023;26(10):2036–47.
76. Habib-Mourad CGLAMCDMKSHN, Author A, Department of N, Food Sciences Fo, Agriculture, Food Sciences AUoBB, et al. Impact of a three-year obesity prevention study on healthy behaviors and bmi among lebanese schoolchildren: Findings from ajyal salima program. Nutrients. 2020;12(9):1–14.
77. Hadi AJ, Hadju V, Suriah, Indriasari R, Manggabara S, Yetti RE, et al. The Effect of the Implementation of a Planned Peer Group Session Model on Obesity Prevention Among Students of an Integrated Islamic Primary School in Makassar. Pakistan Journal of Nutrition. 2019;18(9):882–7.
78. Harrison M, Burns CF, McGuinness M, Heslin J, Murphy NM. Influence of a health education intervention on physical activity and screen time in primary school children: 'Switch Off--Get Active'. J Sci Med Sport. 2006;9(5):388–94.
79. Hawkins KR, Burton JH, Apolzan JW, Thomson JL, Williamson DA, Martin CK. Efficacy of a school-based obesity prevention intervention at reducing added sugar and sodium in children's school lunches: the LA Health randomized controlled trial. Int J Obes (Lond). 2018;42(11):1845–52.
80. Hendy HM, Williams KE, Camise TS. Kid's Choice Program improves weight management behaviors and weight status in school children. Appetite. 2011;56(2):484–94.
81. Herscovici CR, Kovalskys I, De Gregorio MJ. Gender differences and a school-based obesity prevention program in Argentina: a randomized trial. Rev Panam Salud Publ. 2013;34(2):75–82.
82. Hollar D, Lombardo M, Lopez-Mitnik G, Hollar TL, Almon M, Agatston AS, et al. Effective multi-level, multi-sector, school-based obesity prevention programming improves weight, blood pressure, and academic performance, especially among low-income, minority children. J Health Care Poor Underserved. 2010;21(2 Suppl):93–108.
83. Hollar D, Messiah SE, Lopez-Mitnik G, Hollar TL, Almon M, Agatston AS. Effect of a two-year obesity prevention intervention on percentile changes in body mass index and academic performance in low-income elementary school children. Am J Public Health. 2010;100(4):646–53.
84. Hollar D, Messiah SE, Lopez-Mitnik G, Hollar TL, Almon M, Agatston AS. Healthier Options for Public Schoolchildren Program Improves Weight and Blood Pressure in 6-to 13-Year-Olds. J Am Diet Assoc. 2010;110(2):261–7.
85. Hopkins LC, Holloman C, Melnyk B, Fristad M, Goodway JD, Kennel JA, et al. Participation in structured programming may prevent unhealthy weight gain during the summer in school-aged children from low-income neighbourhoods: feasibility, fidelity and preliminary efficacy findings from the Camp NERF study. Public Health Nutr. 2019;22(6):1100–12.
86. Howe CA, Freedson PS, Alhassan S, Feldman HA, Osganian SK. A recess intervention to promote moderate-to-vigorous physical activity. Pediatr Obes. 2012;7(1):82–8.
87. Hrafnkelsson H, Magnusson KT, Thorsdottir I, Johannsson E, Sigurdsson EL. Result of school-based intervention on cardiovascular risk factors. Scand J Prim Health Care. 2014;32(4):149–55.
88. Huang C, Dannenberg AL, Haaland W, Mendoza JA. Changes in Self-Efficacy and Outcome Expectations From Child Participation in Bicycle Trains for Commuting to and From School. Health Educ Behav. 2018;45(5):748–55.

89. Huberty JL, Beets MW, Beighle A, Welk G. Environmental Modifications to Increase Physical Activity During Recess: Preliminary Findings From Ready for Recess. J Phys Act Health. 2011;8:S249–S56.
90. Ickovics JR, Duffany KO, Shebl FM, Peters SM, Read MA, Gilstad-Hayden KR, et al. Implementing School-Based Policies to Prevent Obesity: Cluster Randomized Trial. Am J Prev Med. 2019;56(1):e1–e11.
91. Idamokoro M, Pienaar AE, Gerber B, van Gent MM. The Influence of a 9-Week Movement Program on the Body Composition of 7- to 8-Year-Old Schoolchildren in the Eastern Cape of South Africa. Int J Environ Res Public Health. 2023;20(3).
92. Jakubowski TL, Perron T, Farrell A, Kenner C, Hullings C. The Smart Nutrition and Conditioning for Kids (SNACK) Program. Mcn-Am J Matern-Chil. 2018;43(5):278–84.
93. James J, Thomas P, Cavan D, Kerr D. Preventing childhood obesity by reducing consumption of carbonated drinks: cluster randomised controlled trial. BMJ. 2004;328(7450):1237.
94. James J, Thomas P, Kerr D. Preventing childhood obesity: two year follow-up results from the Christchurch obesity prevention programme in schools (CHOPPS). BMJ. 2007;335(7623):762.
95. Jansen W, Borsboom G, Meima A, Zwanenburg EJ, Mackenbach JP, Raat H, et al. Effectiveness of a primary school-based intervention to reduce overweight. Int J Pediatr Obes. 2011;6(2-2):e70–7.
96. Jiang J, Xia X, Greiner T, Wu G, Lian G, Rosenqvist U. The effects of a 3-year obesity intervention in schoolchildren in Beijing. Child Care Health Dev. 2007;33(5):641–6.
97. Johnston CA, Moreno JP, El-Mubasher A, Gallagher M, Tyler C, Woehle D. Impact of a School-Based Pediatric Obesity Prevention Program Facilitated by Health Professionals. J School Health. 2013;83(3):171–81.
98. Juric P, Dudley DA, Petocz P. Does incorporating high intensity interval training in physical education classes improve fitness outcomes of students? A cluster randomized controlled trial. Prev Med Rep. 2023;32:102127.
99. Kain J, Concha F, Moreno L, Leyton B. School-based obesity prevention intervention in Chilean children: effective in controlling, but not reducing obesity. J Obes. 2014;2014:618293.
100. Kain J, Leyton B, Cerda R, Vio F, Uauy R. Two-year controlled effectiveness trial of a school-based intervention to prevent obesity in Chilean children. Public Health Nutr. 2009;12(9):1451–61.
101. Kamin T, Koroušić Seljak B, Fidler Mis N. Water Wins, Communication Matters: School-Based Intervention to Reduce Intake of Sugar-Sweetened Beverages and Increase Intake of Water. Nutrients. 2022;14(7).
102. Karczewski SA, Carter JS, DeCator DD. The Role of Ethnicity in School-Based Obesity Intervention for School-Aged Children: A Pilot Evaluation. J School Health. 2016;86(11):778–86.
103. Khan M, Bell R. Effects of a School Based Intervention on Children's Physical Activity and Healthy Eating: A Mixed-Methods Study. Int J Environ Res Public Health. 2019;16(22).
104. Kipping RR, Payne C, Lawlor DA. Randomised controlled trial adapting US school obesity prevention to England. Arch Dis Child. 2008;93(6):469–73.
105. Klakk H, Chinapaw M, Heidemann M, Andersen LB, Wedderkopp N. Effect of four additional physical education lessons on body composition in children aged 8-13 years - a prospective study during two school years. Bmc Pediatr. 2013;13.
106. Klish WJ, Karavias KE, White KS, Balch AJ, Fraley JK, Mikhail C, et al. Multicomponent school-initiated obesity intervention in a high-risk, Hispanic elementary school. J Pediatr Gastroenterol Nutr. 2012;54(1):113–6.
107. Kobel S, Lammle C, Wartha O, Kesztyus D, Wirt T, Steinacker JM. Effects of a Randomised Controlled School-Based Health Promotion Intervention on Obesity Related Behavioural Outcomes of Children with Migration Background. J Immigr Minor Health. 2017;19(2):254–62.
108. Koch PA, Contento IR, Gray HL, Burgermaster M, Bandelli L, Abrams E, et al. Food, Health, & Choices: Curriculum and Wellness Interventions to Decrease Childhood Obesity in Fifth-Graders. J Nutr Educ Behav. 2019;51(4):440–55.

109. Kocken PL, Scholten AM, Westhoff E, De Kok BP, Taal EM, Goldbohm RA. Effects of a Theory-Based Education Program to Prevent Overweightness in Primary School Children. Nutrients. 2016;8(1).
110. Kriemler S, Zahner L, Schindler C, Meyer U, Hartmann T, Hebestreit H, et al. Effect of school based physical activity programme (KISS) on fitness and adiposity in primary schoolchildren: cluster randomised controlled trial. BMJ. 2010;340:c785.
111. Kühr P, Lima RA, Grøntved A, Wedderkopp N, Klakk H. Three times as much physical education reduced the risk of children being overweight or obese after 5 years. Acta Paediatrica. 2020;109(3):595–601.
112. Lambrick D, Westrupp N, Kaufmann S, Stoner L, Faulkner J. The effectiveness of a high-intensity games intervention on improving indices of health in young children. J Sports Sci. 2016;34(3):190–8.
113. Larson JN, Brusseau TA, Wengreen H, Fairclough SJ, Newton MM, Hannon JC. Fit "N" Cool Kids: The Effects of Character Modeling and Goal Setting on Children's Physical Activity and Fruit and Vegetable Consumption. Clin Med Insights Pediatr. 2018;12:1179556518784296.
114. Lazaar N, Aucouturier J, Ratel S, Rance M, Meyer M, Duche P. Effect of physical activity intervention on body composition in young children: influence of body mass index status and gender. Acta Paediatr. 2007;96(9):1315–20.
115. Lee GY, Choi YJ. Effects of an obesity management mentoring program for Korean children. Appl Nurs Res. 2016;31:160–4.
116. Leuven J, Rutenfrans AHM, Dolfing AG, Leuven R. School gardening increases knowledge of primary school children on edible plants and preference for vegetables. Food Sci Nutr. 2018;6(7):1960–7.
117. Li B, Pallan M, Liu WJ, Hemming K, Frew E, Lin R, et al. The CHIRPY DRAGON intervention in preventing obesity in Chinese primary-school--aged children: A cluster-randomised controlled trial. PLoS Med. 2019;16(11):e1002971.
118. Li XH, Lin S, Guo H, Huang Y, Wu L, Zhang Z, et al. Effectiveness of a school-based physical activity intervention on obesity in school children: a nonrandomized controlled trial. BMC Public Health. 2014;14:1282.
119. Lin YC, Chen HJ, Wang Y, Min J, Wu HC, Carvajal NA, et al. NASA Mission X Program for Healthy Eating and Active Living among Taiwanese Elementary School Students. J Pediatr Nurs. 2019;49:e8–e14.
120. Liu A, Hu X, Ma G, Cui Z, Pan Y, Chang S, et al. Evaluation of a classroom-based physical activity promoting programme. Obesity Reviews. 2008;9:130–4.
121. Liu AL, Hu XQ, Ma GS, Cui ZH, Pan YP, Chang SY, et al. Report on childhood obesity in China (6) Evaluation of a classroom-based physical activity promotion program. Biomed Environ Sci. 2007;20(1):19–23.
122. Liu Z, Gao P, Gao AY, Lin Y, Feng XX, Zhang F, et al. Effectiveness of a Multifaceted Intervention for Prevention of Obesity in Primary School Children in China: A Cluster Randomized Clinical Trial. JAMA Pediatr. 2022;176(1):e214375.
123. Liu Z, Li Q, Maddison R, Ni Mhurchu C, Jiang Y, Wei DM, et al. A School-Based Comprehensive Intervention for Childhood Obesity in China: A Cluster Randomized Controlled Trial. Child Obes. 2019;15(2):105–15.
124. Llargues E, Franco R, Recasens A, Nadal A, Vila M, Perez MJ, et al. Assessment of a school-based intervention in eating habits and physical activity in school children: the AVall study. J Epidemiol Community Health. 2011;65(10):896–901.
125. Llargues E, Recasens MA, Manresa JM, Jensen BB, Franco R, Nadal A, et al. Four-year outcomes of an educational intervention in healthy habits in schoolchildren: the Avall 3 Trial. Eur J Public Health. 2017;27(1):42–7.
126. Llaurado E, Pedret A, Valls RM, Sola R, Tarro L. A 16-month follow-up after a youth-led social marketing intervention to encourage healthy lifestyles in children (aged 9 at baseline and 11 at follow-up) from disadvantaged neighbourhoods: the European Youth Tackling Obesity-Kids project. Public Health. 2024;232:1–3.

127. Lloyd J, Creanor S, Logan S, Green C, Dean SG, Hillsdon M, et al. Effectiveness of the Healthy Lifestyles Programme (HeLP) to prevent obesity in UK primary-school children: a cluster randomised controlled trial. Lancet Child Adolesc Health. 2018;2(1):35–45.
128. Lloyd J, Creanor S, Price L, Abraham C, Dean S, Green C, et al. Trial baseline characteristics of a cluster randomised controlled trial of a school-located obesity prevention programme; the Healthy Lifestyles Programme (HeLP) trial. BMC Public Health. 2017;17(1):291.
129. Lloyd J, McHugh C, Minton J, Eke H, Wyatt K. The impact of active stakeholder involvement on recruitment, retention and engagement of schools, children and their families in the cluster randomised controlled trial of the Healthy Lifestyles Programme (HeLP): a school-based intervention to prevent obesity. Trials. 2017;18(1):378.
130. Lloyd JJ, Wyatt KM, Creanor S. Behavioural and weight status outcomes from an exploratory trial of the Healthy Lifestyles Programme (HeLP): a novel school-based obesity prevention programme. BMJ Open. 2012;2(3).
131. Long K, Beckmann J, Lang C, Seelig H, Nqweniso S, Probst-Hensch N, et al. Impact of a school-based health intervention program on body composition among South African primary schoolchildren: results from the KaziAfya cluster-randomized controlled trial. BMC medicine [Internet]. 2022; 20(1):[27 p.]. Available from: https://www.cochranelibrary.com/central/doi/10.1002/central/CN-02364106/full.
132. Long KZ, Beckmann J, Lang C, Seelig H, Nqweniso S, Probst-Hensch N, et al. Randomized Trial to Improve Body Composition and Micronutrient Status Among South African Children. Am J Prev Med. 2024;66(6):1078–88.
133. Long MW, Weber MR, Allan MJ, Ma Y, Jin Y, Aldous A, et al. Evaluation of a pragmatic trial of a collaborative school-based obesity prevention intervention in a low-income urban district. Preventive Medicine: An International Journal Devoted to Practice and Theory. 2020;133:ArtID: 106020.
134. López Sánchez GF, Díaz Suárez A, Radzimiński Ł, Jastrzębski Z. Effects of a 12-week physical education program on the body composition of 10- and 11-year-old children. Science & Sports. 2017;32(4):e155–e61.
135. Lynch BA, Gentile N, Maxson J, Quigg S, Swenson L, Kaufman T. Elementary School-Based Obesity Intervention Using an Educational Curriculum. J Prim Care Community Health. 2016;7(4):265–71.
136. Lyu JL, Liu Z, Zhou S, Feng XX, Lin Y, Gao AY, et al. The Effect of a Multifaceted Intervention on Dietary Quality in Schoolchildren and the Mediating Effect of Dietary Quality between Intervention and Changes in Adiposity Indicators: A Cluster Randomized Controlled Trial. Nutrients. 2022;14(16).
137. Madsen KA, Linchey J, Ritchie L, Thompson HR. The Fit Study: Design and rationale for a cluster randomized trial of school-based BMI screening and reporting. Contemp Clin Trials. 2017;58:40–6.
138. Magnusson KT, Hrafnkelsson H, Sigurgeirsson I, Johannsson E, Sveinsson T. Limited effects of a 2-year school-based physical activity intervention on body composition and cardiorespiratory fitness in 7-year-old children. Health Educ Res. 2012;27(3):484–94.
139. Manley D, Cowan P, Graff C, Perlow M, Rice P, Richey P, et al. Self-efficacy, physical activity, and aerobic fitness in middle school children: examination of a pedometer intervention program. J Pediatr Nurs. 2014;29(3):228–37.
140. Marcus C, Nyberg G, Nordenfelt A, Karpmyr M, Kowalski J, Ekelund U. A 4-year, cluster-randomized, controlled childhood obesity prevention study: STOPP. Int J Obes (Lond). 2009;33(4):408–17.
141. Marsigliante S, Ciardo V, Di Maglie A, My G, Muscella A. Efficacy of school-based intervention programs in reducing overweight: A randomized trial. Front Nutr. 2022;9:1001934.
142. Marsigliante S, Gomez-Lopez M, Muscella A. Effects on Children's Physical and Mental Well-Being of a Physical-Activity-Based School Intervention Program: A Randomized Study. Int J Environ Res Public Health. 2023;20(3).
143. Martinez Vizcaino V, Salcedo Aguilar F, Franquelo Gutierrez R, Solera Martinez M, Sanchez Lopez M, Serrano Martinez S, et al. Assessment of an after-school physical activity program to

prevent obesity among 9- to 10-year-old children: a cluster randomized trial. Int J Obes (Lond). 2008;32(1):12–22.
144. Martinez-Vizcaino V, Sanchez-Lopez M, Notario-Pacheco B, Salcedo-Aguilar F, Solera-Martinez M, Franquelo-Morales P, et al. Gender differences on effectiveness of a school-based physical activity intervention for reducing cardiometabolic risk: a cluster randomized trial. Int J Behav Nutr Phys Act. 2014;11:154.
145. McMurray RG, Harrell JS, Bangdiwala SI, Bradley CB, Deng SB, Levine A. A school-based intervention can reduce body fat and blood pressure in young adolescents. J Adolescent Health. 2002;31(2):125–32.
146. McWhannell N, Henaghan JL, Foweather L, Doran DA, Batterham AM, Reilly T, et al. The effect of a 9-week physical activity programme on bone and body composition of children aged 10-11 years: an exploratory trial. Int J Sports Med. 2008;29(12):941–7.
147. Menor-Rodriguez MJ, Cortes-Martin J, Rodriguez-Blanque R, Tovar-Galvez MI, Aguilar-Cordero MJ, Sanchez-Garcia JC. Influence of an Educational Intervention on Eating Habits in School-Aged Children. Children (Basel). 2022;9(4).
148. Merrotsy A, McCarthy AL, Flack J, Lacey S, Coppinger T. Project Spraoi: a two-year longitudinal study on the effectiveness of a school-based nutrition and physical activity intervention on dietary intake, nutritional knowledge and markers of health of Irish schoolchildren. Public Health Nutr. 2019;22(13):2489–99.
149. Meyer U, Schindler C, Zahner L, Ernst D, Hebestreit H, van Mechelen W, et al. Long-term effect of a school-based physical activity program (KISS) on fitness and adiposity in children: a cluster-randomized controlled trial. PLoS One. 2014;9(2):e87929.
150. Mora T, Llargues E, Recasens A. Does health education affect BMI? Evidence from a school-based randomised-control trial. Econ Hum Biol. 2015;17:190–201.
151. Morgado MC, Sousa M, Coelho AB, Vale S, Costa JA, Seabra A. Effects of "Football and Nutrition for Health" program on body composition, physical fitness, eating behaviours, nutritional knowledge, and psychological status among 7 to 10 years school children. Front Pediatr. 2023;11:1251053.
152. Muckelbauer R, Libuda L, Clausen K, Reinehr T, Kersting M. A simple dietary intervention in the school setting decreased incidence of overweight in children. Obes Facts. 2009;2(5):282–5.
153. Muckelbauer R, Libuda L, Clausen K, Toschke AM, Reinehr T, Kersting M. Promotion and provision of drinking water in schools for overweight prevention: randomized, controlled cluster trial. Pediatrics. 2009;123(4):e661–7.
154. Muller I, Schindler C, Adams L, Endes K, Gall S, Gerber M, et al. Effect of a Multidimensional Physical Activity Intervention on Body Mass Index, Skinfolds and Fitness in South African Children: Results from a Cluster-Randomised Controlled Trial. Int J Environ Res Public Health. 2019;16(2).
155. Müller MJ, Asbeck I, Mast M, Langnäse K, Grund A. Prevention of obesity -: more than an intention.: Concept and first results of the Kiel Obesity Prevention Study (KOPS). Int J Obesity. 2001;25:S66–S74.
156. Muros JJ, Zabala M, Oliveras-López MJ, Ocana-Lara FA, de la Serra HLG. Results of a 7-Week School-Based Physical Activity and Nutrition Pilot Program on Health-Related Parameters in Primary School Children in Southern Spain. Pediatric Exercise Science. 2013;25(2):248–61.
157. Narayanan N, Nagpal N, Zieve H, Vyas A, Tatum J, Ramos M, et al. A School-Based Intervention Using Health Mentors to Address Childhood Obesity by Strengthening School Wellness Policy. Prev Chronic Dis. 2019;16:E154.
158. Ng CM, Kaur S, Koo HC, Mukhtar F, Yim HS. Experiential healthy meal preparation: A randomized-controlled trial to improve food group consumption and weight status among children. Human Nutrition & Metabolism. 2022;28.
159. Nickel NC, Doupe M, Enns JE, Brownell M, Sarkar J, Chateau D, et al. Differential effects of a school-based obesity prevention program: A cluster randomized trial. Matern Child Nutr. 2021;17(1):e13009.
160. Nogueira RC, Weeks BK, Beck B. One-Year Follow-up of the CAPO Kids Trial: Are Physical Benefits Maintained? Pediatr Exerc Sci. 2017;29(4):486–95.

161. Nogueira RC, Weeks BK, Beck BR. An in-school exercise intervention to enhance bone and reduce fat in girls: the CAPO Kids trial. Bone. 2014;68:92–9.
162. Nogueira RC, Weeks BK, Beck BR. Targeting bone and fat with novel exercise for peripubertal boys: the CAPO kids trial. Pediatr Exerc Sci. 2015;27(1):128–39.
163. Norman A, Malek ME, Nyberg G, Patterson E, Elinder LS. Effects of Universal School-Based Parental Support for Children's Healthy Diet and Physical Activity-the Healthy School Start Plus Cluster-Randomised Controlled Trial. Prev Sci. 2024;25(6):963–77.
164. Nqweniso SDRRALDJGSGMJNMISDSHSPP-HN, Author A, Department of Human Movement Science NMU, Port Elizabeth SA, Department of Sport E, and Health UoBS, et al. Effect of school-based interventions on body composition of grade-4 children from lower socioeconomic communities in Gqeberha, South Africa. SAJCH South African Journal of Child Health. 2021;15(2):89–98.
165. Olvera LGT, Martinez LC, Souza B, Sichieri R, Ruiz Arregui L, Rodriguez Garcia WD, et al. Impact of a School-Based Intervention on Sugar-Sweetened Beverage Intake Substitution by Water in Mexican Scholars. Ann Nutr Metab. 2024;80(3):136–42.
166. Paulsamy P, Periannan K, Easwaran V, Abdulla Khan N, Manoharan V, Venkatesan K, et al. School-Based Exercise and Life Style Motivation Intervention (SEAL.MI) on Adolescent's Cardiovascular Risk Factors and Academic Performance: Catch Them Young. Healthcare (Basel). 2021;9(11).
167. Peña S, Carranza M, Cuadrado C, Parra DC, Villalobos Dintrans P, Castillo C, et al. Effectiveness of a Gamification Strategy to Prevent Childhood Obesity in Schools: A Cluster Controlled Trial. Obesity (Silver Spring). 2021;29(11):1825–34.
168. Peralta LR, Jones RA, Okely AD. Promoting healthy lifestyles among adolescent boys: the Fitness Improvement and Lifestyle Awareness Program RCT. Prev Med. 2009;48(6):537–42.
169. Plachta-Danielzik S, Pust S, Asbeck I, Czerwinski-Mast M, Langnäse K, Fischer C, et al. Four-year follow-up of school-based intervention on overweight children:: The KOPS study. Obesity. 2007;15(12):3159–69.
170. Polman MAA, Beckers D, Burk WJ, Smit CR, Buijzen M, Vink JM, et al. The effect of a multi-component school-based social network intervention on children's body mass index: a four-arm intervention study. Psychol Health. 2024;39(11):1503–20.
171. Polonsky HM, Bauer KW, Fisher JO, Davey A, Sherman S, Abel ML, et al. Effect of a Breakfast in the Classroom Initiative on Obesity in Urban School-aged Children: A Cluster Randomized Clinical Trial. JAMA Pediatr. 2019;173(4):326–33.
172. Pongutta S, Ferguson E, Davey C, Tangcharoensathien V, Limwattananon S, Borghi J, et al. The impact of a complex school nutrition intervention on double burden of malnutrition among Thai primary school children: a 2-year quasi-experiment. Public Health. 2023;224:51–7.
173. Pope M. Preventing Weight Gain in Children Who Are School Age and African-American. Pediatr Phys Ther. 2016;28(2):207–16.
174. Qian L, Newman IM, Yuen LW, Du W, Shell DF. Effects of a comprehensive nutrition education programme to change grade 4 primary-school students' eating behaviours in China. Public Health Nutr. 2019;22(5):903–11.
175. Ramírez-Rivera D, Martínez-Contreras T, Villegas-Valle R, Henry-Mejia G, Quizán-Plata T, Haby M, et al. Preliminary Results of the Planet Nutrition Program on Obesity Parameters in Mexican Schoolchildren: pilot Single-School Randomized Controlled Trial. International journal of environmental research and public health [Internet]. 2021; 18(2). Available from: https://www.cochranelibrary.com/central/doi/10.1002/central/CN-02247570/full.
176. Recasens MA, Xicola-Coromina E, Manresa JM, Ullmo PA, Jensen BB, Franco R, et al. Impact of school-based nutrition and physical activity intervention on body mass index eight years after cessation of randomized controlled trial (AVall study). Clin Nutr. 2019;38(6):2592–8.
177. Robbins LB, Ling J, Clevenger K, Voskuil VR, Wasilevich E, Kerver JM, et al. A School- and Home-Based Intervention to Improve Adolescents' Physical Activity and Healthy Eating: A Pilot Study. J Sch Nurs. 2020;36(2):121–34.
178. Robbins LB, Ling J, Wen F. Attending After-School Physical Activity Club 2 Days a Week Attenuated an Increase in Percentage Body Fat and a Decrease in Fitness Among Adolescent Girls at Risk for Obesity. Am J Health Promot. 2020;34(5):500–4.

179. Robinson TN. Reducing children's television viewing to prevent obesity - A randomized controlled trial. Jama-J Am Med Assoc. 1999;282(16):1561–7.
180. Roccaldo R, Censi L, D'Addezio L, Berni Canani S, Gennaro L. A teachers' training program accompanying the "School Fruit Scheme" fruit distribution improves children's adherence to the Mediterranean diet: an Italian trial. Int J Food Sci Nutr. 2017;68(7):887–900.
181. Roccaldo R, Censi L, D'Addezio L, Berni Canani S, Gennaro L. Improvement of Adherence to the Mediterranean Diet through a Nutrition Education Teaching Pack for Teachers within the "School Fruit Scheme" Program: An Italian Long-Term Trial in School Children. Nutrients. 2024;16(13).
182. Ronca F, Burgess PW, Savage P, Senaratne N, Watson E, Loosemore M. Decreasing Sedentary Time during Lessons Reduces Obesity in Primary School Children: The Active Movement Study. Obes Facts. 2024;17(3):243–54.
183. Rosario R, Araujo A, Padrao P, Lopes O, Moreira A, Pereira B, et al. Health Promotion Intervention to Improve Diet Quality in Children. Health Promot Pract. 2017;18(2):253–62.
184. Rosario R, Oliveira B, Araujo A, Lopes O, Padrao P, Moreira A, et al. The impact of an intervention taught by trained teachers on childhood overweight. Int J Environ Res Public Health. 2012;9(4):1355–67.
185. Rosenkranz RR, Dixon PM, Dzewaltowski DA, McLoughlin GM, Lee JA, Chen S, et al. A cluster-randomized trial comparing two SWITCH implementation support strategies for school wellness intervention effectiveness. J Sport Health Sci. 2023;12(1):87–96.
186. Rush E, Reed P, McLennan S, Coppinger T, Simmons D, Graham D. A school-based obesity control programme: Project Energize. Two-year outcomes. Br J Nutr. 2012;107(4):581–7.
187. Sadeghi B, Kaiser LL, Hanbury MM, Tseregounis IE, Shaikh U, Gomez-Camacho R, et al. A three-year multifaceted intervention to prevent obesity in children of Mexican-heritage. BMC Public Health. 2019;19(1):582.
188. Safdie M, Jennings-Aburto N, Lévesque L, Janssen I, Campirano-Núñez F, López-Olmedo N, et al. Impact of a school-based intervention program on obesity risk factors in Mexican children. Salud Publica Mexico. 2013;55:S374–S87.
189. Sahota P, Christian M, Day R, Cocks K. The feasibility and acceptability of a primary school-based programme targeting diet and physical activity: the PhunkyFoods Programme. Pilot Feasibility Stud. 2019;5:152.
190. Sahota P, Rudolf MCJ, Dixey R, Hill AJ, Barth JH, Cade J. Randomised controlled trial of primary school based intervention to reduce risk factors for obesity. Bmj-Brit Med J. 2001;323(7320):1029–32.
191. Salcedo Aguilar F, Martinez-Vizcaino V, Sanchez Lopez M, Solera Martinez M, Franquelo Gutierrez R, Serrano Martinez S, et al. Impact of an after-school physical activity program on obesity in children. J Pediatr. 2010;157(1):36–42 e3.
192. Sallis JF, Mckenzie TL, Alcaraz JE, Kolody B, Hovell MF, Nader PR. Project Spark - Effects of Physical-Education on Adiposity in Children. Ann Ny Acad Sci. 1993;699:127–36.
193. Salmon J, Arundell L, Cerin E, Ridgers ND, Hesketh KD, Daly RM, et al. Transform-Us! cluster RCT: 18-month and 30-month effects on children's physical activity, sedentary time and cardiometabolic risk markers. Br J Sports Med. 2023;57(5):311–9.
194. Salmon J, Ball K, Hume C, Booth M, Crawford D. Outcomes of a group-randomized trial to prevent excess weight gain, reduce screen behaviours and promote physical activity in 10-year-old children: switch-play. Int J Obes (Lond). 2008;32(4):601–12.
195. Sánchez-Martínez F, Brugueras S, Serral G, Valmayor S, Juárez O, López MJ, et al. Three-Year Follow-Up of the POIBA Intervention on Childhood Obesity: A Quasi-Experimental Study. Nutrients. 2021;13(2).
196. Santos RG, Durksen A, Rabbanni R, Chanoine JP, Lamboo Miln A, Mayer T, et al. Effectiveness of peer-based healthy living lesson plans on anthropometric measures and physical activity in elementary school students: a cluster randomized trial. JAMA Pediatr. 2014;168(4):330–7.
197. Santos-Beneit G, Bodega P, de Cos-Gandoy A, de Miguel M, Rodriguez C, Orrit X, et al. Effect of Time-Varying Exposure to School-Based Health Promotion on Adiposity in Childhood. J Am Coll Cardiol. 2024;84(6):499–508.

198. Scherr RE, Jones Ph DA, Colorafi R, Klisch S, Linnell JD, Soule KE. Assessing the Effectiveness of an Extender Model Partnership in Implementing a Multicomponent, School-Based Nutrition Intervention. Health Promot Pract. 2021;22(6):890–8.
199. Scherr RE, Linnell JD, Dharmar M, Beccarelli LM, Bergman JJ, Briggs M, et al. A Multicomponent, School-Based Intervention, the Shaping Healthy Choices Program, Improves Nutrition-Related Outcomes. J Nutr Educ Behav. 2017;49(5):368–79 e1.
200. Schwartz AE, Leardo M, Aneja S, Elbel B. Effect of a School-Based Water Intervention on Child Body Mass Index and Obesity. JAMA Pediatr. 2016;170(3):220–6.
201. Sevinç Ö, Bozkurt AI, Gündogdu M, Aslan ÜB, Agbuga B, Aslan S, et al. Evaluation of the effectiveness of an intervention program on preventing childhood obesity in Denizli, Turkey. Turkish Journal of Medical Sciences. 2011;41(6):1097–105.
202. Sgambato MR, Cunha DB, Souza BSN, Henriques VT, Rodrigues RRM, Rêgo ALV, et al. Effectiveness of school-home intervention for adolescent obesity prevention: parallel school randomised study. Br J Nutr. 2019;122(9):1073–80.
203. Shamah Levy T, Morales Ruan C, Amaya Castellanos C, Salazar Coronel A, Jimenez Aguilar A, Mendez Gomez Humaran I. Effectiveness of a diet and physical activity promotion strategy on the prevention of obesity in Mexican school children. BMC Public Health. 2012;12:152.
204. Shang X, Li Y, Xu H, Zhang Q, Hu X, Liu A, et al. Effect of multidimensional lifestyle interventions on metabolic risk reduction in children: a cluster randomised controlled trial. Prev Med. 2020;133:106010.
205. Shofan Y, Kedar O, Branski D, Berry E, Wilschanski M. A school-based program of physical activity may prevent obesity. Eur J Clin Nutr. 2011;65(6):768–70.
206. Sichieri R, Paula Trotte A, de Souza RA, Veiga GV. School randomised trial on prevention of excessive weight gain by discouraging students from drinking sodas. Public Health Nutr. 2009;12(2):197–202.
207. Sichieri R, Yokoo EM, Pereira RA, Veiga GV. Water and sugar-sweetened beverage consumption and changes in BMI among Brazilian fourth graders after 1-year follow-up. Public Health Nutr. 2013;16(1):73–7.
208. Siegrist M, Hanssen H, Lammel C, Haller B, Koch AM, Stemp P, et al. Effects of a cluster-randomized school-based prevention program on physical activity and microvascular function (JuvenTUM 3). Atherosclerosis. 2018;278:73–81.
209. Siegrist M, Lammel C, Haller B, Christle J, Halle M. Effects of a physical education program on physical activity, fitness, and health in children: The JuvenTUM project. Scand J Med Sci Spor. 2013;23(3):323–30.
210. Sigmund E, El Ansari W, Sigmundová D. Does school-based physical activity decrease overweight and obesity in children aged 6-9 years? A two-year non-randomized longitudinal intervention study in the Czech Republic. Bmc Public Health. 2012;12.
211. Sigmund E, Sigmundova D. Longitudinal 2-year follow-up on the effect of a non-randomised school-based physical activity intervention on reducing overweight and obesity of Czech children aged 10-12 years. Int J Environ Res Public Health. 2013;10(8):3667–83.
212. Smith LH, Holloman C. Comparing the effects of teen mentors to adult teachers on child lifestyle behaviors and health outcomes in Appalachia. J Sch Nurs. 2013;29(5):386–96.
213. Speroni KG, Earley C, Atherton M. Evaluating the effectiveness of the Kids Living Fit program: a comparative study. J Sch Nurs. 2007;23(6):329–36.
214. Spiegel SA, Foulk D. Reducing overweight through a multidisciplinary school-based intervention. Obesity. 2006;14(1):88–96.
215. Stea TH, Tveter ET, Te Velde SJ, Vik FN, Klepp KI, Bere E. The effect of an extra piece of fruit or vegetables at school on weight status in two generations - 14 years follow-up of the Fruit and Vegetables Makes the Marks study. PLoS One. 2018;13(10):e0205498.
216. Martinez-Vizcaino V, Soriano-Cano A, Garrido-Miguel M, Cavero-Redondo I, Medio EP, Madrid VM, et al. The effectiveness of a high-intensity interval games intervention in schoolchildren: A cluster-randomized trial. Scand J Med Sci Sports. 2022;32(4):765–81.
217. Story M, Sherwood NE, Himes JH, Davis M, Jacobs DR, Jr., Cartwright Y, et al. An after-school obesity prevention program for African-American girls: the Minnesota GEMS pilot study. Ethn Dis. 2003;13(1 Suppl 1):S54–64.

218. Gutin B, Yin Z, Johnson M, Barbeau P. Preliminary findings of the effect of a 3-year after-school physical activity intervention on fitness and body fat: the Medical College of Georgia Fitkid Project. Int J Pediatr Obes. 2008;3 Suppl 1:3–9.
219. Simon C, Kellou N, Dugas J, Platat C, Copin N, Schweitzer B, et al. A socio-ecological approach promoting physical activity and limiting sedentary behavior in adolescence showed weight benefits maintained 2.5 years after intervention cessation. Int J Obes (Lond). 2014;38(7):936–43.
220. Simon C, Schweitzer B, Oujaa M, Wagner A, Arveiler D, Triby E, et al. Successful overweight prevention in adolescents by increasing physical activity: a 4-year randomized controlled intervention. Int J Obes (Lond). 2008;32(10):1489–98.
221. Morais LDC, Paravidino VB, Mediano MFF, Benthroldo R, Goncalves TR, Sgambato MR, et al. Effectiveness of a school-based randomized controlled trial aimed at increasing physical activity time in adolescents. Eur J Public Health. 2021;31(2):367–72.
222. Stock S, Miranda C, Evans S, Plessis S, Ridley J, Yeh S, et al. Healthy Buddies: a novel, peer-led health promotion program for the prevention of obesity and eating disorders in children in elementary school. Pediatrics. 2007;120(4):e1059–68.
223. Tarro L, Llauradó E, Albaladejo R, Moriña D, Arija V, Solà R, et al. A primary-school-based study to reduce the prevalence of childhood obesity - the EdAl (Educacio en Alimentacio) study: a randomized controlled trial. Trials. 2014;15.
224. Taylor RW, McAuley KA, Barbezat W, Farmer VL, Williams SM, Mann JI, et al. Two-year follow-up of an obesity prevention initiative in children: the APPLE project. Am J Clin Nutr. 2008;88(5):1371–7.
225. Taylor RW, McAuley KA, Barbezat W, Strong A, Williams SM, Mann JI. APPLE Project: 2-y findings of a community-based obesity prevention program in primary school-age children. American Journal of Clinical Nutrition. 2007;86(3):735–42.
226. Taylor RW, McAuley KA, Williams SM, Barbezat W, Nielsen G, Mann JI. Reducing weight gain in children through enhancing physical activity and nutrition: the APPLE project. Int J Pediatr Obes. 2006;1(3):146–52.
227. Telford RD, Cunningham RB, Fitzgerald R, Olive LS, Prosser L, Jiang X, et al. Physical education, obesity, and academic achievement: a 2-year longitudinal investigation of Australian elementary school children. Am J Public Health. 2012;102(2):368–74.
228. Teo CH, Chin YS, Lim PY, Masrom SAH, Shariff ZM. Impacts of a School-Based Intervention That Incorporates Nutrition Education and a Supportive Healthy School Canteen Environment among Primary School Children in Malaysia. Nutrients. 2021;13(5).
229. Thivel D, Isacco L, Lazaar N, Aucouturier J, Ratel S, Dore E, et al. Effect of a 6-month school-based physical activity program on body composition and physical fitness in lean and obese schoolchildren. Eur J Pediatr. 2011;170(11):1435–43.
230. Tran BX, Ohinmaa A, Kuhle S, Johnson JA, Veugelers PJ. Life course impact of school-based promotion of healthy eating and active living to prevent childhood obesity. PLoS One. 2014;9(7):e102242.
231. Treu JA, Doughty K, Reynolds JS, Njike VY, Katz DL. Advancing School and Community Engagement Now for Disease Prevention (ASCEND). Am J Health Promot. 2017;31(2):143–52.
232. Tucker S, Lanningham-Foster L, Murphy J, Olsen G, Orth K, Voss J, et al. A school based community partnership for promoting healthy habits for life. J Community Health. 2011;36(3):414–22.
233. van den Berg A, Warren JL, McIntosh A, Hoelscher D, Ory MG, Jovanovic C, et al. Impact of a gardening and physical activity intervention in Title 1 schools: The TGEG study. Childhood Obesity. 2020;16(Suppl 1):44–54.
234. Verjans-Janssen SRB, Van Kann DHH, Gerards S, Vos SB, Jansen MWJ, Kremers SPJ. Study protocol of the quasi-experimental evaluation of "KEIGAAF": a context-based physical activity and nutrition intervention for primary school children. BMC Public Health. 2018;18(1):842.
235. Vieira M, Teixeira A, Carvalho GS. Effectiveness of the "Planning Health in School" Programme on Children's Nutritional Status. Int J Environ Res Public Health. 2021;18(23).

236. Viggiano E, Viggiano A, Di Costanzo A, Viggiano A, Viggiano A, Andreozzi E, et al. Healthy lifestyle promotion in primary schools through the board game Kaledo: a pilot cluster randomized trial. Eur J Pediatr. 2018;177(9):1371–5.
237. Wadolowska L, Hamulka J, Kowalkowska J, Ulewicz N, Hoffmann M, Gornicka M, et al. Changes in Sedentary and Active Lifestyle, Diet Quality and Body Composition Nine Months after an Education Program in Polish Students Aged 11(-)12 Years: Report from the ABC of Healthy Eating Study. Nutrients. 2019;11(2).
238. Walther C, Gaede L, Adams V, Gelbrich G, Leichtle A, Erbs S, et al. Effect of increased exercise in school children on physical fitness and endothelial progenitor cells: a prospective randomized trial. Circulation. 2009;120(22):2251–9.
239. Wang X, Liu J, Gao D, Li Y, Ma Q, Chen L, et al. Effectiveness of national multicentric school-based health lifestyles intervention among chinese children and adolescents on knowledge, belief, and practice toward obesity at individual, family and schools' levels. Front Pediatr. 2022;10:917376.
240. Wang Z, Xu F, Ye Q, Tse LA, Xue H, Tan Z, et al. Childhood obesity prevention through a community-based cluster randomized controlled physical activity intervention among schools in china: the health legacy project of the 2nd world summer youth olympic Games (YOG-Obesity study). Int J Obes (Lond). 2018;42(4):625–33.
241. Warren JM, Henry CJ, Lightowler HJ, Bradshaw SM, Perwaiz S. Evaluation of a pilot school programme aimed at the prevention of obesity in children. Health Promot Int. 2003;18(4):287–96.
242. Waters E, Gibbs L, Tadic M, Ukoumunne OC, Magarey A, Okely AD, et al. Cluster randomised trial of a school-community child health promotion and obesity prevention intervention: findings from the evaluation of fun 'n healthy in Moreland! BMC Public Health. 2017;18(1):92.
243. Weber KS, Eitner J, Dauben L, Spörkel O, Strassburger K, Sommer J, et al. Positive Effects of Practical Nutrition Lessons in a Primary School Setting with a High Proportion of Migrant School Children. Exp Clin Endocrinol Diabetes. 2020;128(2):111–8.
244. Weber KS, Sporkel O, Mertens M, Freese A, Strassburger K, Kemper B, et al. Positive Effects of Promoting Physical Activity and Balanced Diets in a Primary School Setting with a High Proportion of Migrant School Children. Exp Clin Endocrinol Diabetes. 2017;125(8):554–62.
245. Wendel ML, Benden ME, Zhao H, Jeffrey C. Stand-Biased Versus Seated Classrooms and Childhood Obesity: A Randomized Experiment in Texas. Am J Public Health. 2016;106(10):1849–54.
246. Widhalm K, Helk O, Pachinger O. The Viennese EDDY Study as a Role Model for Obesity: Prevention by Means of Nutritional and Lifestyle Interventions. Obes Facts. 2018;11(3):247–56.
247. Willeboordse M, Bartelink NHM, van Assema P, Kremers SPJ, Savelberg H, Hahnraths MTH, et al. Battling the obesity epidemic with a school-based intervention: Long-term effects of a quasi-experimental study. PLoS One. 2022;17(9):e0272291.
248. Williamson DA, Champagne CM, Harsha DW, Han H, Martin CK, Newton RL, Jr., et al. Effect of an environmental school-based obesity prevention program on changes in body fat and body weight: a randomized trial. Obesity (Silver Spring). 2012;20(8):1653–61.
249. Williamson DA, Copeland AL, Anton SD, Champagne C, Han H, Lewis L, et al. Wise mind project: A school-based environmental approach for preventing weight gain in children. Obesity. 2007;15(4):906–17.
250. Wolfe WS, Dollahite J. Evaluation of the Choose Health: Food, Fun, and Fitness 3rd- to 6th-Grade Curriculum: Changes in Obesity-Related Behaviors. J Sch Health. 2021;91(1):9–18.
251. Wright K, Giger JN, Norris K, Suro Z. Impact of a nurse-directed, coordinated school health program to enhance physical activity behaviors and reduce body mass index among minority children: a parallel-group, randomized control trial. Int J Nurs Stud. 2013;50(6):727–37.
252. Wright K, Norris K, Newman Giger J, Suro Z. Improving healthy dietary behaviors, nutrition knowledge, and self-efficacy among underserved school children with parent and community involvement. Child Obes. 2012;8(4):347–56.
253. Wright K, Suro Z. Using community--academic partnerships and a comprehensive school-based program to decrease health disparities in activity in school-aged children. J Prev Interv Community. 2014;42(2):125–39.

254. Xu F, Ware RS, Leslie E, Tse LA, Wang Z, Li J, et al. Effectiveness of a Randomized Controlled Lifestyle Intervention to Prevent Obesity among Chinese Primary School Students: CLICK-Obesity Study. PLoS One. 2015;10(10):e0141421.
255. Xu H, Ecker O, Zhang Q, Du S, Liu A, Li Y, et al. The effect of comprehensive intervention for childhood obesity on dietary diversity among younger children: Evidence from a school-based randomized controlled trial in China. PLoS One. 2020;15(7):e0235951.
256. Xu H, Li Y, Shang X, Du S, Zhang Q, Liu A, et al. Effect of Comprehensive Interventions Including Nutrition Education and Physical Activity on High Blood Pressure among Children: Evidence from School-Based Cluster Randomized Control Trial in China. Int J Environ Res Public Health. 2020;17(23).
257. Xu H, Li Y, Zhang Q, Hu XL, Liu A, Du S, et al. Comprehensive school-based intervention to control overweight and obesity in China: a cluster randomized controlled trial. Asia Pac J Clin Nutr. 2017;26(6):1139–51.
258. Yang Y, Kang B, Lee EY, Yang HK, Kim HS, Lim SY, et al. Effect of an obesity prevention program focused on motivating environments in childhood: a school-based prospective study. Int J Obes (Lond). 2017;41(7):1027–34.
259. Yin Z, Moore JB, Johnson MH, Vernon MM, Gutin B. The impact of a 3-year after-school obesity prevention program in elementary school children. Child Obes. 2012;8(1):60–70.
260. Zammit N, Maatoug J, Ghammam R, Raoudha K, Lamia B, Mustafa A, et al. Sustainability of the results of a non communicable diseases prevention program among youth in the region of Sousse, Tunisia. Int J Adolesc Med Health. 2016;30(4).
261. Zhou H-R, Wang W-W, Yang H-F, Qi S-X, Wang C-C, Hong X. The effects of a one year school-based comprehensive lifestyle intervention among Chinese kids against obesity (CLICK-obesity) in Nanjing, China. Journal of Public Health. 2021;31(7):1071–9.
262. Yin Z, Gutin B, Johnson MH, Hanes J, Jr., Moore JB, Cavnar M, et al. An environmental approach to obesity prevention in children: Medical College of Georgia FitKid Project year 1 results. Obes Res. 2005;13(12):2153–61.

Supplementary File 4. Reported outcomes, frequencies and summary description

Outcome domain	Reported (merged) outcomes	Frequ
Academic skills - including outcomes related to skills that help students succeeding in academic settings		
	Reading skills	3
	Mathematics skills	3
	Academic attainment, overall	1
	Academic attainment, math	1
	Academic attainment, reading	1
	Academic attainment, writing	1
	Writing skills	2
	Oral language skills	1
	Critical analysis	1
	Expression skills	1
	School grades	1
	Science process skills	1
	Classroom behaviour	2
	Academic performance	4
	Cognitive performance	4
	Sternberg paradigm	2
	Attention	2
Anthropometric measurements domain - including outcomes related to physical properties of the body		
	BMI	12
	BMI-z	10
	BMI percentile	31
	BMI binominal (increase/decrease)	1
	BMI categories	10
	Shifts in weight categories	1
	Odds for overweight/obesity	1
	Incidence overweight/obesity	12
	Remission overweight/obesity	8
	Prevalece of stunting	1
	Incidence of stunting	1
	Incidence of wasting	1
	Admission rate	1
	Height	50
	Sitting height	2
	Weight z-score	6
	Weight	51
	Waist circumference	80
	Waist circumference z-score	5
	Hip circumference	7
	Arm circumference	1
	Hip-to-waist ratio	1
	Waist-to-hip ratio	7
	Waist-to-height ratio	22
	Skinfold thickness	40
	Body fat	67
	% abdominal obesity	2
	Trunk fat	5
	Trunkal fat free mass	1
	Peripheral fat	3
	Lean body mass	16
	Muscle mass	5
	Fat free mass index	3
	Fat mass index	3
	Fat ratio	1
	Bone mass	1
	Bone mineral content	6
	Bone mineral density (femoral neck, lumbar spine)	9
	Tibial total content 4%	1
	Tibial trabecular density 4%	1
	Tibial cortical density 38%	1
	Radial total content 4%	1
	Radial trabecular density 4%	1
	Radial cortical density 38%	1
	Stiffnes index, calceneous %	2
	Broadbent ultrasound attenuation	2
	Bioelectrical impedance	2
	Total body density	1
	Total body water	2
	Maturity	2
Dietary behaviours - including outcomes related to food choice, eating behaviour and dietary intake/nutrition		
	Total energy intake	21
	Total energy intake/kg	1
	Energy intake from sugar	1
	Energy intake from fat	1
	Energy selection	1
